# A scoping review on tools and methods for trait prioritization in crop breeding programmes

**DOI:** 10.1038/s41477-024-01639-6

**Published:** 2024-02-22

**Authors:** M. Occelli, R. Mukerjee, C. Miller, J. Porciello, S. Puerto, E. Garner, M. Guerra, M. I. Gomez, H. A. Tufan

**Affiliations:** 1 School of Integrative Plant Science, Cornell University, Ithaca, NY USA; 2 Charles H. Dyson School of Applied Economics and Management, Cornell University, Ithaca, NY USA; 3 International Food Policy Research Institute, Washington, DC USA; 4 Industrial and Labor Relations School, Cornell University, Ithaca, NY USA; 5 Lucy Family Institute for Data and Society, University of Notre Dame, Notre Dame, IN USA; 6 Center for International Forestry Research (CIFOR) and World Agroforestry (ICRAF), Bogor, Indonesia

**Keywords:** Plant breeding, Agriculture

## Abstract

Trait prioritization studies have guided research, development and investment decisions for public-sector crop breeding programmes since the 1970s, but the research design, methods and tools underpinning these studies are not well understood. We used PRISMA-ScR (Preferred Reporting Items for Systematic review and Meta-Analysis Protocols) to evaluate research on trait ranking for major crops over the past 40 years (1980–2023). Data extraction and descriptive analysis on 657 papers show uneven attention to crops, lack of systematic sex disaggregation and regional bias. The lack of standardized trait data taxonomy across studies, and inconsistent research design and data collection practices make cross-comparison of findings impossible. In addition, network mapping of authors and donors shows patterns of concentration and the presence of silos within research areas. This study contributes to the next generation of innovation in trait preference studies to produce more inclusive, demand-driven varietal design that moves beyond trait prioritization focused on productivity and yield.

## Abstract (web summary)

Trait prioritization studies have informed crop breeding programmes for decades. This scoping review identifies broad crop coverage, systematic sex disaggregation and reduced regional bias as priorities for more inclusive, demand-driven initiatives.

## Main

Public-sector crop breeding programmes play a central role in ensuring food security around the world. These programmes are responsible for research and development on a variety of crops, reflecting the needs and preferences of a diverse population of growers as well as processors, traders and consumers. Public-sector crop breeding programmes are increasingly becoming demand-led and data driven^1^, starting with product design, which requires understanding of trait preferences of heterogeneous target populations in a given environment. Trait preferences are then formalized through target product profiles to guide breeding^2,3^.

Studying trait preferences initially requires definition of what is meant by the term ‘trait’. This varies from definitions rooted in functionality in an evolutionary fitness sense^4^ to narrower definitions of inherited and measurable crop characteristics that increase yield or alleviate biotic and abiotic stresses. Traits help define a variety and distinguish it from other varieties in a market segment^3^. Here we define the term ‘trait’ in a broad sense as ‘observable crop characteristics that can be identified by stakeholders’, or simply, phenotypic variables of a crop (https://cropontology.org/about).

The process through which a wide range of crop attributes are identified and translated into actionable breeding decisions is called trait prioritization^5^. Studies that engage stakeholders in a crop value chain to generate data for trait prioritization are called trait prioritization studies. Trait prioritization studies are particularly important for crop breeders targeting marginalized smallholder farmers who are seldom directly engaged in providing input into breeding programme decision-making for varietal design^5^. Releasing a variety with a suite of traits that does not reflect end-user preferences leads to lower adoption^6,7^. Trait prioritization studies therefore hold potential for breeders to overcome the stubbornly low adoption rates for key food security crops^8^. These studies also help donors and institutional leadership guide their strategic goals and investments.

Despite the centrality of trait prioritization in guiding varietal development with higher adoption potential, the methodologies that underpin this work are seldom examined. Crop trait prioritization studies have historically relied on eliciting farmer preferences using direct ranking or choice experimentation^6,9,10^. In addition, decades of plant breeding and varietal selection relying on participatory methods have also yielded rich trait prioritization information across crops and continents^11,12^. Trait prioritization studies are multidisciplinary or interdisciplinary, which adds complexity to research design and implementation^13^.

We sought to identify best practice as well as most effective tools and methods for trait prioritization. Recent research found systematic bias to socio-economic differences in trait preference studies; for example, only 25% of studies reported sex-disaggregated trait preference data^14^. This is despite mounting evidence on the impact of how social differences such as household food security, poverty and gender intersect to shape differential crop trait preferences^13–15^. A scoping review of stakeholder preference studies focused on rice^16^ reveals important gaps in stakeholder representation and heterogeneity in trait preferences, but it lacks focus on the robustness of tools and methods utilized to collect trait data. We hypothesize that a lack of systematic attention by researchers to methods and tools for collecting and analysing trait prioritization data hinders good quality, unbiased and representative data collection, which in turn results in public-sector crop breeding programmes’ inability to make data-driven decisions. The aim of our scoping review is to fill this gap in the literature by generating a comprehensive and critical review of crop trait prioritization tools and methods, serving as a resource for crop breeding programmes to build on for research design.

## Results

### Studies are unevenly distributed geographically

**Fig. 1:**
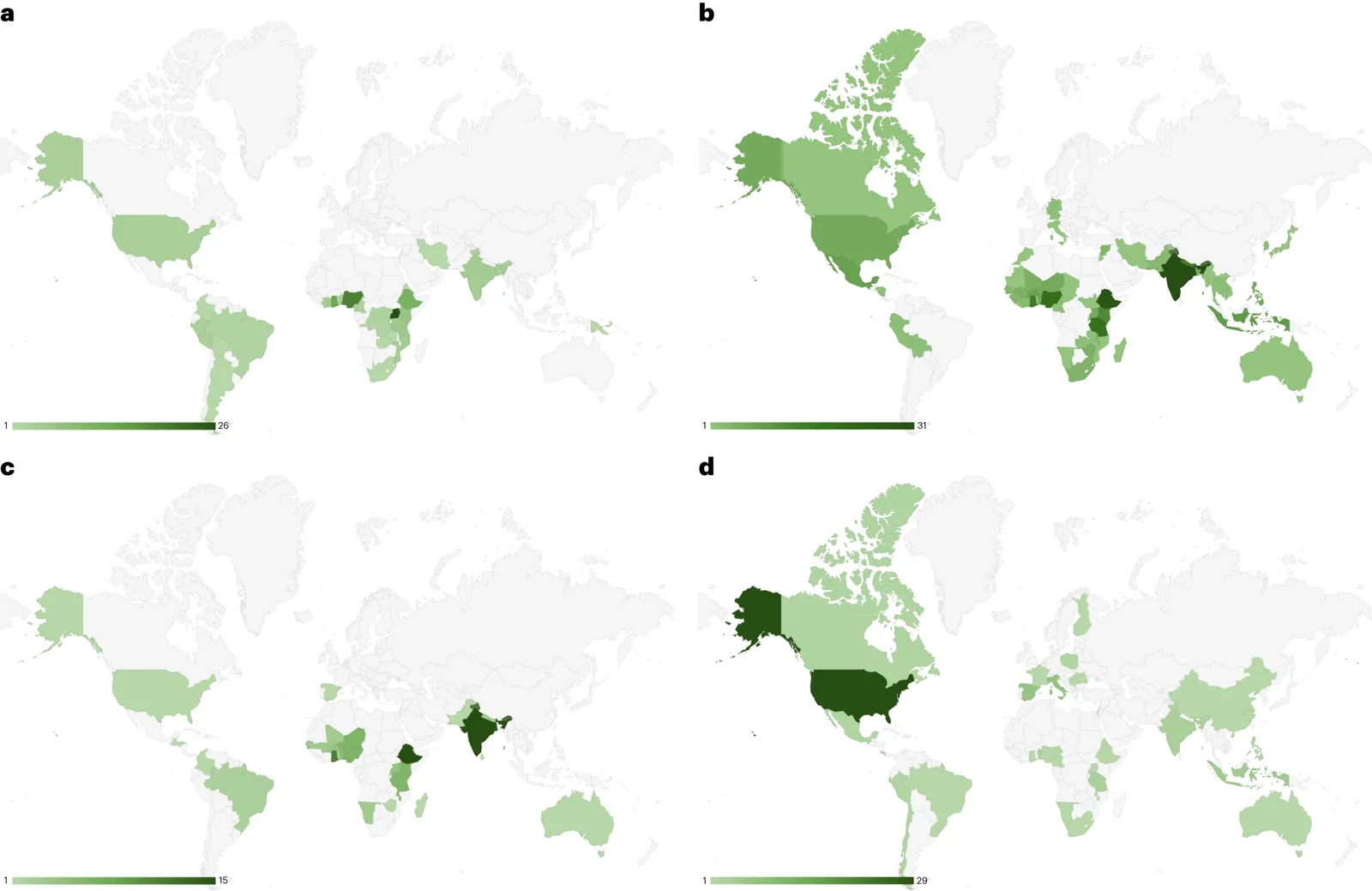
Map of evidence distribution by country and by crop group (1980–2023). **a**, RTB. **b**, Cereals. **c**, Legumes. **d**, Vegetables and fruits. The bars below each figure represent the total number of studies for each group in each country, with the shading indicating the relative intensity of studies for that country.

**Extended Data Fig. 1:**
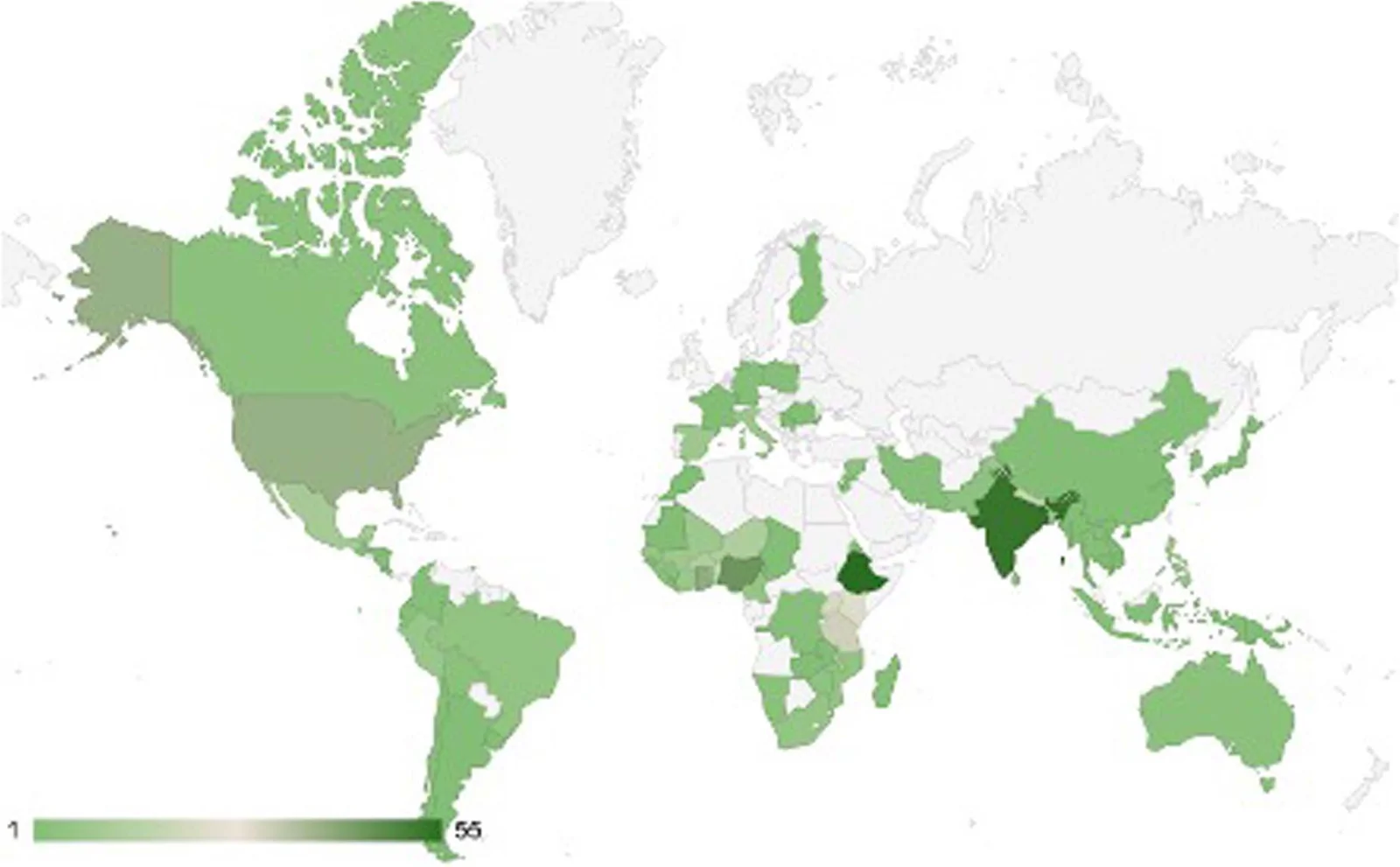
Map of evidence distribution by country (1980–2023). The bar below the map represent the total number of studies in each country, the shading indicative of the relative intensity of studies for that country.

A total of 564 original studies met the inclusion criteria. Of these studies, 83% were peer-reviewed articles and 17% were from grey literature sources. More than 50% of the research on trait prioritization has been published since 2016. Figure 1 shows the map of evidence distribution by country and by crop group. Ethiopia and India have the highest number of included studies, followed at a distance by Nigeria, Ghana, USA and Uganda. A large share of the studies (57%) took place in sub-Saharan Africa, while studies in South America account for only 6% of the total. We found that studies conducted in the Middle East and North Africa comprise only 2% of our total sample. Studies analysing trait priorities for root–tuber–bananas (RTB), cereal and legume crops are almost exclusively located in South Asia, sub-Saharan Africa and South America (96%), while trait priorities for fruits and vegetables are predominantly (59%) studied in North America and Europe. Extended Data Fig. 1 shows the global evidence distribution by country.

### Surveys and descriptive analyses drive trait prioritization

**Fig. 2:**
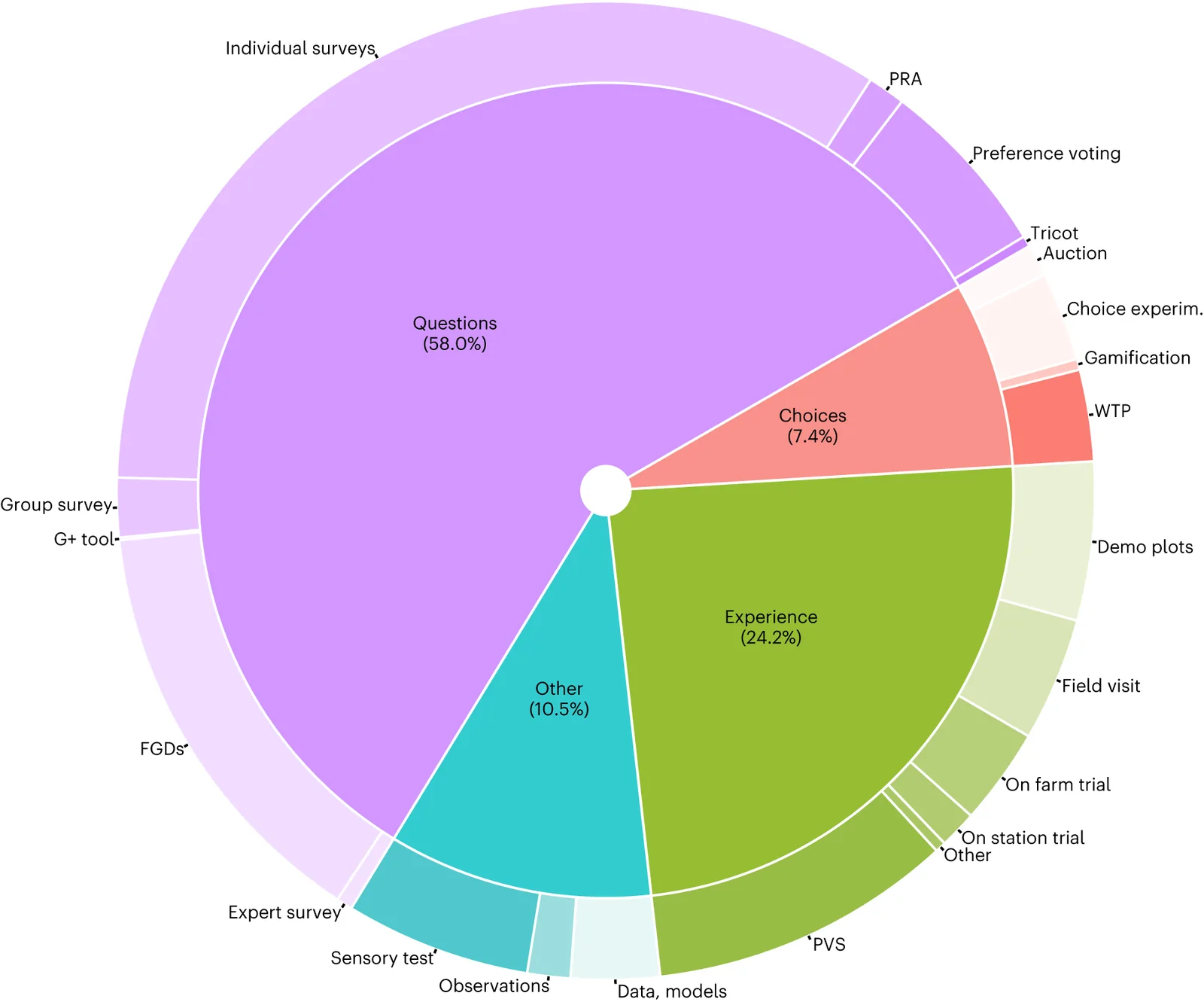
Summary of tools that researchers use to gather end-users’ feedback on trait priorities. The inner ring outlines the four broad categories to which the 21 tools are mapped. The outer ring shows the tools within each broad category that were most frequently mentioned across the included studies. The relative area occupied by categories indicates their relevance. Full data and frequencies for each category are presented in Supplementary Table 1. PRA, participatory rural appraisal; WTP, willingness-to-pay; FGDs, focus group discussions.

**Fig. 3:**
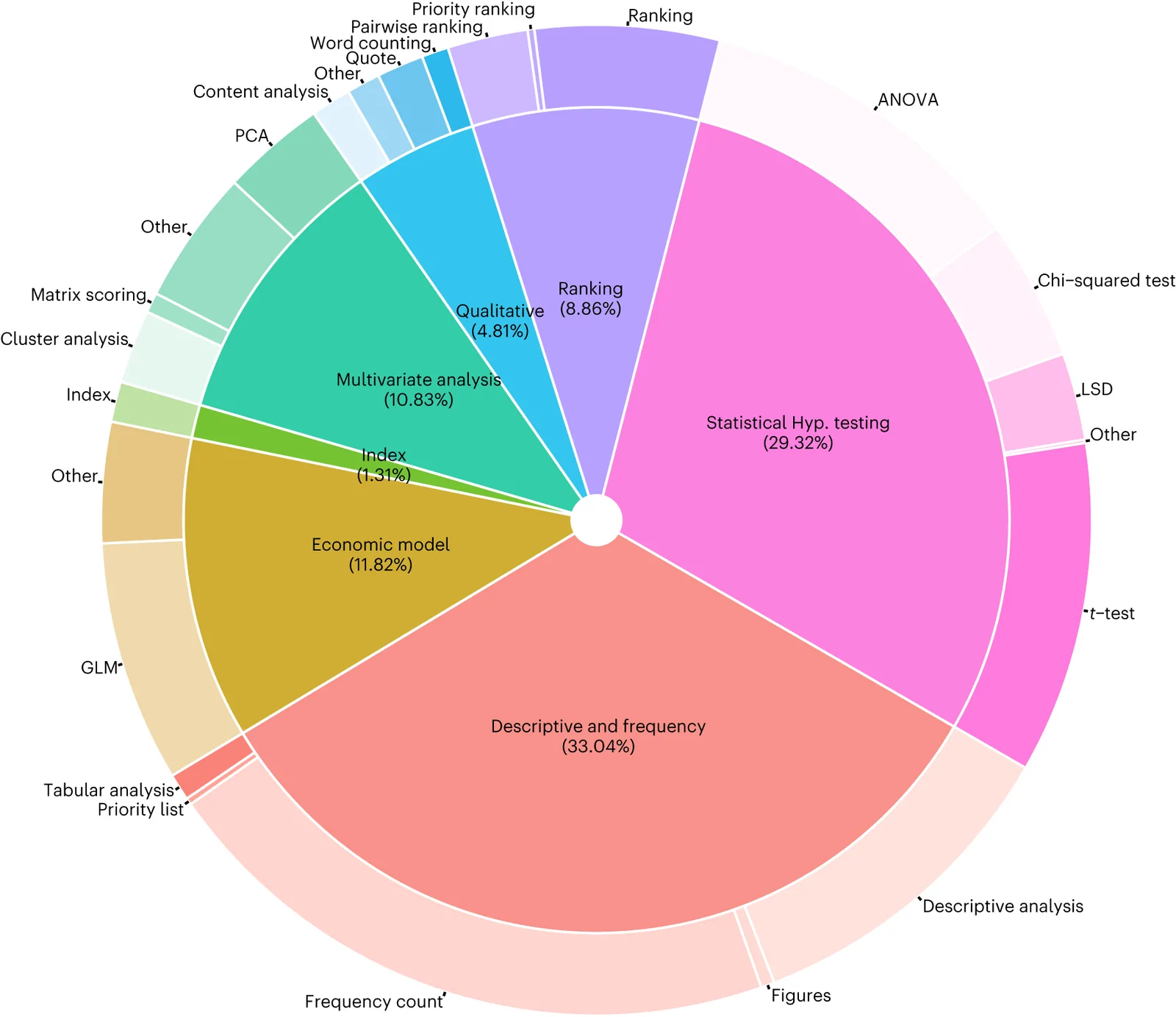
Summary of analytical approaches for trait prioritization. The inner ring outlines the seven broad categories to which the 24 methods are mapped. The outer ring shows the methods within each broad category that were most frequently mentioned across the included studies. The relative area occupied by categories indicates their relevance. Full data and frequencies for each category are presented in Supplementary Table 2. ANOVA, analysis of variance; LSD, least significant difference; GLM, generalized linear model; PCA, principle component analysis.

To collect trait data, a majority (58%) of studies identify preferred traits through direct questions (Fig. 2), including individual surveys and focus group discussions. Trait prioritization through direct experience comes second (24%), including participatory varietal selection (PVS) and demonstration plots. Around 10% of the trait preferences are collected using choice experiments and sensory evaluations (see category Other). The number of tools used within each study varies. More than a third of the studies report using only one tool for trait prioritization, with individual surveys (33%), PVS (14%) or focus group discussions (10%) being the most used. Another third employ two tools, combining individual surveys with focus group discussions or with PVS. The use of more than one tool is more frequent in recent studies. Peer-reviewed articles have a median of two tools per study compared with the median of one tool for studies in the grey literature. To prioritize collected trait data, frequency counts and hypothesis testing are most often used, followed by economic modelling, multivariate analyses and ranking (Fig. 3). Only 5% of the studies used qualitative data analysis, even though one of the most commonly used data collection tools are focus group discussions that require qualitative analysis. Few papers are mixed-method studies combining descriptive methods and hypothesis testing (13%), followed by even fewer mixed-method studies employing descriptive methods and economic modelling (6%).

### Trait prioritization studies are mainly formally led

**Extended Data Fig. 2:**
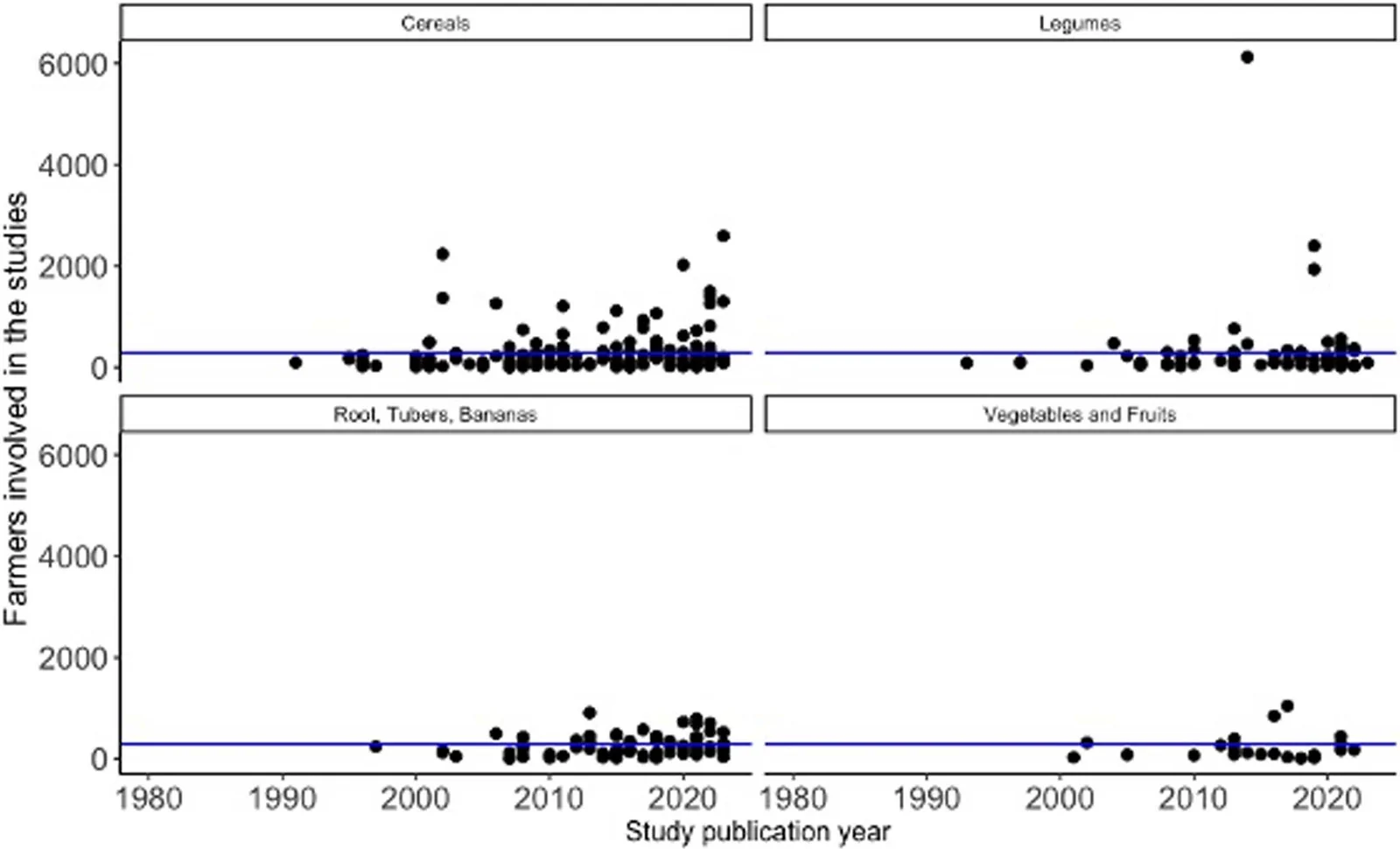
**Farmers’ engagement in the studies sampled, by crop and over time**.

Trait prioritization studies on cereals tend to be more participatory (54% of the studies), while studies on legumes (51%) and RTB crops (41%) are less so. Participatory research is uncommon (14%) on vegetables and fruits. Looking more closely at respondents and how they were engaged, we found that information on sample size and sampling procedure is highly variable and lack robustness. Of the studies, 12% fail to report the number of respondents engaged, while 52% exclusively involve farmers without specifying whether they are also consumers or producers. Less than 19% of the studies involve consumers or traders or other actors in the seed system. We also observe a normalized approach of sampling roughly 250 respondents for trait preference studies, irrespective of crop group, country or method deployed (Extended Data Fig. 2). Triangulating authors’ self-reporting of participatory methodology in studies, we classified the studies as farmer-led or formal-led and consultative or collaborative (see Table 1 in Methods). A vast majority (90%) of the studies that self-report as participatory are classified as formal-led. Only 12.5% of the self-reported participatory studies are formal-led collaborative breeding experiments, with farmers testing varieties in their own field and with their own management practices.

### Gender bias limits data on trait priorities of women farmers

**Fig. 4:**
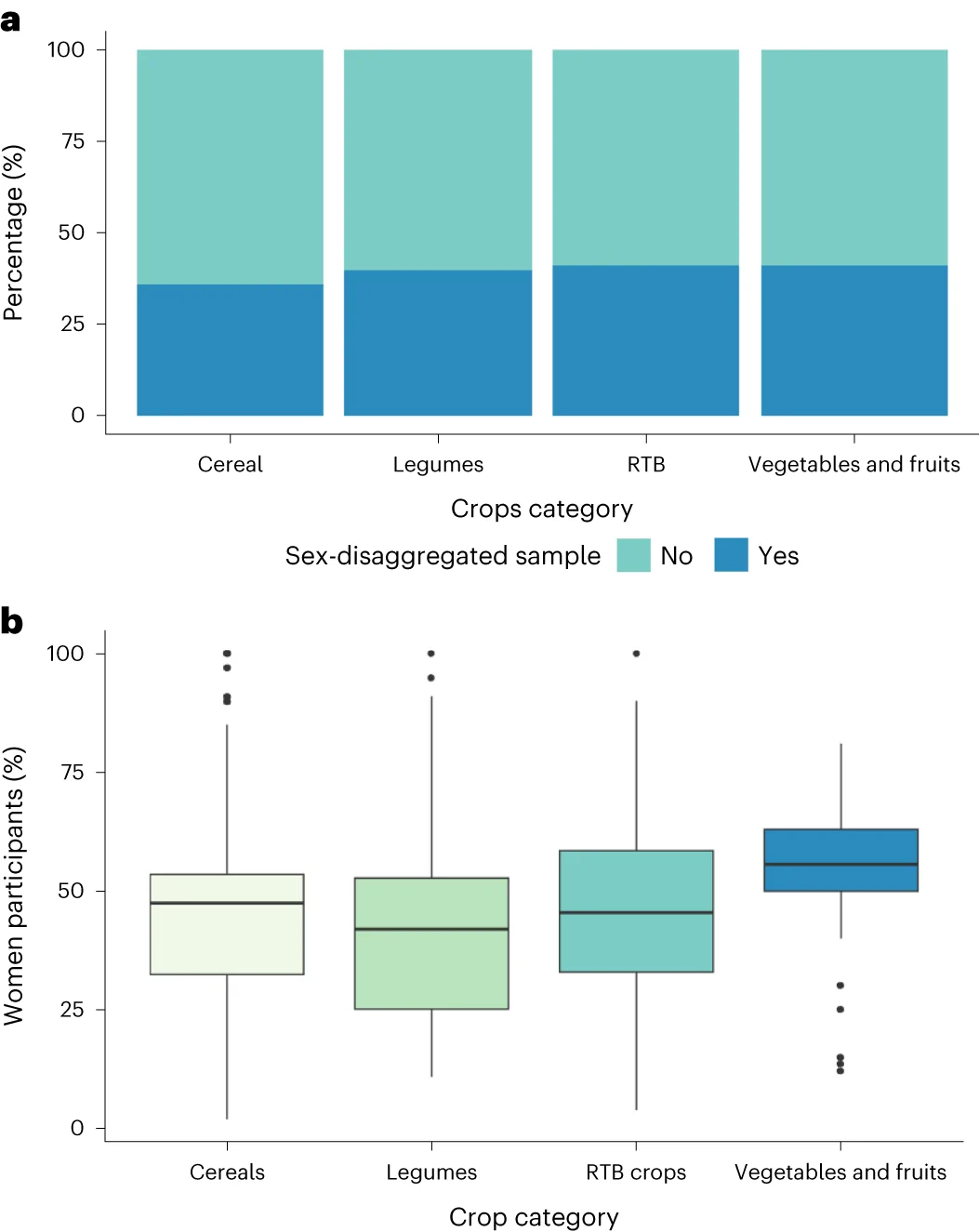
Sex-disaggregated data on trait prioritization. **a**, Studies collecting sex-disaggregated data on preferences. **b**, Percentage of women respondents in the subset of studies collecting sex-disaggregated data. In **b**, the sample is 216 studies. The boxplots indicate the median (horizontal line), the interquartile range from 25% to 75% (lower and upper box limits) and the standard deviation (whiskers); the minimum value is 0, the maximum value is 100, the median is 49.50%, the first quartile is 32% and the third quartile is 57%.

Only 37% of the studies collect sex-disaggregated data on trait preferences. Recent studies sex-disaggregate data more frequently, with a median publication year of 2016 for sex-disaggregated and 2012 for non-disaggregated data. Sex-disaggregation is more common in trait prioritization studies on RTB crops, legumes, and vegetables and fruits, compared with cereals (Fig. 4a). Among studies that sex-disaggregate, studies on vegetables and fruits showed higher numbers of women engaged, while research on legumes showed slightly lower numbers (Fig. 4b). Only 1.7% of studies collected intrahousehold trait preference data.

Of the studies that collect sex-disaggregated data on trait preferences, 84% find sex-based trait differences. Seventy per cent of the studies that show sex-based differences in preferences rely on previous findings from the literature (40%) or additional qualitative (31%) or quantitative (25%) methods within the same study to explain these differences. In discussing sex-disaggregated results, authors use gendered roles to explain them. Characteristics such as ease of peeling, higher nutrient content, culinary attributes, richness in vitamins and postharvest qualities are explained by using stereotypical descriptions of women’s roles in food processing and preparation. Few studies considered other social demographic factors for disaggregating respondents’ preferences. Of the studies, 37% disaggregate data by state or region, 3% disaggregate data by both region and gender, and only 0.9% disaggregate preferences by age.

### Lack of standard taxonomy hinders synthesis of trait data

**Extended Data Fig. 3:**
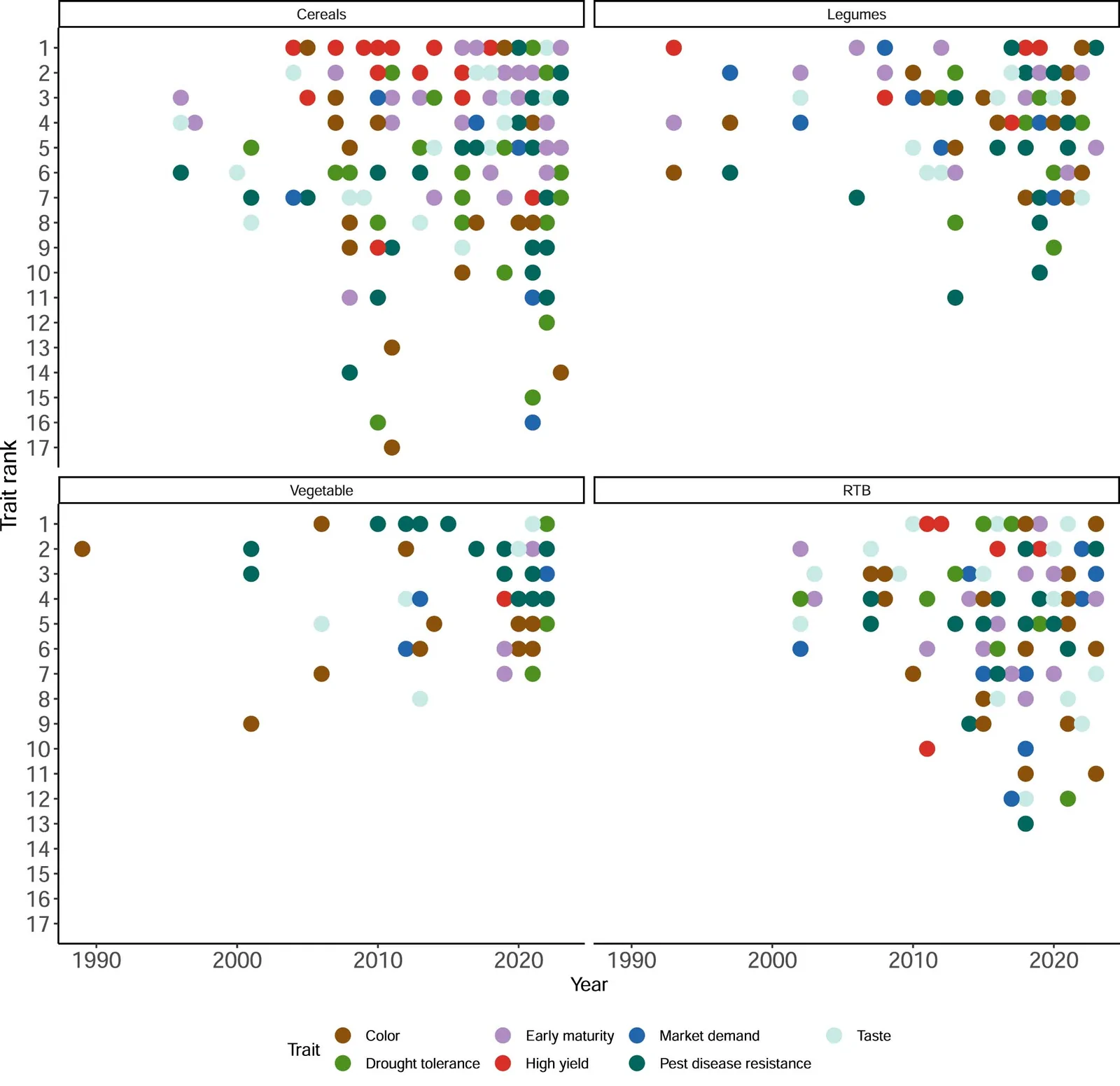
**Ranking position for six of the most common traits (high yield, pest and disease resistance, drought tolerance, market demand, color, taste and early maturity) across time and by crop group**.

We explored the evolution of trait priorities by plotting the position of seven highly recurring traits (high yield, pest and disease resistance, drought tolerance, market demand, taste, early maturity and colour) across time and crop groups (Extended Data Fig. 3). Unsurprisingly, we observe the importance of high yield. However, we also observe heterogeneity in its importance; for example, high yield is less important in RTB crops and vegetables and fruits than in cereals. There is an increase in the importance of early maturity, pest and disease resistance, and drought tolerance in cereals. Taste ranks high among RTB crops, while pest and disease resistance rank high in vegetable and fruit crops. Supplementary Table 4 details the top-three ranked traits for three specific crops in each crop group by decade.

While generating Extended Data Fig. 3 and Supplementary Table 4, we observed a high level of trait description heterogeneity within crops, with no established trait taxonomy process followed. To explore this further, we extracted an illustrative subsample of studies focused on rice traits in India in three consecutive years (Supplementary Table 3). Despite the similarities in the typology of these studies (all non-participatory, no sex-disaggregated data collection), and even shared authorship and study methods, all three studies yielded separate trait priority lists, emphasizing the difficulties in synthesizing useful data from these studies.

### Author and donor networks reveal research and funding silos

**Fig. 5:**
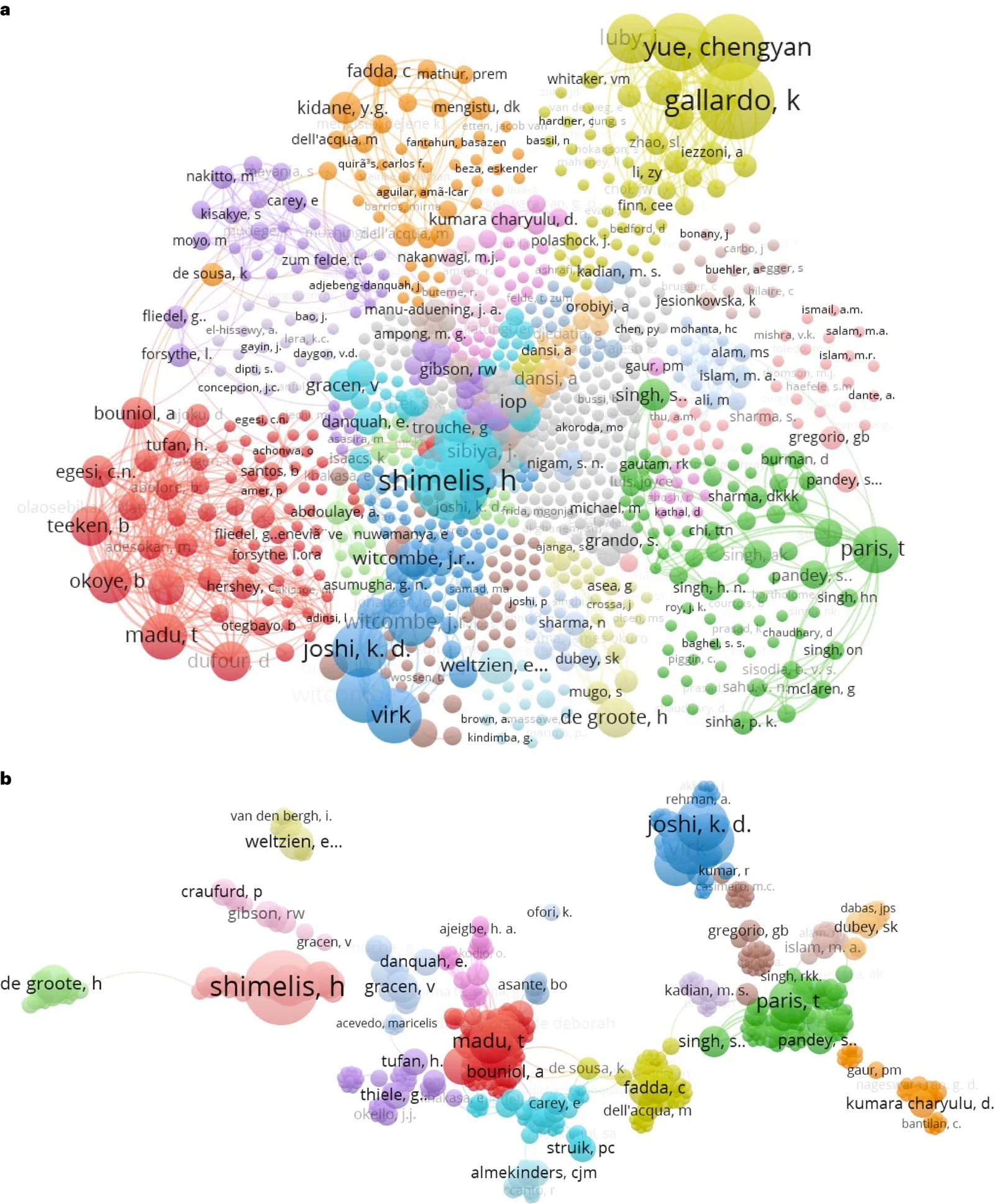
Co-authorship network. The size of the nodes indicates higher numbers of collaborations. Edges indicate co-authorship. Nodes are grouped into clusters that are distinguished by colour. **a**, The network with all authors included. **b**, The network with only authors who are part of an edge (that is, show connection) included.

**Extended Data Fig. 4:**
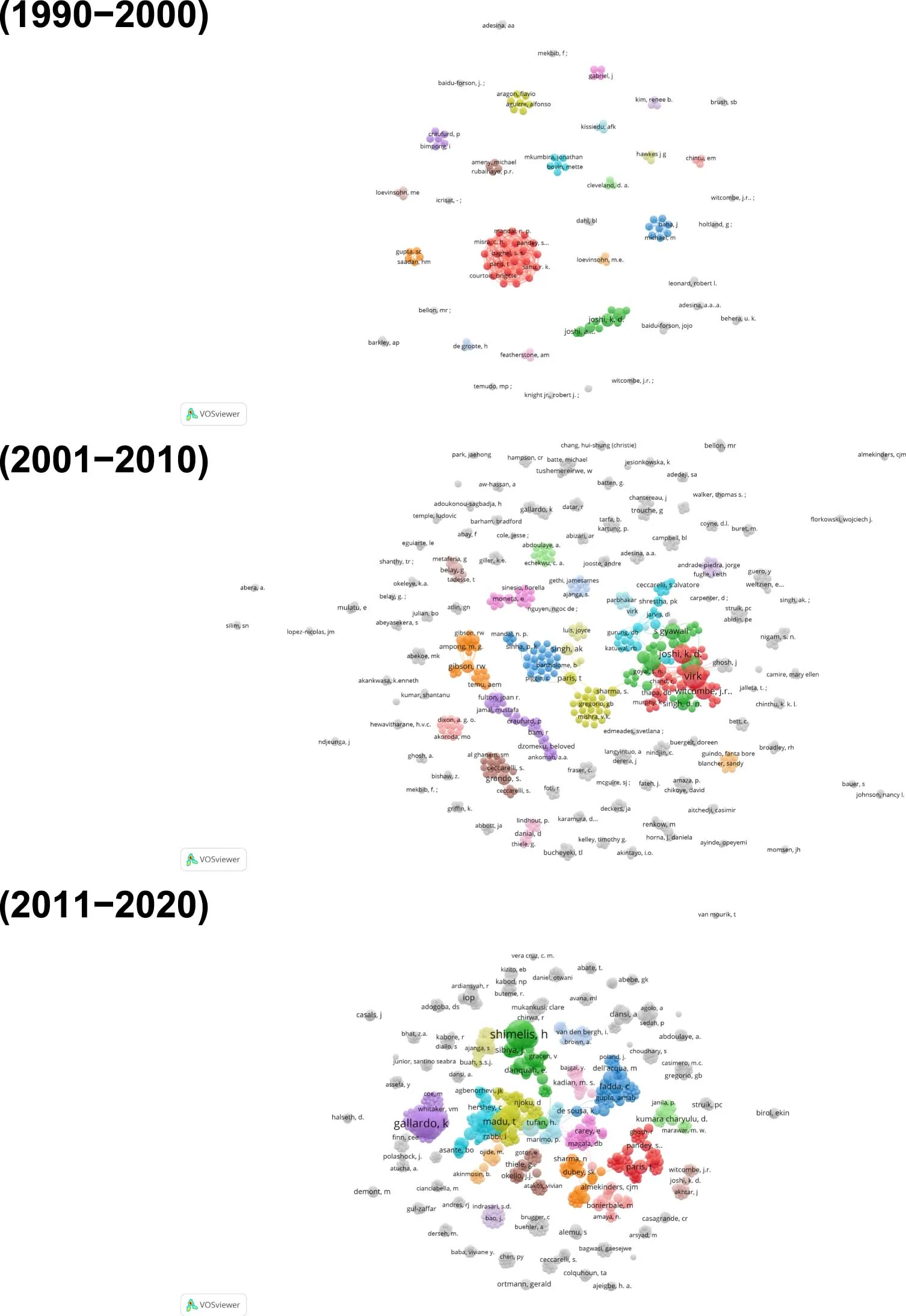
Network of co-authors, across time. The size of the nodes indicate higher number of collaborations. Edges indicate co-authorship. Nodes are grouped into clusters, which are distinguished by colors.

**Extended Data Fig. 5:**
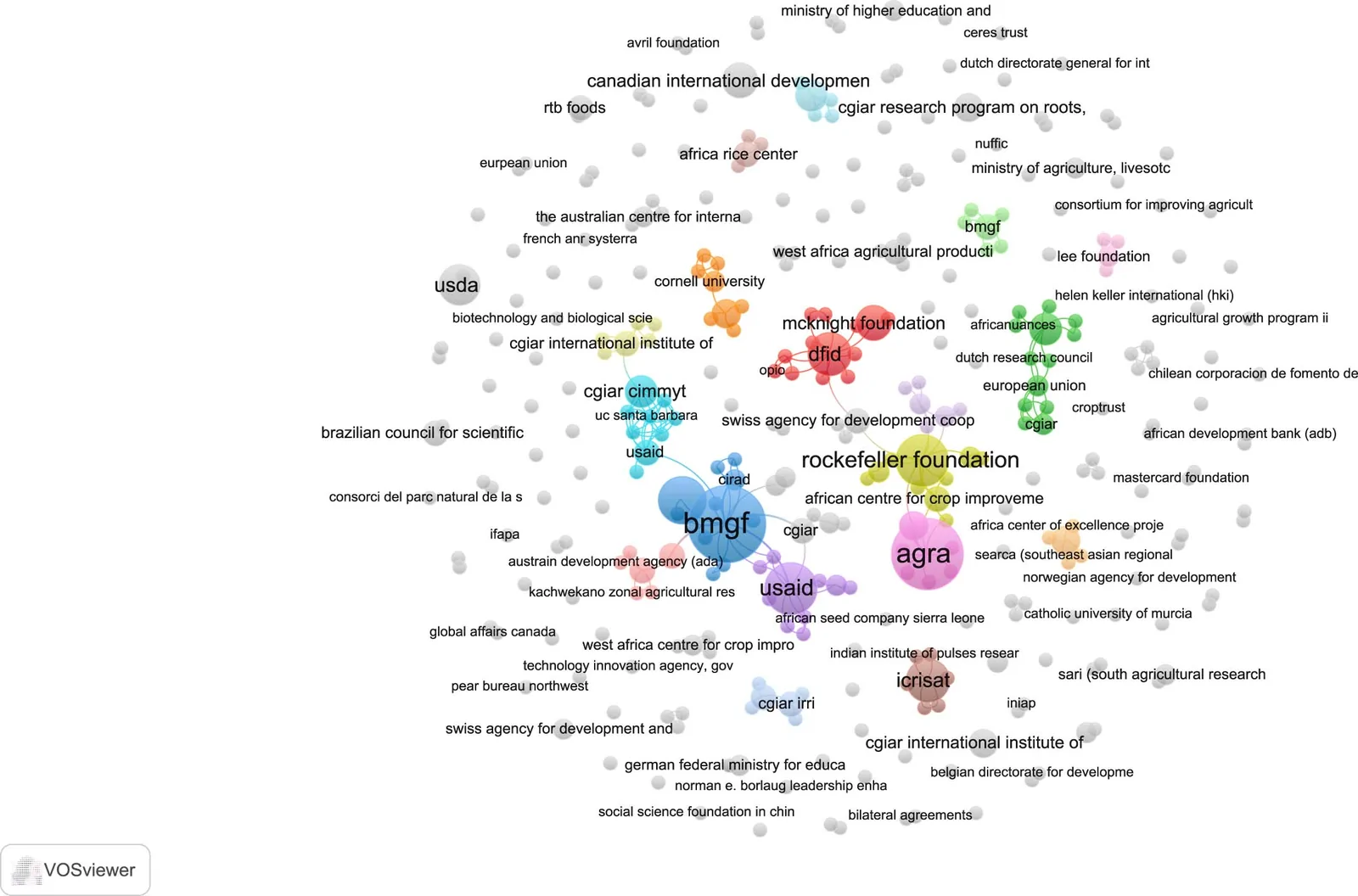
Network of donors. The size of the nodes indicate higher number of collaborations. Edges indicate co-authorship. Nodes are grouped into clusters, which are distinguished by colors.

Finally, we tracked the evolution of author networks across time to visualize the research and donor relationships in these studies. Figure 5a shows the co-authorship network where we observe 10 predominant clusters, each one characterized by the presence of one main author catalysing the collaboration. Collaborations between clusters are rare (Fig. 5b): despite being prolific in terms of studies, these co-authoring communities are isolated, and studies rarely involve authors from different clusters. The evolution through time is shown in Extended Data Fig. 4. Looking at the evolution of the authors’ network between 1990 and 2020, we find that the pattern in Fig. 5 is the result of recent years. The network of donors is reported in Extended Data Fig. 5. Funding for trait prioritization studies is highly centralized, with five donors accounting for nearly half of the studies in this scoping review.

## Discussion

Crops critically important for food security and nutrition, such as roots, tubers and bananas, or fruits and vegetables, receive less attention than cereals in the trait prioritization literature. Prioritization of cereals in national policies reflects an emphasis on calories as the primary driver of food security, which is frequently paired with a general lack of attention to nutrition sensitivity within agricultural programmes^17–20^ on crops that offer dietary diversity, such as fruits and vegetables focused on serving populations in Asia, sub-Saharan Africa or South America. This has consequences for vulnerable populations at risk of malnutrition and micronutrient deficiencies, especially during food crises. Recent findings claim that the effects of food inflation and recent food shortages increase wastage, increase the risk of stunting and decrease diet quality for 1.2 million children in 44 low and middle-income countries^21^. A lack of research attention on crop trait preferences for nutritious crops in this context risks perpetuating this systematic food and nutrition insecurity.

The lack of robustness in study design and taxonomy of traits limits the usefulness of trait prioritization data for crop breeding. It was nearly impossible to aggregate trait preferences across studies without a standardized taxonomy of traits, to enable data-driven decisions at scale. Taxonomies can have far-reaching impact beyond the plant science communities. Initiatives such as the recently announced ‘Vision for Adapted Crops and Soils’ demonstrate governments’ and international agencies’ response to identify the role of crop breeding to support agriculture’s response to climate change, especially for countries with the poorest and hungriest populations^22^. Without better methods guiding the data collection to support research, development, investment and prioritization decisions, crop breeding will not be able to deliver on its promise to support the world with healthy, nutritious and sustainable crops.

There is very little research on trait prioritization being conducted in South America despite a rising interest in crop breeding in the region^23^. This regional bias may reflect a choice to exclude non-English language materials. However, similar results are reported in other recent systematic scoping reviews in agriculture where Spanish-language publications were included^24,25^. This gap is also at odds with the centrality of participatory trait preference research for large public-sector crop breeding programmes in the region, such as the International Potato Center^26^. A lack of available robust research from this part of the world may have disastrous consequences, given climate change and South America’s importance to the global commodities trade^27,28^. Furthermore, a glaring dearth of data from the Middle East and North Africa raises questions around research neglect in this region at a time when widespread conflict and migration would require the opposite. Whether the regional bias in trait prioritization studies is driven by policy and donor priorities requires further analysis. However, the network mapping offers a glimpse into differences in donor priorities for groups of crops, as well as changes in the roles of different types of donor (universities vs foundations) over time. This raises questions on the types of study supported and how these replicate dominant donor priorities^29^.

Studies with robust designs, where clear research questions are accompanied by good data collection, methodological and analytical plans, are rare^30^. For example, robust sample size calculation is critical, with easily available strategies to do so (see ref. ^31^). Few papers discussed respondent selection and sample construction, raising questions on representativeness and external validity of the results they present. Sample size calculations were often overlooked by the authors and may indicate sampling guided by habit and practicality, rather than robust study design. While in some cases sample size calculations might simply not have been reported in studies even if conducted, we posit that a lack of attention to respondent sampling in trait prioritization studies could be producing misleading and unrepresentative results for crop breeders.

Understanding how and why gender shapes trait preferences is critical for breeding programmes to develop new varieties inclusively and equitably^32,33^. Gender analysis minimally requires sex-disaggregated trait prioritization data. Without this data, breeders remain uninformed on trait preferences important to women and may overgeneralize for communities on the basis of incomplete trait preference input^7,14^. In addition, recent literature documents how intersecting social identities and roles in crop value chains shape trait preferences^7,14,15,34,35^. Most studies do not report socio-economic or sex-disaggregated data on trait prioritization study respondents, although studies that do are more recently published studies (see refs. ^36,37^), potentially pointing to a shift in the practice in trait prioritization studies.

Robust study design includes appropriate data collection tools and analysis methods. Direct question-based user preference elicitation is most common in the studies we review. This could be problematic, as direct elicitation of user preferences has been shown to produce questionable results^38^. Another problematic trend we observe is the frequent use of qualitative methods (such as focus group discussions) in data collection, with very rare use of qualitative methods of analysis. Skills to collect and analyse high-quality qualitative data are exceedingly rare in public-sector breeding programmes^39^, which may explain this observation. Some of the more commonly used tools and analysis methods may also stem from the siloed research networks our analysis uncovered. Leading academic researchers playing key roles in networks may cause such siloing^40^.

This scoping review captures a rich history of participatory plant breeding^12^ (for specific case studies, see refs. ^14,41–44^). The results show wide variability across participatory trait prioritization studies, due in part to authors conflating ‘participation’ with ‘participatory’, only engaging end-users in a consultative form. Formal-led and consultative participatory breeding is more common in public-sector breeding^44^. Our findings confirm this trend, with only a small number of studies reporting trait prioritization data from farmers testing varieties in their own fields and with their own management practices. Studies that use on-farm testing (direct experience)^38^ or more-novel video-based product concept testing methods^45^ to capture trait prioritization may partially circumvent biases that arise from direct questions.

## Conclusion

Our findings identify key gaps in the current trait prioritization literature of interest to crop breeders, donors and the broader plant science community. The many data gaps—ranging from geography to socio-demographic, to crops critical for nutrition—raise questions on inequalities in research development and prioritization. Breeding programmes will struggle to become demand-driven through evidence-based decisions if errant data collection practices are not addressed. We conclude with recommendations to inform future trait prioritization studies to address these identified gaps. First, the lack of trait data from nutritious crops in sub-Saharan Africa, Asia and South America, and a dearth of data from the Middle East and North Africa present clear priorities for more research. Second, crop breeding management databases and increasingly well-developed crop ontology definitions^46^ should be linked more closely to trait preference studies to enable higher cross-comparability. Third, all studies should at least collect data from men and women, and sex disaggregate data at collection and analysis to enable gender-informed trait prioritization. Lastly, rigorous study design, including representative sampling, power calculations, and choice of appropriate research methods and analysis approaches underpin the validity and utility of future trait prioritization studies.

## Methods

The reporting guidelines for a systematic scoping review, PRISMA-ScR, were used to examine the tools and methods for trait elicitation and ranking over the past 40 years in plant breeding programmes across time, study locations, crop groups, institutions and genders^47^. Systematic scoping reviews are used to explore the evidence in a field to address questions relating to what is known about a topic, what can be synthesized from existing studies to develop policy or practice recommendations, and what aspects of a topic have yet to be addressed by researchers^48^. Systematic scoping reviews are gaining popularity across agricultural development and other fields because they provide a birds-eye view of evidence gaps across a field of research and are useful for further in-depth research on a particular topic^49–51^.

**Fig. 6:**
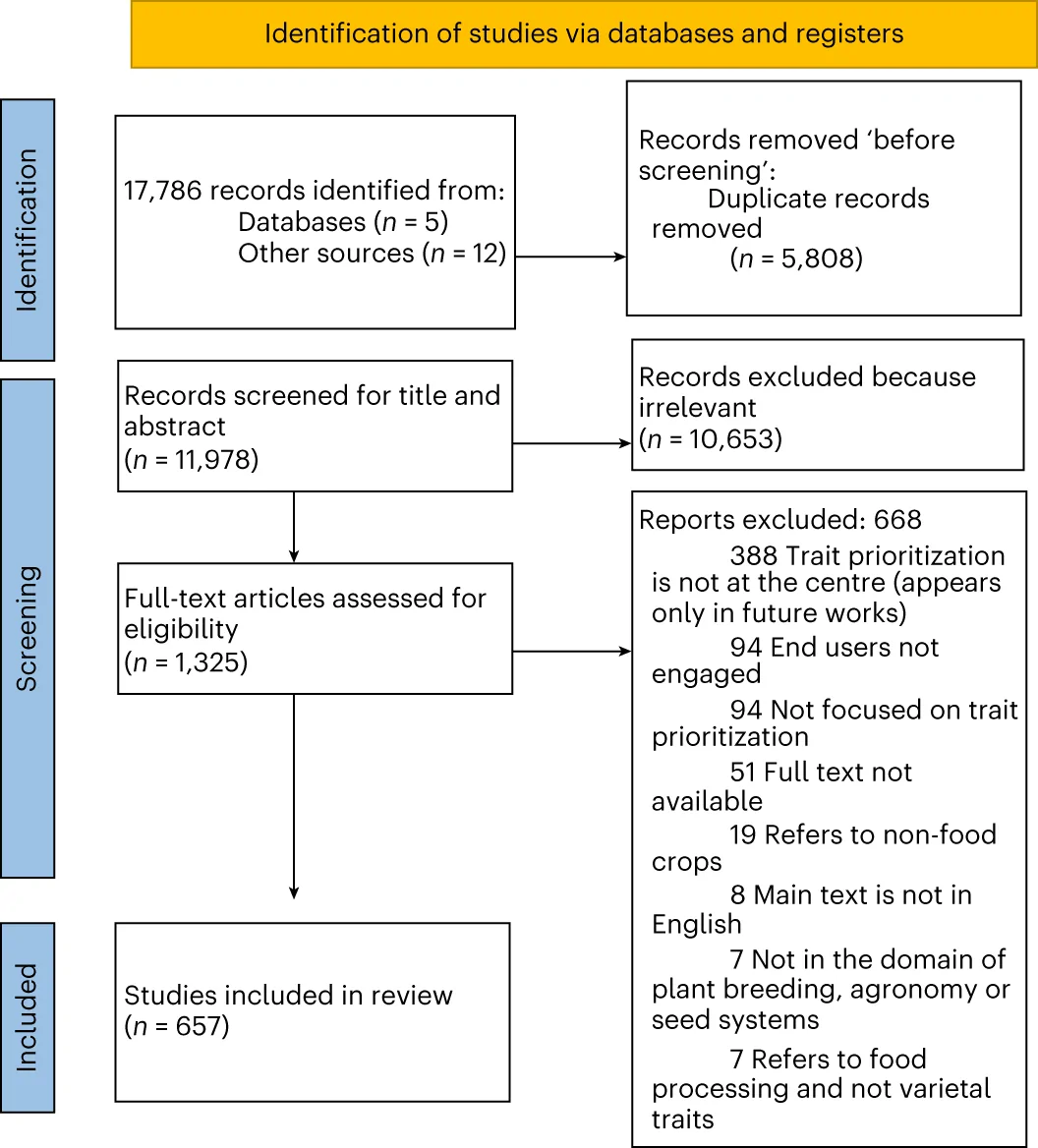
PRISMA flow diagram. Overview of the steps used in the process of screening, with number of records at each step.

Figure 6 presents the PRISMA flowchart used in the process of screening the literature for this scoping review. The Supplementary Information outlines the complete protocol used in this study.

### Protocol and registration

The protocol was created following PRISMA-ScR^47^ and registered on 11 March 2022. The approved protocol detailed exclusion and inclusion criteria, definitions, review questions and the search strategy. The protocol was updated and finalized on 8 July 2022 (https://osf.io/ayw8q). After input from reviewers, the search was broadened and updated, and a revised search was done on 23 June 2023.

### Research question and eligibility criteria

The guiding question for the scoping review is: What tools and methods have been used for trait prioritization in plant breeding programmes across time, study locations, crop groups, institutions and genders?

Inclusion consisted of original research or reviews that explicitly discuss trait prioritization as it relates to crops, varieties, seeds, planting materials or germplasm. Any articles that reported on research outside of the domains of plant breeding, seed systems, or agronomy (for example, livestock or non-food crop studies) were excluded. The search had no restriction on dates and geographical scope. Only studies published in English were included.

### Information sources

We searched five electronic databases of scholarly journal articles for a comprehensive retrieval: Scopus, Web of Science, CAB Direct, AgEcon Search and BIOSIS. A large body of relevant literature is likely to exist outside of scholarly publications, so 12 grey literature sources were searched as well: Commonwealth Scientific and Industrial Research Organization (CSIRO), Gardian, International Fund for Agricultural Development (IFAD), JPAL (Abdul Latif Jameel Poverty Action Lab) /ATAI (Agricultural Technology Adoption Initiative) impact evaluations (IPA), Overseas Development Institute (ODI), UK Department for International Development (DFID), World Bank, World Health Organization (WHO), United Nations Environment Programme (UNEP), World Food Programme (WFP), Food and Agriculture Organization (FAO) and AgriLinks (USAID Feed the Future platform). While there was substantial overlap between some of these grey literature sources, searching all sources was necessary to exhaust the literature^52^.

### Search strategy

As a search strategy, we used keywords and subject headings to discover articles that covered the population and topic identified in the research question. An example of a search strategy for Web of Science is included below.

**Table:** 

Row number	Search string
1	TS= (farme* OR household OR supplie* OR consume* OR producer OR trade* OR processo* OR dealer OR student* OR expert* OR seed company OR responde* OR stakeh* OR particip*)
2	TS= ((trait OR variet* OR characterist*) NEAR (prefer* OR priorit* OR select* OR adopt*))
3	TS= (((breed* AND (plant OR variet*)) OR crop* OR agronom* OR farm* OR agricultur* or seed*))
4	1 AND 2 AND 3

We investigated each source for optimal search strategy and translated the search into the most appropriate terminology for that source. The grey literature sources, most of which do not include advanced search features, were searched by manually inputting the keywords and selecting results that fit the inclusion criteria. The search strategies and all resources searched are provided in Supplementary Information.

### Selection of sources of evidence

We performed the final searches of all scholarly databases on 23 June 2023. Searches of all grey literature sources took place over the period from 12 March 2022 to 6 July 2022. Upon completion of all searches, 17,786 records were retrieved and uploaded into Covidence for review (https://www.covidence.org/). After deduplication, a total of 11,978 unique records was obtained. After screening based on titles and abstracts, 1,325 studies were identified as being potentially relevant to the research topic. We obtained the full-text versions of the articles, with each article being reviewed and confirmed as appropriate by the authors. After completing this process, 657 studies were included in the data extraction (Fig. 6).

### Data extraction

A peer-review process was used for title, abstract and full-text screening. A data-extraction framework of 40 questions supported the inclusion and selection process. Extensive pre-testing helped tailor the set of questions and asked a minimum number of information that could be answered by all papers. The framework is provided in Supplementary Information, with a full extraction template reported.

### Data synthesis

After the extraction phase, data were synthetized descriptively using R (v.4.2.3) and R studio^53^. The network representation of authors, affiliations and donors was carried out in VOSviewer^54^. We grouped data according to four major crop categories: cereals, legumes, RTB, and vegetables and fruits. Commodity crops (spices, cotton, coffee and sugarcane) and ornamental plants composed 3% of the sample and were excluded from the data synthesis. Table 1 summarizes the variables used in data synthesis to answer the main research question of the study.

**Table 1:** Summary of main variables analysed in the study

Variable type	Information collected
Trait prioritization taxa	Mention of trait prioritization vs mention of trait preferences
Journal/Publishing organization	Journal/Organization publishing the study
Institution	Institution that implemented the trait prioritization study
Donor	Donor providing funding for the trait prioritization study
Crop	Specific crop at the centre of the study
Variety	Varieties of the crop considered
Ranking	If the traits were ranked
Data disaggregation	Trait ranking data disaggregated by any variable (sex, region, age or any other variable specified)
Timeframe	Year(s) in which the trait prioritization study was conducted
Declared breeding purpose	Any statements on the aims of the breeding programme, for yield vs for climate vs for nutrition vs for food security
Geographic area of the study	Individual country vs regional vs global
Data analysis method	Specific method used to analyse the trait preferences collected. We categorize methods according to the type of analysis performed. We distinguish between ranking methods, qualitative methods, descriptive methods, statistical hypothesis testing and econometric methods. We heuristically structure methods according to their main analytical purpose.
Data collection tool	Specific tool used to collect trait preferences. We categorize tools according to the type of trait prioritization performed. We distinguish among preferences elicited through direct questions, choice experiments, direct experience or models and secondary data. We categorize the tools as such to reduce the complexity of the data extracted (a total of 21 unique tools were mentioned across studies), choosing to heuristically structure the tools around these themes.
Specific mention of	Local vs traditional vs indigenous knowledge
Specific mention of	Priority setting
Degree of end-users’ engagement	To analyse the data on farmers’ engagement, we relied on the framework of ref. ^23^. The framework distinguishes between formal-led and farmer-led participatory breeding research. The institutional approach suggested by ref. ^23^ identifies two loci of control or decision making and two main institutional approaches: one where farmers join in breeding experiments that have been initiated by formal breeding programmes (formal-led participatory breeding) and another where scientists seek to support farmers’ own systems of breeding, varietal selection and seed maintenance (farmer-led participatory breeding). We adopt this framework in investigating end-users’ engagement in ‘Trait prioritization studies are mainly formally led’.

Unless otherwise specified, in categorizing methods and tools, we allowed papers with multiple tools and methods to be included in multiple categories. This might have led to double counting on some occasions.

### Reporting summary

Further information on research design is available in the Nature Portfolio Reporting Summary linked to this article.

## Supplementary information

Supplementary Information Supplementary Tables 1–4, a list of all included studies and the study protocol.

Reporting Summary

## Data Availability

The data utilized for this study are publicly available and are hosted on the GitHub repository at https://github.com/TufanLab/trait-priority-scoping-review.git.

## Code availability

The code utilized for this study is publicly available and is hosted on the GitHub repository at https://github.com/TufanLab/trait-priority-scoping-review.git.
