# Supplementary material for: A scoping review on tools and methods for trait prioritization in crop breeding programmes
Source: Nat Plants. 2024 Feb 22;10(3):402–11. doi: 10.1038/s41477-024-01639-6 (PMC10954539; doi:10.1038/s41477-024-01639-6)
Supplement: Supplementary file 1 — Supplementary Tables 1–4, a list of all included studies and the study protocol. [file 41477_2024_1639_MOESM1_ESM.pdf]

nature plants

Article https://doi.org/10.1038/s41477-024-01639-6

A scoping review on tools and methods for trait prioritization in crop breeding programmes

In the format provided by the authors and unedited

Supplementary information Occelli et al

Table S1 | Frequencies of tools in the studies sampled, sorted in descending order

Broad Tool Type	Narrow Tool Type	Frequency
Questions	Individual surveys	33.6
Questions	FGDs	14.1
Experience	PVS	10
Experience	Demonstration plots	5.2
Other	Sensory test	6.1
Questions	Preference voting	5.9
Experience	Field visit	4.09
Experience	On farm trials	3.2
Choices	WTP	3
Other	Models and maps	2.9
Choices	Choice experiment	2.9
Questions	Group surveys	1.96
Other	Observation of farmers behavior	1.4
Experience	On station trial	1.2
Questions	PRA	1.2
Choices	Auction, Hedonic pricing	1.1
Choices	Gamification	0.3
Experience	Other	0.3
Questions	Tricot	0.3
Questions	Expert surveys	0.5
Questions	G+ Tools	0.1
Total		100

Table S2 | Frequencies of methods in the studies sampled, sorted in descending order

Broad Tool Type	Narrow Tool Type	Frequency
Descriptive and frequency	Frequency count	26.1
Statistical Hyp. Testing	ANOVA	13.8
Statistical Hyp. Testing	T-test between rankings	13.7
Descriptive and frequency	Descriptive Analysis	13.7
Economic model	GLM	9.9
Ranking	Ranking	7.6
Statistical Hyp. Testing	Chi-squared test	5.8
Multivariate analysis	Other	5.5
Economic model	Other	5
Multivariate analysis	PCA	4.3
Ranking	Pair wise Ranking	3.3
Statistical Hyp. Testing	LSD	3.6
Multivariate analysis	Cluster, factor analysis	3
Qualitative	Quote statement	1.9
Qualitative	Content analysis	1.7
Index	Index	1.7
Qualitative	Other	1.4
Qualitative	Word counting	1.1
Descriptive and frequency	Tabular analysis	1.1
Multivariate analysis	Matrix scoring	0.8
Descriptive and frequency	Figures	0.6
Descriptive and frequency	Direct prioritization	0.3
Ranking	Priority ranking	0.3
Statistical Hyp. Testing	Other	0.1
Total		100

Table S3 | Raw characteristics listed in a sub-sample of studies, according to tools and methods used

Data collection year	PP	Sex	Tools	Method	Authors (lead and last)	Open- or close-ended questions	Raw list of characteristics
2007	Y	N	Experience; Other (sensory)	Descriptive; Multivariate Analysis	Singh Y.P; Ismail A.M.	Open	Number of productive tillers, dwarf plant stature, early maturity, attractive long slender grains, better threshability.
2006	Y	N	Experience; Questions	Descriptive; Ranking	R.K. Singh; N. T. Lang (Singh Y.P; Ismail A.M. are co-authors in the study)	Open	Yield, ability to withstand salt stress, flowering duration, plant height, quality of straw for fodder, for use as building material, taste, cooking quality, bold grain shape, good volume-expansion ratio of cooked rice.
2008	N	N	Questions	Descriptive; Economic model	Malabayabas M.; Pandey S.	Closed	High yield, resistance to lodge, resistance to pests, resistance to drought, good for submergence, good taste, good quality of grains, high price, high demand.

The sub-sample of studies is selected among the highest number of studies on cereals in the period 2005 – 2010. Within this time span, we extract the crop appearing in most studies and within the selected target crop, we choose the country with more studies implemented. All the three studies are focused on rice in India. They do not collect sex-disaggregated data (column Sex in the table) and authors do not mention the methodology as being participatory (column PP in the table).

Table S4 | Top-three ranked traits for three specific crops in each crop group, by decade

	1990 - 1999	2000 - 2009	2010 - 2020
**Cereals**			
Rice	1. High yield<br>2. Easy of threshing<br>3. Early maturity	1. High yield<br>2. Tillering capacity<br>3. Early maturity	1. High yield<br>2. Pest disease resistance<br>3. Early maturity
Maize	1. High yield<br>2. Organoleptic characteristics<br>3. Early maturity	1. Insect resistance<br>2. Storability<br>3. High yield	1. High yield<br>2. Drought tolerance<br>3. Early maturity
Sorghum	1. High yield<br>2. Vigor<br>3. Early maturity	1. High yield<br>2. Grain quality	1. High yield<br>2. Taste<br>3. Early maturity
**Legumes**			
Bean	1. High yield<br>2. market demand<br>3. performs well under bananas	1. High yield<br>2. Early maturity<br>3. Taste	1. Pest disease resistance<br>2. Taste<br>3. Plant architecture
Cowpea	-	1. Market demand<br>2. Early maturity<br>3. High yield	1. Straight pod shape<br>2. A long pod size bearing at least 10 seeds
Soybean	-	1. Early maturity<br>2. Number of pods<br>3. Filling of pods	-
**RTB crops**			
Potato	1. Flavor<br>2. Dry matter<br>3. Flour content	1. Skin quality<br>2. Size<br>3. Color	1. High yield<br>2. Tuber size<br>3. Tuber number
Banana	-	1. Texture<br>2. Taste<br>3. Color	1. Taste<br>2. Finger size<br>3. Number of fingers
Cassava	-	1. High yield<br>2. Dry matter content<br>3. Taste	1. High yield<br>2. Pest disease resistance<br>3. Plant height
**Vegetable and Fruits**			

Apple	1. Low price<br>2. Color<br>3. Large size	1. Flavor<br>2. Juiciness<br>3. Sweetness	1. Crispiness<br>2. Size<br>3. Sweetness
Tomato	-	1. Color<br>2. Texture<br>3. Size and shape	1. Flavor<br>2. Color<br>3. Resistance to physiological disorders
Chili	-	1. Uniformity<br>2. Pest disease resistance<br>3. Pest disease resistance	-

<u>List of all included studies</u>

1. Fritz, K. *et al.* Flavor quality and composition of accession resources in the North Carolina State University peanut breeding program. *CROP SCIENCE* 62, 1880–1890 (2022).
2. Ndjouenkeu, R. *et al.* From cassava to gari: mapping of quality characteristics and end-user preferences in Cameroon and Nigeria. *Int J Food Sci Technol* 56, 1223–1238 (2021).
3. Balogun, I. *et al.* From traits to typologies: Piloting new approaches to profiling trait preferences along the cassava value chain in Nigeria. *CROP SCIENCE* 62, 259–274 (2022).
4. Campbell, B. *et al.* Fruit quality characteristics that affect consumer preferences for satsuma mandarins. *HORTSCIENCE* 39, 1664–1669 (2004).
5. Tsusaka, T., Orr, A., Msere, H., Harris, D. & Rao, N. V. G. Fuelwood or Grain? A Conjoint Analysis of Trait Preferences for Pigeonpea among Smallholders in Southern Malawi. (2018) doi:10.22004/ag.econ.277435.
6. Steinke, J. & van Etten, J. Gamification of farmer-participatory priority setting in plant breeding: Design and validation of "AgroDuos". *Journal of Crop Improvement* 31, 356–378 (2017).
7. Oakley, E. & Momsen, J. Gender and agrobiodiversity: a case study from Bangladesh. *GEOGRAPHICAL JOURNAL* 171, 195–208 (2005).
8. Weltzien, E., Rattunde, F., Christinck, A., Isaacs, K. & Ashby, J. Gender and farmer preferences for varietal traits: Evidence and issues for crop improvement. in *Plant Breeding Reviews* 243–278 (2019). doi:10.1002/9781119616801.ch7.
9. Ayinde, O. E., Abdoulaye, T., Olaoye, G. & Akangbe, J. Gender and innovation in agriculture: a case study of farmers varietal preference of drought tolerant maize in southern Guinea Savannah region of Nigeria. *Albanian Journal of Agricultural Sciences* (2013).
10. Marimo, P. *et al.* Gender and Trait Preferences for Banana Cultivation and Use in Sub-Saharan Africa: A Literature Review(1). *Economic Botany* 74, (2020).
11. Addison, M., Mujawamariya, G. & Barn, R. Gender considerations in development and utilisation of technological innovations: evidence from Ghana. *DEVELOPMENT IN PRACTICE* 30, 15–26 (2020).
12. Feyisa, T. G. & Megersa, H. G. Gender differences in fruit production and selection of avocado and banana varieties: Case study at wondo genet and wondo woredas, Southern Ethiopia. *International Journal of Agricultural Extension* 8, 77–83 (2020).
13. Njuguna-Mungai, E. *et al.* Gender dynamics around introduction of improved forages in Kenya and Ethiopia. *Agronomy Journal* 114, 277–295 (2022).
14. Voss, R. C., Donovan, J., Rutsaert, P. & Cairns, J. E. Gender inclusivity through maize breeding in Africa: A review of the issues and options for future engagement. *Outlook on Agriculture* 50, 392–405 (2021).
15. Elango, D. & Kawarazuka, N. *Gender responsive participatory varietal selection for sustainable seed potato systems in Assam, India.* https://cgspace.cgiar.org/handle/10568/102487 (2019).
16. Stevens, M., Lea-Cox, J., Black, B. & Abbott, J. A comparison of fruit quality and consumer preferences among three cold-climate strawberry production systems. *HORTTECHNOLOGY* 17, 586–591 (2007).
17. Varshney, R. K., Ojiewo, C. & Monyo, E. A decade of Tropical Legumes projects: Development and adoption of improved varieties, creation of market-demand to benefit smallholder farmers and empowerment of national programmes in sub-Saharan Africa and South Asia. *Plant Breeding* 138, 379–388 (2019).

18. Gebretsadik, R., Shimelis, H., Laing, M., Tongoona, P. & Mandefro, N. A diagnostic appraisal of the sorghum farming system and breeding priorities in Striga infested agro-ecologies of Ethiopia. *AGRICULTURAL SYSTEMS* 123, 54–61 (2014).
19. Mujawamariya, G., Zenna, N., Leonard, L., Andrianina, I. & Ramiaramanana, D. A gendered outlook into the adoption of improved rice varieties in Madagascar. *GENDER TECHNOLOGY & DEVELOPMENT* 26, 1–27 (2022).
20. Edmeades, S. A hedonic approach to estimating the supply of variety attributes of a subsistence crop. 49 (2006) doi:10.22004/ag.econ.55424.
21. Dalton, T. A household hedonic model of rice traits: economic values from farmers in West Africa. *AGRICULTURAL ECONOMICS* 31, 149–159 (2004).
22. Kikulwe, E., Birol, E., Wesseler, J. & Falck-Zepeda, J. A latent class approach to investigating demand for genetically modified banana in Uganda. *Agricultural Economics* 42, 547–560 (2011).
23. Ceccarelli, S. *et al.* A methodological study on participatory barley breeding - I. Selection phase. *EUPHYTICA* 111, 91–104 (2000).
24. Ceccarelli, S. *et al.* A methodological study on participatory barley breeding II. Response to selection. *EUPHYTICA* 133, 185–200 (2003).
25. A participatory approach for landrace evaluation. http://www.bioversityinternational.org/e-library/publications/detail/a-participatory-approach-for-landrace-evaluation/.
26. Kamara, A. Y. *et al.* A participatory evaluation of improved cowpea cultivars in the Guinea and Sudan savanna zones of north east Nigeria. *Archives of Agronomy and Soil Science* 56, 355–370 (2010).
27. Valle, J. F. *A review of crop variety evaluation in Roots, Tubers and Bananas: geographic coverage, approaches, trait inclusion, and gender aspects*. https://cgspace.cgiar.org/handle/10568/111047 (2021).
28. Greenberg, S. *A review of participatory plant breeding and lessons for African seed and food sovereignty movements (ACB report)*. (2018).
29. Thiele, G. *et al.* A review of varietal change in roots, tubers and bananas: consumer preferences and other drivers of adoption and implications for breeding. *International Journal of Food Science and Technology* 56, 1076–1092 (2021).
30. Acevedo, M. *et al.* A scoping review of adoption of climate-resilient crops by small-scale producers in low- and middle-income countries. *Nature Plants* 6, 1231–1241 (2020).
31. Ghosh, A., Satya, P. & Mukhopadhyay, P. A simple quantitative method to judge the need of participatory plant breeding programme. *Indian Journal of Genetics & Plant Breeding* 67, 374–380 (2007).
32. Quaye, W. *et al.* A socio-economic assessment of cowpea diversity on the Ghanaian market: implications for breeding. *INTERNATIONAL JOURNAL OF CONSUMER STUDIES* 35, 679–687 (2011).
33. Vijayachandra Reddy, S. & Kammar, S. A study on preferred quality traits in marketing and consumption of Pigeon pea in Karnataka State. *Ecology, Environment and Conservation* 22, S411–S414 (2016).
34. Rocha, M., Deliza, R., Correa, F., do Carmo, M. & Abboud, A. A study to guide breeding of new cultivars of organic cherry tomato following a consumer-driven approach. *FOOD RESEARCH INTERNATIONAL* 51, 265–273 (2013).
35. Useche, P., Barham, B. & Foltz, J. A Trait Specific Model of GM Crop Adoption by Minnesota and Wisconsin Corn Farmers. 37 (2006) doi:10.22004/ag.econ.201525.
36. Joshi, K. *et al.* Acceptance and competitiveness of new improved wheat varieties by

smallholder farmers. *JOURNAL OF CROP IMPROVEMENT* 31, 608–627 (2017).
37. Hussen, M. & Biru, T. Adaptation and Participatory Evaluation of Improved Sorghum (Sorghum bicolor (L.) Moenech) Varieties in Southwestern Ethiopia. *Russian Agricultural Sciences* 47, S77–S89 (2021).
38. Alam, M. S., Islam, M. A., Paris, T. & Cueno, A. Adaptation Strategies and Coping Mechanism Against Climate Change in Coastal Bangladesh. 30 (2011) doi:10.22004/ag.econ.290547.
39. Tegbaru, A. *et al.* Addressing gendered varietal and trait preferences in West African maize. *World Development Perspectives* 20, (2020).
40. Lawal, B. O., Saka, J. O., Oyegbami, A. & Akintayo, I. O. Adoption and performance assessment of improved maize varieties among smallholder farmers in southwest nigeria. *Journal of Agricultural and Food Information* 6, 35–47 (2004).
41. Sarkar, M. *et al.* Adoption Determinants of Exotic Rice Cultivars in Bangladesh. *FRONTIERS IN SUSTAINABLE FOOD SYSTEMS* 6, (2022).
42. Adeola, R. G., Ogunleye, K. Y. & Adewole, W. A. Adoption Intensity Determinants for Improved Sweet Potato Varieties among Farmers in Nigeria. *International Journal of Agricultural Management and Development (IJAMAD)* (2019) doi:10.22004/ag.econ.292783.
43. Agwu, A. & Anyaeche, C. Adoption of improved cassava varieties in six rural communities in Anambra State, Nigeria. *AFRICAN JOURNAL OF BIOTECHNOLOGY* 6, 89–98 (2007).
44. Abebe, G., Bijman, J., Pascucci, S. & Omta, O. Adoption of improved potato varieties in Ethiopia: The role of agricultural knowledge and innovation system and smallholder farmers' quality assessment. *AGRICULTURAL SYSTEMS* 122, 22–32 (2013).
45. Pradel, W., Gatto, M., Hareau, G., Pandey, S. & Bhardway, V. Adoption of potato varieties and their role for climate change adaptation in India. *CLIMATE RISK MANAGEMENT* 23, 114–123 (2019).
46. Wang, Z. *et al.* Adoption of table grape cultivars: An attribute preference study on Chinese grape growers. *SCIENTIA HORTICULTURAE* 216, 66–75 (2017).
47. Felix, A., Roferos, L., Bandonill, E. & Julian, B. Amylose content preference for cooked milled rice among Filipino consumers in non-irrigated lowland ecosystems. *PHILIPPINE AGRICULTURAL SCIENTIST* 90, 206–214 (2007).
48. JACKSON M T, HAWKES J G, & ROWE P R. AN ETHNOBOTANICAL FIELD STUDY OF PRIMITIVE POTATO VARIETIES IN PERU. *Euphytica* 29, 107–114 (1980).
49. Yue, C. *et al.* An Evaluation of US Peach Producers' Trait Prioritization: Evidence from Audience Surveys. *Hortscience* 49, 1309–1314 (2014).
50. Yue, C. *et al.* An Evaluation of US Strawberry Producers Trait Prioritization: Evidence from Audience Surveys. *HORTSCIENCE* 49, 188–193 (2014).
51. Yue, C. *et al.* An Evaluation of US Tart and Sweet Cherry Producers Trait Prioritization: Evidence from Audience Surveys. *Hortscience* 49, 931–937 (2014).
52. Forsythe, L., Tufan, H., Bouniol, A., Kleih, U. & Fliedel, G. An interdisciplinary and participatory methodology to improve user acceptability of root, tuber and banana varieties. *International Journal of Food Science & Technology* 56, 1115–1123 (2021).
53. Gallardo, R. *et al.* An Investigation of Trait Prioritization in Rosaceous Fruit Breeding Programs. *HORTSCIENCE* 47, 771–776 (2012).
54. Yue, C. *et al.* An Investigation of Trait Prioritizationin Turfgrass Breeding Programs. *HORTSCIENCE* 52, 1544–1549 (2017).
55. Yue, C. *et al.* An Investigation of US Apple Producers' Trait Prioritization-Evidence from Audience Surveys. *HORTSCIENCE* 48, 1378–1384 (2013).

56. DeYoung, D. J. *et al.* An Overview of Bean Production Practices, Varietal Preferences, and Consumption Patterns in the Milpa System of the Guatemalan Highlands: Results of a Farm Household Survey. 69 (2017) doi:10.22004/ag.econ.268951.
57. Corollaro, M., Gasperi, F. & Grappadelli, L. An Overview of Sensory Quality of Apple Fruit. *JOURNAL OF THE AMERICAN POMOLOGICAL SOCIETY* 68, 141–157 (2014).
58. IOP *et al.* Analysis of sensory characteristic rice of Padi Gogo (dry land paddy) at Aceh Province. in vol. 425 (2020).
59. Chentoufi, L. *et al.* Anchoring durum wheat diversity in the reality of traditional agricultural systems: varieties, seed management, and farmers' perception in two Moroccan regions. *JOURNAL OF ETHNOBIOLOGY AND ETHNOMEDICINE* 10, (2014).
60. Serrano-Megias, M. & Lopez-Nicolas, J. Application of agglomerative hierarchical clustering to identify consumer tomato preferences: influence of physicochemical and sensory characteristics on consumer response. *JOURNAL OF THE SCIENCE OF FOOD AND AGRICULTURE* 86, 493–499 (2006).
61. Mohammed, S. B. *et al.* Appraisal of cowpea cropping systems and farmers' perceptions of production constraints and preferences in the dry savannah areas of Nigeria. *CABI Agriculture and Bioscience* 2, 25 (2021).
62. Mogga, M., Sibiya, J., Shimelis, H., Lamo, J. & Ochanda, N. APPRAISAL OF MAJOR DETERMINANTS OF RICE PRODUCTION AND FARMERS' CHOICE OF RICE ID EOTYPES IN SOUTH SUDAN: IMPLICATIONS FOR BREEDING AND POLICY INTERVENTIONS. *EXPERIMENTAL AGRICULTURE* 55, 143–156 (2019).
63. Teklu, D. H., Shimelis, H., Tesfaye, A. & Abady, S. Appraisal of the sesame production opportunities and constraints, and farmer-preferred varieties and traits, in Eastern and Southwestern Ethiopia. *Sustainability (Switzerland)* 13, (2021).
64. Teferi, E., Kassie, G., Pe, M. & Fadda, C. Are farmers willing to pay for climate related traits of wheat? Evidence from rural parts of Ethiopia. *AGRICULTURAL SYSTEMS* 185, (2020).
65. Mwiti, F., Okello, J. J. & Munei, K. Are Farmers Willing to Pay for Quality Planting Materials of Clonally Propagated Biofortified Crops? The Case of Orange-Fleshed Sweetpotatoe in Tanzania. 24 (2015) doi:10.22004/ag.econ.212519.
66. Bosch, C., Zeller, M. & Deffner, D. Are seed distributions effective? Evidence from a randomly controlled experiment with improved bean seeds in rural Madagascar. 26 (2016) doi:10.22004/ag.econ.249286.
67. Gallardo, R. K., Yue, C., McCracken, V., Luby, J. & McFerson, J. Are WTP Estimates for Fruit Quality Similar between Growers and Consumers? Results of a Choice Experiment on Four Rosaceous Fruit Crops. 27 (2016) doi:10.22004/ag.econ.235548.
68. McElhinny, E. *et al.* Aspects of participatory plant breeding for quinoa in marginal areas of Ecuador. *Euphytica* 153, 373–384 (2007).
69. Zander, K., Blaise, P. & Holm-Muller, K. Assessing German farmers' trade-offs between disease resistance and yield in winter wheat varieties. *OUTLOOK ON AGRICULTURE* 52, 67–78 (2023).
70. Larochelle, C., Asare-Marafo, D., Birol, E. & Alwang, J. Assessing the Adoption of Improved Bean Varieties in Rwanda and the Role of Varietal Attributes in Adoption Decisions. (2016).
71. Paris, T., Singh, A., Cueno, A. & Singh, V. Assessing the impact of participatory research in rice breeding on women farmers: A case study in eastern Uttar Pradesh, India. *EXPERIMENTAL AGRICULTURE* 44, 97–112 (2008).
72. Agre, A. *et al.* Assessment of cassava (Manihot esculenta Crantz) diversity, loss of

landraces and farmers preference criteria in southern Benin using farmers' participatory approach. *GENETIC RESOURCES AND CROP EVOLUTION* 64, 307–320 (2017).
73. Nanyonjo, A. *et al.* Assessment of end user traits and physicochemical qualities of cassava flour: a case of Zombo district, Uganda. *INTERNATIONAL JOURNAL OF FOOD SCIENCE AND TECHNOLOGY* 56, 1289–1297 (2021).
74. Mengistu, G., Shimelis, H., Laing, M. & Lule, D. Assessment of farmers' perceptions of production constraints, and their trait preferences of sorghum in western Ethiopia: implications for anthracnose resistance breeding. *Acta Agriculturae Scandinavica Section B: Soil and Plant Science* 69, 241–249 (2019).
75. Nkhata, W. *et al.* Assessment of smallholder farmers' awareness of bean fly (Ophiomyia spp.) and management practices in central and northern Malawi: Implications for resistance breeding. *CROP PROTECTION* 139, (2021).
76. Andiku, C. *et al.* Assessment of sorghum production constraints and farmer preferences for sorghum variety in Uganda: implications for nutritional quality breeding. *Acta Agriculturae Scandinavica Section B: Soil and Plant Science* 71, 620–632 (2021).
77. Adejuwon, J. O. Assessment of the Changing Pattern in Maize Cultivation in Sokoto-Rima River Basin, Nigeria. *Journal of Applied Sciences & Environmental Management* 22, 1433–1437 (2018).
78. Shumbusha, D., Shimelis, H., Laing, M. & Rukundo, P. Assessment of the roles and farmer-preferred traits of sweetpotato in a crop-livestock farming system in Rwanda: Implications for breeding dual-purpose varieties. *Open Agriculture* 5, 834–843 (2020).
79. Kagoda, F., Derera, J., Tongoona, P. & Coyne, D. L. Awareness of plant-parasitic nematodes, and preferred maize varieties, among smallholder farmers in east and southern Uganda: Implications for assessing nematode resistance breeding needs in African maize. *International Journal of Pest Management* 56, 217–222 (2010).
80. B, E. *et al.* Banana and plantain (Musa spp.) cultivar preference, local processing techniques and consumption patterns in Eastern Democratic Republic of Congo. *International Journal of Agriculture Sciences* (2012).
81. Gibson, R. W., Mpembe, I. & Mwanga, R. O. M. Benefits of participatory plant breeding (PPB) as exemplified by the first-ever officially released PPB-bred sweet potato cultivar. *The Journal of Agricultural Science* 149, 625–632 (2011).
82. Teeken, B. *et al.* Beyond 'Women's Traits': Exploring How Gender, Social Difference, and Household Characteristics Influence Trait Preferences. *FRONTIERS IN SUSTAINABLE FOOD SYSTEMS* 5, (2021).
83. Courtois, B. *et al.* *Breeding better rainfed rice varieties through farmer participation: some early lessons from Eastern India*. http://agritrop.cirad.fr/392576/ (1998).
84. Ewell, H. Breeding for impact: perspectives on gender-responsive cassava breeding in Nigeria. *Gender, Technology and Development* 25, 217–232 (2021).
85. Soleri, D. & Cleveland, D. A. *Breeding for quantitative variables. Part 1: Farmers_ and scientists_ knowledge and practice in variety choice and plant selection*. ftp://ftp.fao.org/docrep/fao/012/i1070e/i1070e04.pdf (2009).
86. Gallardo, R. *et al.* Breeding Trait Priorities of the Blueberry Industry in the United States and Canada. *HORTSCIENCE* 53, 1021-+ (2018).
87. Gallardo, R. K. *et al.* Breeding trait priorities of the cranberry industry in the United States and Canada. *HortScience* 53, 1467–1474 (2018).
88. Marenya, P., Wanyama, R., Alemu, S. & Woyengo, V. Building Resilient Maize Production Systems With Stress-Adapted Varieties: Farmers' Priorities in Western Kenya. *FRONTIERS IN SUSTAINABLE FOOD SYSTEMS* 6, (2022).

89. Bentley, J. W. *et al. Cassava farmers' preferences for varieties and seed dissemination system in Nigeria: gender and regional perspectives*. (International Institute of Tropical Agriculture, 2017).
90. Teeken, B. *et al.* Cassava Trait Preferences of Men and Women Farmers in Nigeria: Implications for Breeding. *ECONOMIC BOTANY* 72, 263–277 (2018).
91. Bechoff, A. *et al.* Cassava traits and end-user preference: Relating traits to consumer liking, sensory perception, and genetics. *Critical Reviews in Food Science and Nutrition* 58, 547–567 (2018).
92. Ishikawa, H. *et al.* Characteristics of farmers' selection criteria for cowpea (Vigna unguiculata)varieties differ between north and south regions of Burkina Faso. *EXPERIMENTAL AGRICULTURE* 56, 94–103 (2020).
93. Pircher, T. *et al. Characterizing Nigeria's cassava seed system and the use of planting material in three farming communities*. https://cgspace.cgiar.org/handle/10568/106314 (2019).
94. Adinsi, L. *et al.* Characterizing quality traits of boiled yam: texture and taste for enhanced breeding efficiency and impact. *JOURNAL OF THE SCIENCE OF FOOD AND AGRICULTURE* (2023) doi:10.1002/jsfa.12589.
95. Ganeme, A. *et al.* Characterizing sorghum (Sorghum bicolor [L.] Moench) varieties diversity to identify those with contrasting traits of interest for intercropping systems in the Sudano-Sahelian zone of West Africa. *PLANT GENETIC RESOURCES-CHARACTERIZATION AND UTILIZATION* (2022) doi:10.1017/S1479262122000168.
96. Gimenez-Sanchis, A., Tarrega, A., Tarancon, P., Aleza, P. & Besada, C. Check-All-That-Apply Questions including the Ideal Product as a Tool for Selecting Varieties in Breeding Programs. A Case Study with Mandarins. *AGRONOMY-BASEL* 11, (2021).
97. Casals, J., Rivera, A., Sabate, J., del Castillo, R. & Simo, J. Cherry and Fresh Market Tomatoes: Differences in Chemical, Morphological, and Sensory Traits and Their Implications for Consumer Acceptance. *AGRONOMY-BASEL* 9, (2019).
98. Suhasini, K. *et al.* Chickpea Baseline and Early Adoption Surveys in South Asia Insights from TL-II (Phase-I) Project: Synthesis Report 2013. http://oar.icrisat.org/7930/ (2013).
99. Gallardo, R. K. Choice Experiments' Findings: A Tool for Fruit Agribusiness Managers' Decision Making. 14, 16 (2011).
100. Client-oriented breeding sparks a low-input green revolution in Bangladesh.
101. Almekinders, C. & Elings, A. Collaboration of farmers and breeders: Participatory crop improvement in perspective. *EUPHYTICA* 122, 425–438 (2001).
102. Virk, D. S., Singh, D. N., Prasad, S. C., Gangwar, J. S. & Witcombe, J. R. Collaborative and consultative participatory plant breeding of rice for the rainfed uplands of eastern India. *Euphytica* 132, 95–108 (2003).
103. Hong, Y. A., Gallardo, R. K., Silva, M. & Orozco, J. F. College Students' Preferences and Willingness to Pay for Fresh Apple Varieties in Peru. *Journal of Food Distribution Research* (2018) doi:10.22004/ag.econ.292175.
104. De Groote, H., Chege, C., Tomlins, K. & Gunaratna, N. Combining experimental auctions with a modified home-use test to assess rural consumers' acceptance of quality protein maize, a biofortified crop. *FOOD QUALITY AND PREFERENCE* 38, 1–13 (2014).
105. Abeyasekera, S., Ritchie, J. & Lawson-McDowall, J. Combining ranks and scores to determine farmers' preferences for bean varieties in southern Malawi. *EXPERIMENTAL AGRICULTURE* 38, 97–109 (2002).
106. Gibson, R. W. *et al. Community-based breeding ofsuperior, mosaic disease-resistantcassava in Ghana*. http://www.dfid.gov.uk/r4d/PDF/Outputs/RLPSRReview4.pdf

(2004).
107. Ishikawa, H., Drabo, I., Boukar, O., Fatokum, C. & Muranaka, S. Comparative Analysis of Farmers' Selection Criteria for Cowpea (Vigna unguiculata) Varieties in Niger and Burkina Faso. *JARQ-JAPAN AGRICULTURAL RESEARCH QUARTERLY* 53, 159–167 (2019).
108. Tchokponhoué, D. A. *et al.* Comparative analysis of management practices and end-users' desired breeding traits in the miracle plant [Synsepalum dulcificum (Schumach & Thonn.) Daniell] across ecological zones and sociolinguistic groups in West Africa. *Journal of Ethnobiology and Ethnomedicine* 17, (2021).
109. Malabayabas, M., Gauchan, D. & Pandey, S. Comparative analysis of rice variety adoption patterns in Eastern India and Central Luzon, Philippines. 28 (2011) doi:10.22004/ag.econ.290433.
110. Sanusi, M. S., Akinoso, R., Danbaba, N. & Hussein, J. Comparative studies of the effect of processing conditions on cooking and sensory properties of selected rice varieties. 22, (2022).
111. Trouche, G., Lancon, J., Acuna, S., Briones, B. & Thomas, G. Comparing decentralized participatory breeding with on-station conventional sorghum breeding in Nicaragua: II. Farmer acceptance and index of global value. *FIELD CROPS RESEARCH* 126, 70–78 (2012).
112. Courtois, B. *et al.* Comparing farmers and breeders rankings in varietal selection for low-input environments: A case study of rainfed rice in eastern India. *EUPHYTICA* 122, 537–550 (2001).
113. Baafi, E. *et al.* Constraints and Breeding Priorities for Increased Sweetpotato Utilization in Ghana. *Sustainable Agriculture Research* (2015) doi:10.22004/ag.econ.230300.
114. Mofokeng, M. A., Shimelis, H., Tongoona, P. & Laing, M. D. Constraints and varietal trait preferences of sorghum producers in South Africa. *Journal of Tropical Agriculture* 54, 7–15 (2016).
115. Hewavitharane, H. V. C., Warnakulasooriya, H. U. & Wajira Kumara, G. B. S. CONSTRAINTS TO EXPANSION OF COWPEA AND MUNGBEAN UNDER RAIN-FED FARMING IN ANURADHAPURA DISTRICT. 16 (2010) doi:10.22004/ag.econ.95541.
116. Bammite, D. *et al.* Constraints to production and preferred traits for taro (Colocasia esculenta) and new cocoyam (Xanthosoma mafaffa) in Togo, West Africa. *African Journal of Food, Agriculture, Nutrition and Development* 18, 13388–13405 (2018).
117. Nowakunda, K., Rubaihayo, P. R., Ameny, M. & Tushemereirwe, W. K. Consumer acceptability of introduced bananas in Uganda. *INFOMUSA; FRA* 9, 22–25 (2000).
118. De Groote, H. *et al.* Consumer acceptance of quality protein maize (QPM) in East Africa. *JOURNAL OF THE SCIENCE OF FOOD AND AGRICULTURE* 94, 3201–3212 (2014).
119. Unnevehr, L. J. CONSUMER DEMAND FOR RICE GRAIN QUALITY AND RETURNS TO RESEARCH ON QUALITY IMPROVEMENT. 14 (1984) doi:10.22004/ag.econ.279004.
120. Lewers, K., Newell, M., Park, E. & Luo, Y. Consumer preference and physiochemical analyses of fresh strawberries from ten cultivars. *INTERNATIONAL JOURNAL OF FRUIT SCIENCE* 20, S733–S756 (2020).
121. Klasener, G., Ribeiro, N., Casagrande, C. & Arns, F. Consumer preference and the technological and nutritional quality of different bean colours. *ACTA SCIENTIARUM-AGRONOMY* 42, (2020).
122. Ribeiro, N., Casagrande, C., Mezzomo, H., Klasener, G. & Steckling, S. Consumer preference and the technological, cooking and nutritional quality of carioca beans. *SEMINA-CIENCIAS AGRARIAS* 40, 651–664 (2019).

123. Folorunsho, W. & Ayinde, O. Consumer Preference of Banana(Musa Spp) in Kwara State. *international society of horticultural science* 879, 89–93 (2010).
124. Moyo, M. *et al.* Consumer Preference Testing of Boiled Sweetpotato Using Crowdsourced Citizen Science in Ghana and Uganda. *FRONTIERS IN SUSTAINABLE FOOD SYSTEMS* 5, (2021).
125. Cerda, A., Garcia, L., Ortega-Farias, S. & Ubilla, A. Consumer preferences and willingness to pay for organic apples. *CIENCIA E INVESTIGACION AGRARIA* 39, 47–59 (2012).
126. Mishili, F. J., Temu, A. A., Fulton, J. R. & Lowenberg-DeBoer, J. CONSUMER PREFERENCES AS DRIVERS OF THE COMMON BEAN TRADE IN TANZANIA: A MARKETING PERSPECTIVE. 18 pages (2009) doi:10.22004/ag.econ.48644.
127. Zamzami, L., Andrini, A. & Budiyati, E. Consumer Preferences for a New Variety of Grapes (Vitis vinifera) Paras 61. *Annals of Biology (Hissar)* 36, 159–162 (2020).
128. Carrillo-Rodriguez, L. *et al.* Consumer preferences for apple quality traits. 17 (2013) doi:10.22004/ag.econ.150503.
129. Gao, Z. *et al.* Consumer Preferences for Fresh Citrus: Impacts of Demographic and Behavioral Characteristics. 14, 17 (2011).
130. Causse, M. *et al.* Consumer Preferences for Fresh Tomato at the European Scale: A Common Segmentation on Taste and Firmness. *JOURNAL OF FOOD SCIENCE* 75, S531–S541 (2010).
131. Mishili, F. J. *et al.* CONSUMER PREFERENCES FOR QUALITY CHARACTERISTICS ALONG THE COWPEA VALUE CHAIN IN NIGERIA, GHANA AND MALI. 27 (2007) doi:10.22004/ag.econ.28684.
132. Gonzalez, C. & Johnson, N. L. Consumer preferences for table cassava characteristics in Pernambuco, Brazil. *Revista de Economia e Agronegócio / Brazilian Review of Economics and Agribusiness* 20 (2009) doi:10.22004/ag.econ.94839.
133. Sundari & Roini, C. Consumer Preferences of Local Durian (Durio zibethinus Murr.) In West Halmahera Island Based on Agronomic Characteristics. in vol. 2021 (2018).
134. IOP, Salmiah, Nauli, O. & Sihombing, L. Consumer preferences toward local rice in Medan. in *International Conference on Agriculture, Environment and Food Security (AEFS) 2019* vol. 454 012004-Article No.: 012004 (2020).
135. Leonard, R. L. & Wadsworth, J. J. Consumer Preferences: A Guide to Connecticut Apple Marketing. 25 (1989) doi:10.22004/ag.econ.25203.
136. Del Carmen, D., Esguerra, E. & Gerance, A. Consumer Purchasing Behavior for Fresh Soursop (Annona muricata L.): Evidence from Metro Manila and CALABARZON, Philippines. *PHILIPPINE AGRICULTURAL SCIENTIST* 103, 132–139 (2020).
137. Akankwasa, K., Tushemereirwe, W. & Ragama, P. Consumer willingness to pay for introduced dessert bananas in Uganda. *African Crop Science Journal (ISSN: 1021-9730) Vol 16 Num 4* 16, (2010).
138. Gilbert, J. *et al.* Consumer-assisted Selection of Blueberry Fruit Quality Traits. *HORTSCIENCE* 49, 864–873 (2014).
139. Syamsuri, R., Hanifa, A. P. & Yuniarsih, E. T. Consumers characteristics and their preferences toward local durians in South Sulawesi. in vol. 2616 (2023).
140. Barlagne, C., Cornet, D., Blazy, J., Diman, J. & Ozier-Lafontaine, H. Consumers' preferences for fresh yam: a focus group study. *FOOD SCIENCE & NUTRITION* 5, 54–66 (2017).
141. Bairagi, S., Mohanty, S. & Custodio, M. Consumers' preferences for rice attributes in Cambodia: a choice modeling approach. *JOURNAL OF AGRIBUSINESS IN DEVELOPING*

AND EMERGING ECONOMIES 9, 94–108 (2019).
142. Baco, M. N., Affoukouk, T., Moumouni, I., Yallou, C. & Abdoulaye, T. Contribution des femmes a ladoption des varietes de mais tolerantes a la secheresse au Nord Benin. *Science et Technique, Lettres, Sciences Sociales et Humaines* (2014).
143. Lunduka, R., Fisher, M. & Snapp, S. Could farmer interest in a diversity of seed attributes explain adoption plateaus for modern maize varieties in Malawi? *FOOD POLICY* 37, 504–510 (2012).
144. Karikari, B. *et al.* Cowpea cropping systems, traits preference and production constraints in the upper west region of Ghana: farmers' consultation and implications for breeding. *CABI AGRICULTURE & BIOSCIENCE* 4, (2023).
145. Knight Jr., R. J. CRITERIA FOR EVALUATING IMPORTANT FRUIT CHARACTERS IN MANGO (Mangifera indica L.) GERMPLASM. 5 (1985) doi:10.22004/ag.econ.261372.
146. van Etten, J. *et al.* Crop variety management for climate adaptation supported by citizen science. *Proceedings of the National Academy of Sciences* 116, 4194–4199 (2019).
147. Wendmu, T. *et al.* Cultural Effects on Sorghum Varieties Grown, Traits Preferred, and Seed Management Practices in Northern Ethiopia. *ECONOMIC BOTANY* 76, 233–249 (2022).
148. Witcombe, J. R. Decentralization versus farmer participation in plant breeding : some methodology issues. in (Centro Internacional de Agricultura Tropical, 1997).
149. Ceccarelli, S. & Grando, S. Decentralized-participatory plant breeding: an example of demand driven research. *Euphytica* 155, 349–360 (2007).
150. Lassoued, R. & Smyth, S. Decision factors influencing new variety adoption in western Canada by the seed industry. *CANADIAN JOURNAL OF PLANT SCIENCE* 103, 214–227 (2023).
151. Khachatryan, H. & Rihn, A. Defining U. S. Consumers' (mis)perceptions of Pollinator Friendly Labels: An Exploratory Study. *International Food and Agribusiness Management Review* (2018) doi:10.22004/ag.econ.269672.
152. Dalton, T. J., Yesuf, M. & Muhammad, L. Demand for Drought Tolerance in Africa: Selection of Drought Tolerant Maize Seed using Framed Field Experiments. 29 (2011) doi:10.22004/ag.econ.103712.
153. Motagi, B. N., Vabi, M. B., Ajeigbe, H. A., Echekwu, C. A. & Mohammed, S. G. Designing effective groundnut breeding strategies through farmers-breeder interactions in Northern Nigeria. in 248–249 (2016).
154. IOP & Sembiring, A. Determinant factors for Brebes shallot farmers in selecting shallot varieties (Case study in Brebes, Central Java Indonesia). in vol. 58 (2017).
155. Rai, P. & Bajgai, Y. Determinants Influencing Selection of Potato Varietal Technology and the Role of Gender in Farm Decisions in Bhutan. *POTATO RESEARCH* (2022) doi:10.1007/s11540-022-09607-3.
156. Cholo, M., Marisennayya, S., Bojago, E., Leja, D. & Divya, R. K. Determinants of adoption and intensity of improved haricot bean (Phaseolus vulgaris L.) varieties: A Socio-agronomic study from southern Ethiopia. *Journal of Agriculture and Food Research* 13, (2023).
157. Bagheri, A. Determinants of Adoption of Mini-Tuber Seed Potato: A Case in Ardabil Province of Iran. 05, (2015).
158. Laizer, J. S., Baharanyi, N. R., Zabawa, R. & Kadigi, R. M. J. Determinants of Consumer Preference for and Expenditure on Rice in the Kilimanjaro Region, Tanzania. 06, (2018).
159. Ortmann, G., Wale, E. & Tushemereirwe, W. Determinants of consumers' willingness to

purchase East African Highland cooking banana hybrids in Uganda. *African journal of agricultural research* 8, 780–791 (2013).
160. Nindjin, C. *et al.* Determination of relevant sensory properties of pounded yams (Dioscorea spp.) using a locally based descriptive analysis methodology. *Food Quality and Preference* 18, 450–459 (2007).
161. Singh, S. *et al.* Development and Promotion of an Informal and Formal Seed System Through Farmer Participatory Seed Production of Pigeonpea (Cajanus cajan L.) in Uttar Pradesh, India. *AGROECOLOGY AND SUSTAINABLE FOOD SYSTEMS* 37, 531–549 (2013).
162. Mwanga, R. *et al.* Development of a food product profile for boiled and steamed sweetpotato in Uganda for effective breeding. *INTERNATIONAL JOURNAL OF FOOD SCIENCE AND TECHNOLOGY* 56, 1385–1398 (2021).
163. Beekham, A., Ramdin, R., Deen, S., Jackson, T. & Nandlal, S. DEVELOPMENT OF AN OPEN POLLINATED PUMPKIN VARIETY (CES STARZ) WITH UNIFORM SHAPE, SKIN COLOUR AND QUALITY CHARACTERS FOR THE EXPORT MARKET. 10 (2013) doi:10.22004/ag.econ.253450.
164. Gridley, H. E., Jones, M. P. & Wopereis-Pura, M. Development of New Rice for Africa (NERICA) and participatory varietal selection.
165. Manu-Aduening, J. A. *et al.* Development of superior cassava cultivars in Ghana by farmers and scientists: The process adopted, outcomes and contributions and changed roles of different stakeholders. *Euphytica* 150, 47–61 (2006).
166. Okot, F., Laing, M., Shimelis, H. & de Milliano, W. Diagnostic Appraisal of the Sorghum Farming System and Breeding Priorities in Sierra Leone. *SUSTAINABILITY* 14, (2022).
167. Gurmu, F., Hussein, S. & Laing, M. Diagnostic assessment of sweetpotato Production in ethiopia: Constraints, post-harvest handling and farmers' preferences. *Research on Crops* 16, 104–115 (2015).
168. Misiko, M. Dilemma in participatory selection of varieties. *AGRICULTURAL SYSTEMS* 119, 35–42 (2013).
169. Alhassan, A., Salifu, H. & Adebanji, A. Discriminant analysis of farmers adoption of improved maize varieties in Wa Municipality, Upper West Region of Ghana. *SPRINGERPLUS* 5, (2016).
170. Abebe, G., Bijman, J., Pascucci, S., Omta, S. & Tsegaye, A. *Diverging quality preferences along the supply chain: implications for variety choice by potato growers in Ethiopia*. *QUALITY AND INNOVATION IN FOOD CHAINS: LESSONS AND INSIGHTS FROM AFRICA* 140 (2016). doi:10.3920/978-90-8686-825-4_610.3920/978-90-8686-825-4.
171. Nankya, R. *et al.* Diversity in Nutrient Content and Consumer Preferences of Sensory Attributes of Peanut (Arachis hypogaea L.) Varieties in Ugandan Agroecosystems. *SUSTAINABILITY* 13, (2021).
172. Calingacion, M. *et al.* Diversity of global rice markets and the science required for consumer-targeted rice breeding. *PLoS ONE* 9, (2014).
173. Dansi, A., Adoukonou-Sagbadja, H. & Vodouhe, R. Diversity, conservation and related wild species of Fonio millet (Digitaria spp.) in the northwest of Benin. *GENETIC RESOURCES AND CROP EVOLUTION* 57, 827–839 (2010).
174. Adejumobi, I. *et al.* Diversity, trait preferences, management and utilization of yams landraces (Dioscorea species): an orphan crop in DR Congo. *SCIENTIFIC REPORTS* 12, (2022).
175. Witcombe, J. Do farmer-participatory methods apply more to high potential areas than to marginal ones? *OUTLOOK ON AGRICULTURE* 28, 43–49 (1999).

176. Belay, G. Does client-oriented plant breeding work? *CAB Reviews: Perspectives in Agriculture, Veterinary Science, Nutrition and Natural Resources* 4, 1–7 (2009).
177. Anbazhagan, K. *et al.* Dual-Purpose Sorghum: A Targeted Sustainable Crop-Livestock Intervention for the Smallholder Subsistence Farming Communities of Adilabad, India. *FRONTIERS IN SUSTAINABLE FOOD SYSTEMS* 6, (2022).
178. Akankwasa, K., Ortmann, G. & Wale, E. Early-Stage Adoption of Improved Banana " Matooke " Hybrids in Uganda: A Count Data Analysis Based on Farmers' Perceptions. *International Journal of Innovation and Technology Management* 13, 1–26 (2015).
179. Adekambi, S., Okello, J., Abidin, P. & Carey, E. Effect of exposure to biofortified crops on smallholder farm household adoption decisions: The case of orange-fleshed sweetpotato in Ghana and Nigeria. *SCIENTIFIC AFRICAN* 8, (2020).
180. Adekambi, S. *et al.* Effect of varietal attributes on the adoption of an orange-fleshed sweetpotato variety in Upper East and Northern Ghana. *OUTLOOK ON AGRICULTURE* 49, 311–320 (2020).
181. Fufa, F., Grando, S., Kafawin, O., Shakhatreh, Y. & Ceccarelli, S. Efficiency of farmers' selection in a participatory barley breeding programme in Jordan. *PLANT BREEDING* 129, 156–161 (2010).
182. Agbola, F. W., Kelley, T. G., Bent, M. J. M. & Rao, P. P. ELICITING AND VALUING MARKET PREFERENCES WITH TRADITIONAL FOOD CROPS: THE CASE OF CHICKPEA IN INDIA. *International Food and Agribusiness Management Review* 15 (2002) doi:10.22004/ag.econ.34585.
183. Chakrabarti, A., Campbell, B. L. & Shonkwiler, V. Eliciting Consumer Preference and Willingness to Pay for Mushrooms: A Latent Class Approach. *Journal of Food Distribution Research* (2019) doi:10.22004/ag.econ.292182.
184. Otegbayo, B. *et al.* End-user preferences for pounded yam and implications for food product profile development. *INTERNATIONAL JOURNAL OF FOOD SCIENCE AND TECHNOLOGY* 56, 1458–1472 (2021).
185. Gaoh, B. *et al.* Establishing Breeding Priorities for Developing Biofortified High-Yielding Pearl Millet (Pennisetum glaucum (L.) R. Br.) Varieties and Hybrids in Dosso Region of Niger. *AGRONOMY-BASEL* 13, (2023).
186. Emmanuel, O. *et al.* Establishing the linkage between eba's instrumental and sensory descriptive profiles and their correlation with consumer preferences: implications for cassava breeding. *JOURNAL OF THE SCIENCE OF FOOD AND AGRICULTURE* (2023) doi:10.1002/jsfa.12518.
187. Choi, J. *et al.* Estimating Strawberry Attributes' Market Equilibrium Values. *HORTSCIENCE* 52, 742–748 (2017).
188. Choi, J. *et al.* Estimation of market equilibrium values for apple attributes. *CHINA AGRICULTURAL ECONOMIC REVIEW* 10, 135–151 (2018).
189. Aguessy, S. *et al.* Ethnobotanical characterization of scarlet eggplant (Solanum aethiopicum L.) varieties cultivated in Benin (West Africa). *JOURNAL OF AGRICULTURE AND FOOD RESEARCH* 5, (2021).
190. Soleri, D., Smith, S. & Cleveland, D. Evaluating the potential for farmer and plant breeder collaboration: A case study of farmer maize selection in Oaxaca, Mexico. *EUPHYTICA* 116, 41–57 (2000).
191. Laurie, S. & Magoro, M. Evaluation and release of new sweet potato varieties through farmer participatory selection. *AFRICAN JOURNAL OF AGRICULTURAL RESEARCH* 3, 672–676 (2008).
192. Letayo, E., Saadan, H., Mndolwa, S., Gupta, S. & Monyo, E. Evaluation of crop

performance and farmer preference for pearl millet varieties in Tanzania. in 65–70 (1996).
193. Geja, M. M. Evaluation of lentil varieties for adaptation and yield performance under midland ecology of kaffa zone, south-west Ethiopia. *International Journal of Agricultural Research, Innovation and Technology (IJARIT)* (2019) doi:10.22004/ag.econ.303809.
194. Dugje, I., Odo, P., Teli, I., Kamara, A. & Asiedu, E. Evaluation of multi-stress tolerant maize varieties for sustainable intensification in Northern Guinea Savanna of north eastern Nigeria. *MAYDICA* 59, 137–143 (2014).
195. Assefa, A., Bezabih, A., Germay, G., Alemayehu, T. & Lkaw, A. Evaluation of sorghum (Sorghum bicolor (L.) Moench) variety performance in the lowlands area of wag lasta, north eastern Ethiopia. *COGENT FOOD & AGRICULTURE* 6, (2020).
196. Abidin, P. *et al.* Evaluation of sweetpotato (Ipomoea batatas (L.) Lam.) germplasm from north-eastern Uganda through a farmer participatory approach. in 61–68 (2002). doi:10.17660/ActaHortic.2002.583.5.
197. Umesh, K. B. & Sakamma, S. Ex-ante attribute based technology development in agriculture: A case of finger millet in India. (2018) doi:10.22004/ag.econ.277148.
198. Amadi, C. *et al.* Exhibition Trial and Farmer Participatory Selection of New Late-blight Resistant B3C1 Potato Genotypes for Adaptation to Nigerian Conditions. in 128–133 (2015).
199. Karamura, D., Mgenzi, B., Karamura, E. & Sharrock, S. Exploiting indigenous knowledge for the management and maintenance of Musa biodiversity on farm. *African Crop Science Journal* 12, 67–74 (2004).
200. Quaye, W., Adofo, K., Madode, Y. & Abizari, A. Exploratory and multidisciplinary survey of the cowpea network in Tolon-Kumbungu district of Ghana: A food sovereignty perspective. *AFRICAN JOURNAL OF AGRICULTURAL RESEARCH* 4, 311–320 (2009).
201. Kassa, Y., Abie, A., Mamo, D. & Ayele, T. Exploring farmer perceptions and evaluating the performance of mung bean (Vigna radiata L) varieties in Amhara region, Ethiopia. *Heliyon* 8, (2022).
202. Asante, B. *et al.* Exploring Gender Differences in the Role of Trait Preferences among Stakeholders in the Rice Value Chain in Ghana. *SUSTAINABILITY* 15, (2023).
203. Geda, S. A. & Kühl, R. Exploring smallholder farmers' choices for climate-smart seed innovations: Empirical evidence from southern Ethiopia. *Sustainability (Switzerland)* 13, 1–18 (2021).
204. Mahadevan, R. & Asafu-Adjaye, J. Exploring the potential for green revolution: a choice experiment on maize farmers in Northern Ghana. *African Journal of Agricultural and Resource Economics* 15 (2015) doi:10.22004/ag.econ.211668.
205. Predieri, S. *et al.* Exploring through CATA (Check-All-That-Apply) method the Italian consumers' perception towards off-season nectarine imported from Chile. *EUROPEAN JOURNAL OF HORTICULTURAL SCIENCE* 86, 169–178 (2021).
206. Dahl, B. & Wilson, W. Factors affecting spring wheat variety choices: Comparisons between Canada and the United States. *CANADIAN JOURNAL OF AGRICULTURAL ECONOMICS-REVUE CANADIENNE D AGROECONOMIE* 47, 305–320 (1999).
207. Aitchedji, C., Tenkouano, A. & Coulibaly, O. Factors affecting the adoption of disease-resistant plantain and banana (MUSA SPP) Hybrids in Nigeria. in vol. 879 (2008).
208. Jemison, J. M., Jr., Sexton, P. & Camire, M. E. Factors influencing consumer preference of fresh potato varieties in maine. *American Journal of Potato Research* 85, 140–149 (2008).
209. Zawedde, B., Harris, C., Alajo, A., Hancock, J. & Grumet, R. Factors Influencing Diversity of Farmers' Varieties of Sweet Potato in Uganda: Implications for Conservation. *ECONOMIC BOTANY* 68, 337–349 (2014).

210. Masangano, C. & Miles, C. Factors influencing farmers' adoption of Kalima bean (Phaseolus vulgaris L.) variety in Malawi. *JOURNAL OF SUSTAINABLE AGRICULTURE* 24, 117–129 (2004).
211. Jogo, W., Bocher, T. & Grant, F. Factors influencing farmers' dis-adoption and retention decisions for biofortified crops: the case of orange-fleshed sweetpotato in Mozambique. *AGREKON* 60, 445–459 (2021).
212. Bissah, M. *et al.* Factors influencing rice production in the south-eastern belt of Ghana. *HELIYON* 8, (2022).
213. Nowakunda, K. & Tushemereirwe, W. Farmer acceptance of introduced banana genotypes in Uganda. *African Crop Science Journal* 12, 1–6 (2004).
214. Chianu, J., Vanlauwe, B., Mukalama, J., Adesina, A. & Sanginga, N. Farmer evaluation of improved soybean varieties being screened in five locations in Kenya: Implications for research and development. *AFRICAN JOURNAL OF AGRICULTURAL RESEARCH* 1, 143–150 (2006).
215. Thiele, G., Gardner, G., Torrez, R. & Gabriel, J. Farmer involvement in selecting new varieties: Potatoes in Bolivia. *EXPERIMENTAL AGRICULTURE* 33, 275–290 (1997).
216. Mekbib, F. Farmer participation in common bean genotype evaluation: The case of eastern Ethiopia. *EXPERIMENTAL AGRICULTURE* 33, 399–408 (1997).
217. Njukwe, E. *Farmer participation in Research-for-Development to enhance cassava production in Cameroon*. (Shoukadoh Book Sellers, 2016).
218. Egbadzor, K. F. *et al.* Farmer participation in selection within segregating populations of cowpea in Volta Region, Ghana. *Agriculture and Food Security* 4, (2015).
219. Witcombe, J. & Joshi, A. *Farmer participatory approaches for varietal breeding and selection linkages to the formal seed sector*. *Participatory plant breeding* (1996).
220. Joshi, A. & Witcombe, J. R. Farmer Participatory Approaches for Varietal Improvement.
221. Witcombe, J., Joshi, A., Joshi, K. & Sthapit, B. Farmer participatory crop improvement .1. Varietal selection and breeding methods and their impact on biodiversity. *EXPERIMENTAL AGRICULTURE* 32, 445–460 (1996).
222. Sthapit, B., Joshi, K. & Witcombe, J. Farmer participatory crop improvement .3. Participatory plant breeding, a case study for rice in Nepal. *EXPERIMENTAL AGRICULTURE* 32, 479–496 (1996).
223. Witcombe, J. R. & Joshi, A. Farmer participatory crop improvement methods. *Crop Improvement* 23, 112–122 (1996).
224. Joshi, A. & Witcombe, J. R. Farmer participatory crop improvement. II. Participatory varietal selection, a case study in India. *Experimental Agriculture* 32, 461–477 (1996).
225. Foti, R., Mapiye, C., Mutenje, M., Mwale, M. & Mlambo, N. Farmer participatory screening of maize seed varieties for suitability in risk prone, resource-constrained smallholder farming systems of Zimbabwe. *AFRICAN JOURNAL OF AGRICULTURAL RESEARCH* 3, 180–185 (2008).
226. Angarawai, I. I. *et al.* Farmer participatory varietal selection in pearl millet: Experience across some states of Northern Nigeria. *African Journal of Agricultural Research* 11, 1421–1425 (2016).
227. Thangapandian, R., Sumathi, P., Yuvaraja, A., Joseph, M. & Sanjivkumar, V. Farmer participatory varietal selection in pearl millet: Experience in vertisol tract of Southern districts of Tamil Nadu. *Electronic Journal of Plant Breeding* 8, 870–873 (2017).
228. Mitchell, J., Sipaseuth & Fukai, S. Farmer participatory variety selection conducted in high- and low-toposequence multi-location trials for improving rainfed lowland rice in Lao PDR. *CROP & PASTURE SCIENCE* 65, 655–666 (2014).

229. Kaguongo, W. *et al. Farmer practices and adoption of improved potato varieties in Kenya and Uganda*. (2008).
230. Sanchez-Toledano, B., Kallas, Z. & Gil-Roig, J. Farmer preference for improved corn seeds in Chiapas, Mexico: A choice experiment approach. *SPANISH JOURNAL OF AGRICULTURAL RESEARCH* 15, (2017).
231. Chiwona-Karltun, L. *et al.* Farmer Preference, Utilization, and Biochemical Composition of Improved Cassava (Manihot esculenta Crantz) Varieties in Southeastern Africa. *ECONOMIC BOTANY* 69, 42–56 (2015).
232. Snapp, S. & Silim, S. Farmer preferences and legume intensification for low nutrient environments. *PLANT AND SOIL* 245, 181–192 (2002).
233. Stanley Peter, D. Farmer Preferences and Market Integration of Cowpea in Uganda. 90 (2013) doi:10.22004/ag.econ.243442.
234. Ndjeunga, J. *et al.* Farmer preferences for groundnut traits and varieties in West Africa: Cases of Mali, Niger and Nigeria.Working Paper Series no. 27. http://oar.icrisat.org/221/ (2010).
235. Maligalig, R., Umbeger, W., Demont, M. & Peralta, A. Farmer preferences for rice varietal trait improvements in Nueva Ecija, Philippines: A latent class cluster approach. (2018) doi:10.22004/ag.econ.277476.
236. Nakyewa, B. *et al.* Farmer preferred traits and genotype choices in Solanum aethiopicum L., Shum group. *JOURNAL OF ETHNOBIOLOGY AND ETHNOMEDICINE* 17, (2021).
237. Abebrese, S. O. *et al.* Farmer Preferred Traits and Potential for Adoption of Hybrid Rice in Ghana. *Sustainable Agriculture Research* (2019) doi:10.22004/ag.econ.301900.
238. Horna, J. D., Smale, M. & von Oppen, M. Farmer Willingness to Pay for Seed-Related Information: Rice Varieties in Nigeria and Benin. 55 (2005) doi:10.22004/ag.econ.58587.
239. Annicchiarico, P., Russi, L., Romani, M., Pecetti, L. & Nazzicari, N. Farmer-participatory vs. conventional market-oriented breeding of inbred crops using phenotypic and genome-enabled approaches: A pea case study. *FIELD CROPS RESEARCH* 232, 30–39 (2019).
240. Adeniji, O. T. & Aloyce, A. Farmer's Knowledge of Horticultural Traits and Participatory Selection of African Eggplant Varieties (Solanum aethiopicum) in Tanzania. *Tropicultura* 30, 185–191 (2012).
241. IOP, Muslimin, Halil, W. & Dewayani, W. Farmer's response to high yielding rice varieties in South Sulawesi (case study of Takalar). in vol. 484 (2020).
242. Rafiq, M. *et al.* Farmer's participatory varietal selection in Japonica rice (Oryza sativa L.) in Kashmir valley. *Sabrao Journal of Breeding and Genetics* 48, 200–209 (2016).
243. Cardona, T. J., Speelman, S. & Sanou, E. Farmers attitudes towards GMO crops: comparison of attitudes towards first and second generation crops in Burkina Faso. (2018) doi:10.22004/ag.econ.276968.
244. ADESINA, A. & BAIDUFORSON, J. FARMERS PERCEPTIONS AND ADOPTION OF NEW AGRICULTURAL TECHNOLOGY - EVIDENCE FROM ANALYSIS IN BURKINA-FASO AND GUINEA, WEST-AFRICA. *AGRICULTURAL ECONOMICS* 13, 1–9 (1995).
245. Mansaray, B., Jin, S., Yuan, R. & Li, H. Farmers Preferences for Attributes of Seed Rice in Sierra Leone: A Best-Worst Scaling Approach. (2018) doi:10.22004/ag.econ.277552.
246. Acheampong, P. P., Owusu, V. & Nurah, G. K. Farmers Preferences for Cassava Variety Traits: Empirical Evidence from Ghana. 21 (2013) doi:10.22004/ag.econ.161633.
247. Adjei-Nsiah, S. *et al.* Farmers' agronomic and social evaluation of productivity, yield and N-2-fixation in different cowpea varieties and their subsequent residual N effects on a succeeding maize crop. *NUTRIENT CYCLING IN AGROECOSYSTEMS* 80, 199–209

(2008).
248. Mekbib, F. Farmers' Breeding of Sorghum [Sorghum bicolor (L.) Moench] in the Center of Diversity, Ethiopia: II. Selection Process, Criteria and Methods. *Journal of New Seeds* 9, 234–265 (2008).
249. Joshi, G. & Bauer, S. Farmers' choice of the modern rice varieties in the rainfed ecosystem of Nepal. *JOURNAL OF AGRICULTURE AND RURAL DEVELOPMENT IN THE TROPICS AND SUBTROPICS* 107, 129–138 (2006).
250. Sibiya, J., Tongoona, P., Derera, J. & Makandaa, I. Farmers' desired traits and selection criteria for maize varieties and their implications for maize breeding: A case study from Kwazulu-Natal Province, South Africa. *Journal of Agriculture and Rural Development in the Tropics and Subtropics* 114, 39–49 (2013).
251. Mulatu, E. & Zelleke, H. Farmers' highland maize (Zea mays L.) selection criteria: Implication for maize breeding for the Hararghe highlands of eastern Ethiopia. *EUPHYTICA* 127, 11–30 (2002).
252. Danial, D., Parlevliet, J., Almekinders, C. & Thiele, G. Farmers' participation and breeding for durable disease resistance in the Andean region. *EUPHYTICA* 153, 385–396 (2007).
253. Mandal, N. P., Sinha, P. K., Singh, R. K., Variar, M. & Atlin, G. N. *Farmers' participatory breeding for upland rice in eastern India*. https://scholar.google.com/scholar_lookup?title=Farmers%27+participatory+breeding+for+upland+rice+in+eastern+India&author=Mandal%2C+N.+P&publication_year=2002 (2002).
254. Singh, A. Farmers' participatory crop improvement: The need of the hour. *NATIONAL ACADEMY SCIENCE LETTERS-INDIA* 26, 119–137 (2003).
255. Adeniji, O. T. & Aloyce, A. Farmers' Participatory Identification of Horticultural Traits: Developing Breeding Objectives for Vegetable Amaranth in Tanzania. *Journal of Crop Improvement* 27, 309–318 (2013).
256. Letting, F., Venkataramana, P. & Ndakidemi, P. Farmers' Participatory Plant Selection of Lablab (Lablab purpureus (L.) Sweet) in Tanzania. *FRONTIERS IN PLANT SCIENCE* 13, (2022).
257. Najeeb, S. *et al.* Farmers' participatory selection of new rice varieties to boost production under temperate agro-ecosystems. *JOURNAL OF INTEGRATIVE AGRICULTURE* 17, 1307–1314 (2018).
258. Singh, Y. *et al.* Farmers' Participatory Varietal Selection: A Sustainable Crop Improvement Approach for the 21st Century. *AGROECOLOGY AND SUSTAINABLE FOOD SYSTEMS* 38, 427–444 (2014).
259. Akintunde, O. & Obayelu, O. Farmers' perception of on-farm conservation of cassava biodiversity in Ogun State, Nigeria. *INTERNATIONAL FOOD RESEARCH JOURNAL* 23, 2265–2270 (2016).
260. Adesina, A. A. & Seidi, S. Farmers' perceptions and adoption of new agricultural technology: analysis of modern mangrove rice varieties in Guinea Bissau. *Quarterly Journal of International Agriculture* 34, 358–371 (1995).
261. Mukanga, M., Derera, J., Tongoona, P. & Laing, M. D. Farmers' perceptions and management of maize ear rots and their implications for breeding for resistance. *African Journal of Agricultural Research* 6, 4544–4554 (2011).
262. Efisue, A. *et al.* Farmers' perceptions on rice varieties in Sikasso region of Mali and their implications for rice breeding. *JOURNAL OF AGRONOMY AND CROP SCIENCE* 194, 393–400 (2008).
263. Adewumi, A. S. *et al.* Farmers' perceptions on varietal diversity, trait preferences and

diversity management of bush yam (Dioscorea praehensilis Benth.) in Ghana. *Scientific African* 12, e00808-Article No.: e00808 (2021).
264. Agyeman, K. *et al.* Farmers' perceptions, constraints and preferences for improved Bambara groundnut varieties in Ghana. *JOURNAL OF AGRICULTURE AND FOOD RESEARCH* 3, (2021).
265. Ibrahim, A. *et al.* Farmers' practices, utilization, conservation and marketing of Bambara groundnut (Vigna subterranea (L.) Verdc.) in Dosso Region, Western Niger. *GENETIC RESOURCES AND CROP EVOLUTION* 65, 1907–1914 (2018).
266. Pandit, D. *et al.* Farmers' Preference and Informal Seed Dissemination of First Ug99 Tolerant Wheat Variety in Bangladesh. *CZECH JOURNAL OF GENETICS AND PLANT BREEDING* 47, S160–S164 (2011).
267. Onugo, T. N. & Onyeneke, R. U. Farmers' Preference and Willingness to Pay for Climate-Smart Rice Varieties in Uzo-Uwani Local Government Area of Enugu State, Nigeria. *Ekologia Bratislava* 41, 262–271 (2022).
268. Laborte, A. *et al.* Farmers' Preference for Rice Traits: Insights from Farm Surveys in Central Luzon, Philippines, 1966-2012. *PLOS ONE* 10, (2015).
269. Al Mahmud, A. *et al.* Farmers' Preference, Yield, and GGE-Biplot Analysis-Based Evaluation of Four Sweet Potato (Ipomoea batatas L.) Varieties Grown in Multiple Environments. *SUSTAINABILITY* 13, (2021).
270. Miriti, P., Regassa, M., Ojiewo, C. & Melesse, M. Farmers' preferences and willingness to pay for traits of sorghum varieties: Informing product development and breeding programs in Tanzania. *JOURNAL OF CROP IMPROVEMENT* 37, 253–272 (2023).
271. Martey, E., Etwire, P., Adogoba, D. & Tengey, T. Farmers' preferences for climate-smart cowpea varieties: implications for crop breeding programmes. *CLIMATE AND DEVELOPMENT* 14, 105–120 (2022).
272. Madalla, N. A. *et al.* Farmers' preferences for East African highland cooking banana 'Matooke' hybrids and local cultivars. *Agriculture and Food Security* 12, (2023).
273. Coulibaly, M., Agossou, C. O. A., Akohoué, F., Sawadogo, M. & Achigan-Dako, E. G. Farmers' preferences for genetic resources of Kersting's groundnut [macrotyloma geocarpum (harms) maréchal and baudet] in the production systems of Burkina Faso and Ghana. *Agronomy* 10, (2020).
274. Basuki, R. S., Khaririyatun, N., Kirana, R. & Thamrin, M. Farmers' Preferences on the Quality of Indonesian Chili's Varieties for Developing New Varieties. in vol. 995 (2022).
275. Sharma, N. *et al.* Farmers' preferences to varietal attributes as an indicator for acceptance and adoption of aromatic rice (Oryza sativa) varieties. *INDIAN JOURNAL OF AGRICULTURAL SCIENCES* 87, 51–55 (2017).
276. Valombola, J., Awala, S. & Hove, K. FARMERS' PREFERENCES, SEED SOURCE, PRODUCTION CONSTRAINTS AND IMPROVEMENT NEEDS ASSESSMENT OF BAMBARA GROUNDNUT (VIGNA SUBTERRANEA [L.] VERDC.) IN NORTHERN RURAL OF NAMIBIA. *SCIENTIFIC PAPERS-SERIES MANAGEMENT ECONOMIC ENGINEERING IN AGRICULTURE AND RURAL DEVELOPMENT* 21, 789–797 (2021).
277. Rabe, M., Baoua, I. & Baributsa, D. Farmers' Preferred Genotype Traits and Socio-Economic Factors Influencing the Adoption of Improved Cowpea Varieties in South-Central Niger. *AGRONOMY-BASEL* 12, (2022).
278. Holtland, G. Farmers' priorities for new sorghum and pearl millet varieties based on on-farm trials in semi-arid Tanzania. in 71–80 (1996).
279. Abay, F., Waters-Bayer, A. & Bjornstad, A. Farmers' seed management and innovation in varietal selection: Implications for barley breeding in Tigray, northern Ethiopia. *AMBIO* 37,

312–320 (2008).
280. Lebot, V., Malapa, R. & Sardos, J. Farmers' selection of quality traits in cassava (Manihot esculenta Crantz) landraces from Vanuatu. *GENETIC RESOURCES AND CROP EVOLUTION* 62, 1055–1068 (2015).
281. Kidasi, P., Chao, D., Obudho, E. & Mwang'ombe, A. Farmers' Sources and Varieties of Cassava Planting Materials in Coastal Kenya. *FRONTIERS IN SUSTAINABLE FOOD SYSTEMS* 5, (2021).
282. Hossain, S. M. Farmers' Trait Preferences for Varietal Replacement: A study to boost rice productivity in Odisha, India. *Grassroots Journal of Natural Resources* 4, 13–23 (2021).
283. Madalla, N. *Farmers' traits preferences for improved banana cultivars in Tanzania and Uganda*. (2021).
284. Brush, S. & Meng, E. Farmers' valuation and conservation of crop genetic resources. *GENETIC RESOURCES AND CROP EVOLUTION* 45, 139–150 (1998).
285. Ibitoye, D. O. & Kolawole, A. O. Farmers' Appraisal on Okra [Abelmoschus esculentus (L.)] Production and Phenotypic Characterization: A Synergistic Approach for Improvement. *Frontiers in Plant Science* 13, (2022).
286. Akankwasa, K., Ortmann, G. F., Wale, E. & Tushemereirwe, W. K. Farmers' choice among recently developed hybrid banana varieties in Uganda: A multinomial logit analysis. *Agrekon* 52, 25–51 (2013).
287. Loko, Y. L. E. *et al.* Farmers' management of peanut (Arachis hypogaea L.) diversity, their varietal preference traits and uses in Southern and Central Benin. *Journal of Crop Science and Biotechnology* 23, 259–272 (2020).
288. Vindhiyavarman, P., Manivannan, N., Nigam, S. N. & Muralidharan, V. Farmers' participatory varietal selection in groundnut: a case study from Tamil Nadu, India. *Electronic Journal of Plant Breeding* 1, 878–881 (2010).
289. Abady, S., Shimelis, H. & Janila, P. Farmers' perceived constraints to groundnut production, their variety choice and preferred traits in eastern Ethiopia: implications for drought-tolerance breeding. *Journal of Crop Improvement* 33, 505–521 (2019).
290. Mutari, B., Sibiya, J., Bogweh Nchanji, E., Simango, K. & Gasura, E. Farmers' perceptions of navy bean (Phaseolus vulgaris L.) production constraints, preferred traits and farming systems and their implications on bean breeding: a case study from South East Lowveld region of Zimbabwe. *Journal of Ethnobiology and Ethnomedicine* 17, (2021).
291. Mehmood, K., Rehman, A. & Khan, A. Farmers' Perceptions, Awareness and Adoption of Improved Groundnut Varieties in Potwar Plateau of Pakistan. *Sarhad Journal of Agriculture* 37, 1364–1376 (2021).
292. Suvi, W. T., Shimelis, H. & Laing, M. Farmers' perceptions, production constraints and variety preferences of rice in Tanzania. *Journal of Crop Improvement* 1–18 (2020) doi:10.1080/15427528.2020.1795771.
293. Martin, I., Vranken, L. & Ugás, R. Farmers' Preferences to Cultivate Threatened Crop Varieties: Evidence from Peru. (2021) doi:10.22004/ag.econ.315216.
294. Kyebalyenda, T. *et al.* FARMERS' SELECTION CUES IN COWPEA FOR VEGETABLE USE IN EASTERN UGANDA. *African Journal of Food, Agriculture, Nutrition and Development* 22, 20197–20214 (2022).
295. Dixon, A. *et al.* Fast track participatory approach to release of elite cassava genotypes for various uses in Nigeria's cassava economy. *EUPHYTICA* 160, 1–13 (2008).
296. Gebreyohannes, A. *et al.* Finger millet production in ethiopia: Opportunities, problem diagnosis, key challenges and recommendations for breeding. *Sustainability (Switzerland)* 13, (2021).

297. Etten, J. V. *et al.* FIRST EXPERIENCES WITH A NOVEL FARMER CITIZEN SCIENCE APPROACH: CROWDSOURCING PARTICIPATORY VARIETY SELECTION THROUGH ON-FARM TRIADIC COMPARISONS OF TECHNOLOGIES (TRICOT). *Experimental Agriculture* 55, 275–296 (2019).
298. Mulwa, C. K., Mussa, H., Ogero, K., Makunde, G. S. & Andrade, M. I. *Gender-differentiated trait preferences for sweetpotato varieties in Mozambique. A Focus Group Discussion Report.* https://cgspace.cgiar.org/handle/10568/116772 (2021).
299. Effah-Manu, L., Wireko-Manu, F., Agbenorhevi, J., Maziya-Dixon, B. & Oduro, I. Gender-Disaggregated Consumer Testing and Descriptive Sensory Analysis of Local and New Yam Varieties. *FOODS* 12, (2023).
300. Dinssa, F. *et al.* Gender-disaggregated Farmers Participatory Variety Selection in Amaranth Multilocation Trials in Kenya and Tanzania. *HORTTECHNOLOGY* 32, 288–303 (2022).
301. Adam, R. I., Kandiwa, V., David, S. & Muindi, P. Gender-responsive approaches for enhancing the adoption of improved maize seed in Africa: a training manual for plant breeders and technicians. (2019).
302. Nchanji, E. B., Lutomia, C. K., Ageyo, O. C., Karanja, D. & Kamau, E. Gender-responsive participatory variety selection in kenya: Implications for common bean (phaseolus vulgaris l.) breeding in kenya. *Sustainability (Switzerland)* 13, (2021).
303. Krishna, V. & Veettil, P. Gender, caste, and heterogeneous farmer preferences for wheat varietal traits in rural India. *PLOS ONE* 17, (2022).
304. Lope-Alzina, D. Gendered production spaces and crop varietal selection: Case study in Yucatan, Mexico. *SINGAPORE JOURNAL OF TROPICAL GEOGRAPHY* 28, 21–38 (2007).
305. Were, W. V., Shanahan, P., Melis, R. & Omari, O. O. Gene action controlling farmer preferred traits in cassava varieties adapted to mid-altitude tropical climatic conditions of western Kenya. *Field Crops Research* 133, 113–118 (2012).
306. Mayuoni-Kirshenbaum, L., Bar-Ya'akov, I., Hatib, K., Holland, D. & Porat, R. Genetic diversity and sensory preference in pomegranate fruits. *FRUITS* 68, 517–524 (2013).
307. Singh, R., van Heusden, A., Kumar, R., Visser, R. & Yadav, R. Genetic Diversity of Mungbean (Vigna radiata L.) in Iron and Zinc Content as Impacted by Farmers' Varietal Selection in Northern India. *ECOLOGY OF FOOD AND NUTRITION* 52, 148–162 (2013).
308. Kidane, Y. *et al.* Genome Wide Association Study to Identify the Genetic Base of Smallholder Farmer Preferences of Durum Wheat Traits. *FRONTIERS IN PLANT SCIENCE* 8, (2017).
309. Gesesse, C. A. *et al.* Genomics-driven breeding for local adaptation of durum wheat is enhanced by farmers' traditional knowledge. *Proceedings of the National Academy of Sciences of the United States of America* 120, (2023).
310. Mukankusi, C. *et al.* Genomics, genetics and breeding of common bean in Africa: A review of tropical legume project. *Plant Breeding* 138, 401–414 (2019).
311. Batlang, U. *et al.* Germplasm evaluation for climate adaptation and drought tolerance: The cases of local varieties of maize in Zambia and cowpea in Botswana. *Biodiversity Ecol* 6, 272–281 (2018).
312. Trouche, G., vom Brocke, K., Aguirre, S. & Chow, Z. GIVING NEW SORGHUM VARIETY OPTIONS TO RESOURCE-POOR FARMERS IN NICARAGUA THROUGH PARTICIPATORY VARIETAL SELECTION. *EXPERIMENTAL AGRICULTURE* 45, 451–467 (2009).
313. Karunakaran, K. R. *et al.* Groundnut Baseline and Early-Adoption Surveys in South Asia: Insights from TL-II (Phase-1) Project: Synthesis Report 2013.

http://oar.icrisat.org/7932/ (2013).
314. Banla, E. M. *et al.* Groundnut production constraints and farmers' trait preferences: A pre-breeding study in Togo. *Journal of Ethnobiology and Ethnomedicine* 14, (2018).
315. Daudi, H., Shimelis, H., Laing, M., Okori, P. & Mponda, O. Groundnut production constraints, farming systems, and farmer-preferred traits in Tanzania. *Journal of Crop Improvement* 32, 812–828 (2018).
316. Li, Z. *et al.* Grower Willingness to Pay for Fruit Quality versus Plant Disease Resistance and Welfare Implications: The Case of Florida Strawberry. *JOURNAL OF AGRICULTURAL AND RESOURCE ECONOMICS* 45, 199-+ (2020).
317. Surdianto, Y., Sunandar, B. & Sutrisna, N. Growth and productivity of new superior rice varieties and respondents' preference in Majalengka Regency. in vol. 306 (2021).
318. Buergelt, D., von Oppen, M. & Yadavendra, J. P. Hedonic price analysis to guide breeding for upgrading an orphan crop in India and Nepal. 15 (2009) doi:10.22004/ag.econ.51758.
319. Brocke, K. *et al.* HELPING FARMERS ADAPT TO CLIMATE AND CROPPING SYSTEM CHANGE THROUGH INCREASED ACCESS TO SORGHUM GENETIC RESOURCES ADAPTED TO PREVALENT SORGHUM CROPPING SYSTEMS IN BURKINA FASO. *EXPERIMENTAL AGRICULTURE* 50, 284–305 (2014).
320. Martey, E. & Goldsmith, P. Heterogeneous demand for soybean quality. *AFRICAN JOURNAL OF AGRICULTURAL AND RESOURCE ECONOMICS-AFJARE* 15, 27–50 (2020).
321. Belay, G., Tefera, H., Getachew, A., Assefa, K. & Metaferia, G. Highly client-oriented breeding with farmer participation in the Ethiopian cereal tef [Eragrostis tef (Zucc.) Trotter]. *AFRICAN JOURNAL OF AGRICULTURAL RESEARCH* 3, 22–28 (2008).
322. Joshi, K. *et al.* Highly client-oriented breeding, using local preferences and selection, produces widely adapted rice varieties. *FIELD CROPS RESEARCH* 100, 107–116 (2007).
323. Orobiyi, A. *et al.* Horticultural practices and varietal diversity of chili pepper (Capsicum annuum L.) in Central and Northern Benin. *GENETIC RESOURCES AND CROP EVOLUTION* 64, 419–436 (2017).
324. Floro, V., Labarta, R., Lopez-Lavalle, L., Martinez, J. & Ovalle, T. Household Determinants of the Adoption of Improved Cassava Varieties using DNA Fingerprinting to Identify Varieties in Farmer Fields: A Case Study in Colombia. *JOURNAL OF AGRICULTURAL ECONOMICS* 69, 518–536 (2018).
325. Ogunleke, A. & Baiyegunhi, L. Households' acceptability of local (Ofada) rice based on quality attributes in South-West, Nigeria. *BRITISH FOOD JOURNAL* 121, 2233–2248 (2019).
326. Acheampong, P., Owusu, V. & Nurah, G. How does Farmer Preference matter in Crop variety Adoption ? The case of Improved Cassava varieties' Adoption in Ghana. *OPEN AGRICULTURE* 3, 466–477 (2018).
327. Atieno, E., Kilwinger, F., Almekinders, C. & Struik, P. How Kenyan Potato Farmers Evaluate the Seed: Implications for the Promotion of Certified Seed Potato. *POTATO RESEARCH* (2023) doi:10.1007/s11540-022-09602-8.
328. Iragaba, P. *et al.* Identification of cassava quality attributes preferred by Ugandan users along the food chain. *INTERNATIONAL JOURNAL OF FOOD SCIENCE AND TECHNOLOGY* 56, 1184–1192 (2021).
329. Gastelo, M. *et al.* Identification of Elite Potato Clones with Resistance to Late Blight Through Participatory Varietal Selection in Peru. *POTATO RESEARCH* 64, 611–634 (2021).
330. Kolech, S. *et al.* Identification of Farmer Priorities in Potato Production Through

Participatory Variety Selection. *AMERICAN JOURNAL OF POTATO RESEARCH* 92, 648–661 (2015).
331. Kemal, S. A. Identification of Farmer's Preferred Durum Wheat (Triticum durum L) Varieties in Southeastern Ethiopia. (2017).
332. Kwak, H. S., Kim, M., Lee, Y. & Jeong, Y. Identification of key sensory attributes for consumer acceptance and instrumental quality of aseptic-packaged cooked rice. *International Journal of Food Science & Technology* 50, 691–699 (2015).
333. Njoku, D. N. *et al.* Identification of Pro-vitamin A Cassava (Manihot esculenta Crantz) Varieties for Adaptation and Adoption through Participatory Research. *Journal of Crop Improvement* 28, 361–376 (2014).
334. Bellon, M. R. *et al.* IDENTIFYING APPROPRIATE GERMPLASM FOR PARTICIPATORY BREEDING: AN EXAMPLE FROM THE CENTRAL VALLEYS OF OAXACA, MEXICO. 19 (2000) doi:10.22004/ag.econ.46524.
335. Drabo, I. *et al.* IDENTIFYING FARMERS' PREFERENCES and CONSTRAINTS to PEARL MILLET PRODUCTION in the SAHEL and NORTH-SUDAN ZONES of BURKINA FASO. *Experimental Agriculture* 55, 765–775 (2019).
336. Mohammadi, R. *et al.* Identifying superior rainfed barley genotypes in farmers' fields using participatory varietal selection. *J. Crop Sci. Biotechnol.* 14, 281–288 (2011).
337. Thapa, D. B. *et al.* Identifying superior wheat cultivars in participatory research on resource poor farms. *Field Crops Research* 112, 124–130 (2009).
338. Macholdt, J. & Honermeier, B. Importance of variety choice: Adapting to climate change in organic and conventional farming systems in Germany. *OUTLOOK ON AGRICULTURE* 46, 178–184 (2017).
339. Kangile, R., Gebeyehu, S. & Mollel, H. Improved rice seed use and drivers of source choice for rice farmers in Tanzania. *JOURNAL OF CROP IMPROVEMENT* 32, 622–634 (2018).
340. Custodio, M., Demont, M., Laborte, A. & Ynion, J. Improving food security in Asia through consumer-focused rice breeding. *GLOBAL FOOD SECURITY-AGRICULTURE POLICY ECONOMICS AND ENVIRONMENT* 9, 19–28 (2016).
341. Dorward, P., Craufurd, P., Marfo, K., Dogbe, W. & Bam, R. Improving participatory varietal selection processes: participatory varietal selection and the role of informal seed diffusion mechanisms for upland rice in Ghana. *EUPHYTICA* 155, 315–327 (2007).
342. Fla State Hort Soc *et al.* In Pursuit of the Perfect Peach: Consumer-assisted Selection of Peach Fruit Traits. in vol. 128 35–45 (2015).
343. Paris, T. R. *et al.* Incorporating gender concerns in participatory rice breeding and varietal selection: Preliminary results from Eastern India. *In: Lilja, Nina; Ashby, Jacqueline Anne; Sperling, Louise (eds.). Assessing the impact of participatory research and gender analysis*.
344. Witcombe, J., Joshi, K., Rana, R. & Virk, D. Increasing genetic diversity by participatory varietal selection in high potential production systems in Nepal and India. *EUPHYTICA* 122, 575–588 (2001).
345. Virk, D., Chakraborty, M., Ghosh, J., Prasad, S. & Witcombe, J. Increasing the client orientation of maize breeding using farmer participation in eastern India. *EXPERIMENTAL AGRICULTURE* 41, 413–426 (2005).
346. Dao, A., Sanou, J., Gracen, V. & Danquah, E. Y. Indentifying farmers' preferences and constraints to maize production in two agro-ecological zones in Burkina Faso. *Agriculture and Food Security* 4, (2015).
347. Sugri, I. *et al.* Influence of current seed programme of Ghana on maize (Zea mays)

seed security. *Research Journal of Seed Science* 6, 29–39 (2013).
348. Causse, M., Buret, M., Robini, K. & Verschave, P. Inheritance of nutritional and sensory quality traits in fresh market tomato and relation to consumer preferences. *Journal of Food Science* 68, 2342–2350 (2003).
349. INSTITUTIONAL INNOVATIONS IN INDIA'S CROP IMPROVEMENT SYSTEM. (2009).
350. INSTITUTIONAL INNOVATIONS IN NEPAL'S CROP IMPROVEMENT SYSTEM. (2009).
351. Lazaro, A. & Ruiz-Aceituno, L. Instrumental Texture Profile of Traditional Varieties of Tomato (Solanum lycopersicum L.) and its Relationship to Consumer Textural Preferences. *Plant Foods for Human Nutrition (Dordrecht)* 76, (2021).
352. Fadda, C. *et al.* Integrating Conventional and Participatory Crop Improvement for Smallholder Agriculture Using the Seeds for Needs Approach: A Review. *Frontiers in Plant Science* 11, (2020).
353. Dufour, D., Hershey, C., Hamaker, B. R. & Lorenzen, J. Integrating end-user preferences into breeding programmes for roots, tubers and bananas. *International Journal of Food Science & Technology* 56, 1071–1075 (2021).
354. Misiko, M., Tittonell, P., Ramisch, J. J., Richards, P. & Giller, K. E. Integrating new soybean varieties for soil fertility management in smallholder systems through participatory research: Lessons from western Kenya. *Agricultural Systems* 97, 1–12 (2008).
355. Mucioki, M., Mucioki, S. & Johns, T. Intraspecific Diversity and Seed Management of Pearl Millet (Pennisetum glaucum) in Tharaka, Kenya: A Persistent and Valued Traditional Food Crop. *ECONOMIC BOTANY* 68, 397–409 (2014).
356. Karamura, E. *et al.* Introduced banana hybrids in Africa: seed systems, farmers' experiences and consumers' perspectives. in vol. 1114 239–243 (2016).
357. Bam, R. *et al.* Introducing improved cultivars: Understanding farmers' seed drying and storage practices in central Ghana. *EXPERIMENTAL AGRICULTURE* 43, 301–317 (2007).
358. Testen, A. *et al.* Introduction and Evaluation of Tomato Germplasm by Participatory Mother and Baby Trials in the Morogoro Region of Tanzania. *HORTSCIENCE* 51, 1467–1474 (2016).
359. Faye, M. D. INVESTIGATION OF KEY ASPECTS FOR THE SUCCESSFUL MARKETING OF COWPEAS IN SENEGAL. 151 (2005) doi:10.22004/ag.econ.28059.
360. Baco, M. N., Affoukouk, T., Moumouni, I. & Abdoulaye, T. Is taking gender into account for development and diffusion of agricultural innovations justified? The case of drought tolerant maize in Northern Benin. *Journal of Agricultural Extension and Rural Development* (2015) doi:10.5897/jaerd2015.0720.
361. Kim, R. B., Unterschultz, J. R. & Veeman, M. M. JAPANESE MILLERS' PREFERENCES FOR WHEAT AND FLOUR: A STATED PREFERENCE ANALYSIS. 12 (2000) doi:10.22004/ag.econ.36403.
362. Melfa & Workaye Farmer Community *et al.* Joining smallholder farmers' traditional knowledge with metric traits to select better varieties of Ethiopian wheat. *SCIENTIFIC REPORTS* 7, (2017).
363. De Groote, H. *et al.* Kenyan farmers appreciate the higher yield of 50% non-pollen producing Maize (Zea mays) hybrids. *EXPERIMENTAL AGRICULTURE* 59, (2023).
364. Nyaligwa, L., Hussein, S., Laing, M., Ghebrehiwot, H. & Amelework, B. A. Key maize production constraints and farmers' preferred traits in the mid-altitude maize agroecologies of northern Tanzania. *South African Journal of Plant and Soil* 34, 47–53 (2017).
365. Temudo, M. P. Knowledge interfaces: Rice varieties selection and the question of food security in Guinea-Bissau. *Revista de Ciencias Agrarias* 19, 69–95 (1996).

366. Diallo, C. *et al.* Learning from farmers to improve sorghum breeding objectives and adoption in Mali. *JOURNAL OF CROP IMPROVEMENT* 32, 829–846 (2018).
367. Humphries, S., Gallardo, O., Jimenez, J., Sierra, F. & Jiménez, J. LINKING SMALL FARMERS TO THE FORMAL RESEARCH SECTOR: LESSONS FROM A PARTICIPATORY BEAN BREEDING PROGRAMME IN HONDURAS. (2005).
368. Paris, T. R. *et al.* Listening to farmers' perceptions through participatory rice varietal selection: A case study in villages in Eastern Uttar Pradesh, India. in (Consultative Group on International Agricultural Research, 2001).
369. Omot, N., Spriggs, J. & Chang, H.-S. (Christie). Long-Distance Marketing of Sweet Potato from the Highlands of Papua New Guinea: An Analysis of Consumer Preferences and Supplier Responsiveness. 22 (2010) doi:10.22004/ag.econ.59110.
370. Ghebreagziabiher, F., Gorman, M., Burke, J., Struik, P. & Griffin, D. Looking backward to find a path forward for the sustainable flow of suitable potato varieties to Eritrean potato farmers. *INTERNATIONAL JOURNAL OF AGRICULTURAL SUSTAINABILITY* 20, 911–925 (2022).
371. Montes-Hernandez, S., Merrick, L. & Eguiarte, L. Maintenance of squash (Cucurbita spp.) landrace diversity by farmers' activities in Mexico. *GENETIC RESOURCES AND CROP EVOLUTION* 52, 697–707 (2005).
372. De Groote, H., Dema, G., Sonda, G. & Gitonga, Z. Maize for food and feed in East Africa-The farmers' perspective. *FIELD CROPS RESEARCH* 153, 22–36 (2013).
373. Lobulu, J., Shimelis, H., Laing, M. & Mushongi, A. Maize production constraints, traits preference and current Striga control options in western Tanzania: farmers' consultation and implications for breeding. *ACTA AGRICULTURAE SCANDINAVICA SECTION B-SOIL AND PLANT SCIENCE* 69, 734–746 (2019).
374. Gibson, R. *et al.* Maize seed selection by East African smallholder farmers and resistance to Maize streak virus. *ANNALS OF APPLIED BIOLOGY* 147, 153–159 (2005).
375. Marenya, P., Wanyama, R., Alemu, S., Westengen, O. & Jaleta, M. Maize variety preferences among smallholder farmers in Ethiopia: Implications for demand-led breeding and seed sector development. *PLOS ONE* 17, (2022).
376. Chataika, B. *et al.* Major Production Constraints and Spider Plant [Gynandropsis gynandra (L.) Briq.] Traits Preferences Amongst Smallholder Farmers of Northern Namibia and Central Malawi. *FRONTIERS IN SUSTAINABLE FOOD SYSTEMS* 6, (2022).
377. Puska, A., Nedeljkovic, M., Prodanovic, R., Vladisavljevic, R. & Suzic, R. Market Assessment of Pear Varieties in Serbia Using Fuzzy CRADIS and CRITIC Methods. *AGRICULTURE-BASEL* 12, (2022).
378. Okello, J. J. *et al.* Market Intelligence and Incentive-Based Trait Ranking for Plant Breeding: A Sweetpotato Pilot in Uganda. *Frontiers in Plant Science* 13, (2022).
379. Gallardo, R. K. *et al.* Market Intermediaries' Ratings of Importance for Rosaceous Fruits' Quality Attributes. *International Food and Agribusiness Management Review* 34 (2015) doi:10.22004/ag.econ.211657.
380. Nissen, R., Smith, L., Broadley, R. & Sisson, C. Marketing of custard apple in Australia: From grower to consumer. in 437–442 (2002). doi:10.17660/ActaHortic.2002.575.49.
381. Esuma, W., Nanyonjo, A. R., Miiro, R., Angudubo, S. & Kawuki, R. S. Men and women's perception of yellow-root cassava among rural farmers in eastern Uganda. *Agriculture and Food Security* 8, (2019).
382. Weltzien, E. & Christinck, A. Methodologies for priority setting in plant breeding programs. in *Plant breeding and farmer participation* 75–105 (2009).
383. Asfaw, A. *et al.* Micro-nutrient composition and end-user acceptable quality in potato in

Ethiopia. *ACTA AGRICULTURAE SCANDINAVICA SECTION B-SOIL AND PLANT SCIENCE* 68, 596–607 (2018).
384. Kassie, G. T. *et al.* Modeling Preference and Willingness to Pay for Drought Tolerance (DT) in Maize in Rural Zimbabwe. *World Development* 94, 465–477 (2017).
385. Alibu, S. *et al.* Modest Ag-Extension and Access to Seeds of Aromatic Rice Can Boost Returns of Smallholder Farmers in Uganda, A Case Study. *AGRICULTURE-BASEL* 12, (2022).
386. Andrade, N., Monteros-Altamirano, A., Bastidas, C. & Sorensen, M. Morphological, Sensorial and Chemical Characterization of Chilli Peppers (Capsicum spp.) from the CATIE Genebank. *AGRONOMY-BASEL* 10, (2020).
387. Achoja, F. O., Enujeke, E. C., Ogisi, O. D. & Overehirha, R. T. Multinomial regression analysis of yam (Dioscorea spp.) consumers' preferences and varietal diversification pattern in Nigeria. *Asian Journal of Agriculture and Rural Development* 10, 698–707 (2020).
388. Meressa, A. M. & Navrud, S. Not my cup of coffee: Farmers' preferences for coffee variety traits – Lessons for crop breeding in the age of climate change. *Bio-based and Applied Economics Journal* (2020) doi:10.22004/ag.econ.309254.
389. Dida, M. *et al.* Novel sources of resistance to blast disease in finger millet. *CROP SCIENCE* 61, 250–262 (2021).
390. Nyakurwa, C., Gasura, E., Mabasa, S., Rugare, J. & Setimela, P. On-farm agronomic performance and farmer preference of quality protein maize grown under conservation agriculture in Southern Africa: a case for Zimbabwe. *JOURNAL OF AGRICULTURAL SCIENCE* 160, 168–184 (2022).
391. Yadav, R. K. *et al.* On-Farm Diversity Assessment and Participatory Varietal Evaluation of Cold-Tolerant Rice in Mid-Hills of Nepal. *Journal of Crop Science and Biotechnology* 22, 403–414 (2019).
392. Williams, R. *et al.* On-farm evaluation of introduced maize varieties and their yield determining factors in East Timor. *FIELD CROPS RESEARCH* 137, 170–177 (2012).
393. Chintu, E., Monyo, E. & Gupta, S. On-farm evaluation of pearl millet varieties in Malawi for farmer preferences, grain yield, and food quality traits. in 27–33 (1996).
394. Bucheyeki, T., Shenkalwa, E., Mapunda, T. & Matata, L. On-farm evaluation of promising groundnut varieties for adaptation and adoption in Tanzania. *AFRICAN JOURNAL OF AGRICULTURAL RESEARCH* 3, 531–536 (2008).
395. Behera, U. K. & Jha, K. P. On-farm evaluation of promising rice varieties and their response to green manuring in the rainfed mid land and low land ecosystem of Kalahandi, Orissa. *Crop Research (Hisar)* 13, 267–276 (1997).
396. Oyekanmi, A. A., Okeleye, K. A. & Okonji, C. J. On-farm evaluation of rainfed lowland rice varieties at Olokose village, Odeda, Ogun State, Nigeria. *Journal of Agronomy* 7, 192–196 (2008).
397. Sogoba, B. *et al.* On-Farm Evaluation on Yield and Economic Performance of Cereal-Cowpea Intercropping to Support the Smallholder Farming System in the Soudano-Sahelian Zone of Mali. *AGRICULTURE-BASEL* 10, (2020).
398. Kinhoegbe, G. *et al.* On-farm management and participatory evaluation of pigeonpea (Cajanus cajan [L.] Millspaugh) diversity across the agro-ecological zones of the Republic of Benin. *JOURNAL OF ETHNOBIOLOGY AND ETHNOMEDICINE* 16, (2020).
399. Loko, Y. *et al.* On-Farm Management of Rice Diversity, Varietal Preference Criteria, and Farmers' Perceptions of the African (Oryza glaberrima Steud.) Versus Asian Rice (Oryza sativa L.) in the Republic of Benin (West Africa): Implications for Breeding and Conservation. *ECONOMIC BOTANY* 75, 1–29 (2021).

400. Loko, Y. *et al.* On-Farm Management of Soybean (Glycine max) Varietal Diversity in Southern and Central Regions of the Republic of Benin. *AGRICULTURAL RESEARCH* 11, 359–372 (2022).
401. Ssali, R. *et al.* On-farm participatory evaluation of East African highland banana 'Matooke' hybrids. *Acta Horticulturae* 879, 585–591 (2010).
402. Worku, M. *et al.* On-farm performance and farmers' participatory assessment of new stress-tolerant maize hybrids in Eastern Africa. *FIELD CROPS RESEARCH* 246, (2020).
403. Akohoué, F., Sibiya, J. & Achigan-Dako, E. G. On-farm practices, mapping, and uses of genetic resources of Kersting's groundnut [Macrotyloma geocarpum (Harms) Maréchal et Baudet] across ecological zones in Benin and Togo. *Genetic Resources and Crop Evolution* 66, 195–214 (2019).
404. BaiduForson, J. On-station farmer participatory varietal evaluation: A strategy for client-oriented breeding. *EXPERIMENTAL AGRICULTURE* 33, 43–50 (1997).
405. Asgedom, S., Struik, P. C., Heuvelink, E. P. & Araia, W. Opportunities and constraints of tomato production in Eritrea. *African Journal of Agricultural Research* 6, 956–967 (2011).
406. Nduwumuremyi, A., Melis, R., Shanahan, P. & Asiimwe, T. Participatory appraisal of preferred traits, production constraints and postharvest challenges for cassava farmers in Rwanda. *Food Security* 8, 375–388 (2016).
407. Horn, L., Shimelis, H. & Laing, M. Participatory appraisal of production constraints, preferred traits and farming system of cowpea in the northern Namibia: Implications for breeding. *Legume Research* 38, 691–700 (2015).
408. Asfaw, A., Almekinders, C., Blair, M. & Struik, P. Participatory approach in common bean (Phaseolus vulgaris L.) breeding for drought tolerance for southern Ethiopia. *PLANT BREEDING* 131, 125–134 (2012).
409. Nguyen, N. D. Participatory approach to crop improvement at the community level in Vietnam. in (Consultative Group on International Agricultural Research, 2001).
410. Buah, S. S. J., Denwar, N. N., Kanton, R. A. L. & Kombiok, J. M. Participatory approach to variety selection using soybean production in ghana as a model. *West African Journal of Applied Ecology* 28, 14–30 (2020).
411. Witcombe, J. R. Participatory Approaches to Plant Breeding and Selection.
412. Namugga, P., Melis, R., Sibiya, J. & Barekye, A. Participatory assessment of potato farming systems, production constraints and cultivar preferences in Uganda. *Australian Journal of Crop Science* 11, 932–940 (2017).
413. Muhinyuza, J. *et al.* Participatory Assessment of Potato Production Systems and Cultivar Development in Rwanda. *SUSTAINABILITY* 14, (2022).
414. Tekalign, A., Derera, J., Sibiya, J. & Fikre, A. Participatory assessment of production threats, farmers' desired traits and selection criteria of faba bean (Vicia faba L.) varieties: Opportunities for faba bean breeding in Ethiopia. *Indian Journal of Agricultural Research* 50, 295–302 (2016).
415. Thakur, R. Participatory Breeding in Rice.
416. Monyo, E. S., Ipinge, S. A., Heinrich, G. M. & Chinhema, E. Participatory breeding: Does it make a difference? lessons from Namibian pearl millet farmers. *In: Lilja, Nina; Ashby, Jacqueline Anne; Sperling, Louise (eds.). Assessing the impact of participatory research and gender analysis*.
417. Gyawali, S. *et al.* Participatory crop improvement and formal release of Jethobudho rice landrace in Nepal. *EUPHYTICA* 176, 59–78 (2010).
418. Tiwari, T. P., Virk, D. S. & Sinclair, F. L. Participatory crop improvement for intercropped maize on Bari land terraces with trees. in (Consultative Group on International Agricultural

Research, 2001).
419. Tiwari, Virk & Sinclair, &. Participatory Crop Improvement for Maize-Millet Intercropping in the mid-hills of the Himalayan Region R7281 Plant Sciences Research Programme Final Participatory Crop Improvement for Maize-Millet Intercropping in the mid-hills of the Himalayan Region.
420. Goyal, S. N., Joshi, A. & Witcombe, J. R. Participatory crop improvement in maize in Gujarat, India. in (Consultative Group on International Agricultural Research, 2001).
421. Kassa, Y., Ayele, T., Teferra, B. & Abie, A. Participatory Evaluation and Selection of Improved Food Barley Varieties in the Highland Potential Areas of Central Ethiopia. *Proceedings of the National Academy of Sciences India Section B - Biological Sciences* 92, 907–917 (2022).
422. Burman, D. *et al.* Participatory evaluation guides the development and selection of farmers' preferred rice varieties for salt- and flood-affected coastal deltas of South and Southeast Asia. *FIELD CROPS RESEARCH* 220, 67–77 (2018).
423. Asumadu, H., Ewool, M. B. & Obeng-Antwi, K. Participatory evaluation of drought tolerant maize varieties using mother-baby trial model: A case study in the forest-Savannah transition zone of Ghana. *Journal of Agronomy* 10, 68–73 (2011).
424. Virk, D., Chakraborty, M., Ghosh, J. & Harris, D. Participatory evaluation of horsegram (Macrotyloma uniflorum) varieties and their on-station responses to on-farm seed priming in eastern India. *EXPERIMENTAL AGRICULTURE* 42, 411–425 (2006).
425. Kindie, Y. & Nigusie, Z. Participatory evaluation of lentil varieties in Wag-lasta, Eastern Amhara. *Cogent Food and Agriculture* 4, (2018).
426. Ferede, M. & Demsie, Z. Participatory evaluation of malt barley (Hordium disticum L.) varieties in barley-growing highland areas of Northwestern Ethiopia. *COGENT FOOD & AGRICULTURE* 6, (2020).
427. Chakle, S., Tewolde, F. & Mamo, D. Participatory Evaluation of Open Pollinated Maize (Zea mays L.) Varieties for Green Cob Production Under Irrigation in the North Shewa Lowlands, Ethiopia. *ADVANCES IN AGRICULTURE* 2022, (2022).
428. Harahagazwe, D. *et al. Participatory Evaluation of Resilient Potato Varieties in Climate-Smart Villages of Lushoto in Tanzania*. https://cgspace.cgiar.org/handle/10568/79454 (2016).
429. De Santis, G. *et al.* Participatory Evaluation of Rice Varieties for Specific Adaptation to Organic Conditions in Italy. *SUSTAINABILITY* 14, (2022).
430. Jalleta, T. Participatory evaluation of the performance of some improved bread wheat (Triticum aestivum) varieties in the Jijiga plains of eastern Ethiopia. *Experimental Agriculture* 40, 89–97 (2004).
431. Ouma, J. O. *et al.* Participatory Farmer Evaluation of Stem borer Resistant Maize varieties in three maize growing ecologies of Kenya. 11 (2010) doi:10.22004/ag.econ.96189.
432. Ouma, J. *et al.* Participatory farmer evaluation of stem borer tolerant maize varieties in three maize growing ecologies of Kenya. *AFRICAN JOURNAL OF AGRICULTURAL RESEARCH* 6, 3021–3028 (2011).
433. Bruno, A. *et al.* Participatory farmers' selection of common bean varieties (Phaseolus vulgaris L.) under different production constraints. *Plant Breeding* 137, 283–289 (2018).
434. Belay, B. & Wale, M. Participatory on farm evaluation of improved sorghum varieties in North Gondar areas of Ethiopia. *COGENT FOOD & AGRICULTURE* 7, (2021).
435. Kamara, A. Y. *et al.* Participatory on-farm evaluation of the performance of drought-tolerant maize varieties in the Guinea savannas of Nigeria. *Journal of Food, Agriculture and Environment* 4, 192–196 (2006).

436. Virk, D. S., Singh, D. N., Kumar, R. P., Gangwar, J. S. & Witcombe, J. R. *Participatory plant breeding (PPB) in rice in Eastern India - the success of an NGO/GO partnership*. https://scholar.google.com/scholar_lookup?title=Participatory+plant+breeding+%28PPB%29+in+rice+in+Eastern+India+-+the+success+of+an+NGO%2FGO+partnership&author=Virk%2C+D.+S&publication_year=2002 (2002).
437. Casals, J., Rull, A., Segarra, J., Schober, P. & Simo, J. Participatory Plant Breeding and the Evolution of Landraces: A Case Study in the Organic Farms of the Collserola Natural Park. *AGRONOMY-BASEL* 9, (2019).
438. Ojwang', P., Melis, R., Songa, J., Githiri, M. & Bett, C. Participatory plant breeding approach for host plant resistance to bean fly in common bean under semi-arid Kenya conditions. *EUPHYTICA* 170, 383–393 (2009).
439. Virk, D. S. Participatory plant breeding in finger millet in India. Phase 1.
440. Witcombe, J., Joshi, A. & Goyal, S. Participatory plant breeding in maize: A case study from Gujarat, India. *EUPHYTICA* 130, 413–422 (2003).
441. Kumar, R. *et al.* Participatory plant breeding in rice in Eastern India. in (Consultative Group on International Agricultural Research, 2001).
442. Joshi, K. D., Sthapit, B., Subedi, M. & Witcombe, J. R. *Participatory plant breeding in rice in Nepal. Farmers, scientists and plant breeding: Integrating knowledge and practice* (2002).
443. Ceccarelli, S., Grando, S. & Baum, M. Participatory plant breeding in water-limited environments. *EXPERIMENTAL AGRICULTURE* 43, 411–435 (2007).
444. Witcombe, J. *et al.* Participatory plant breeding is better described as highly client-oriented plant breeding. I. Four indicators of client-orientation in plant breeding. *EXPERIMENTAL AGRICULTURE* 41, 299–319 (2005).
445. Witcombe, J., Gyawali, S., Sunwar, S., Sthapit, B. & Joshi, K. Participatory plant breeding is better described as highly client-oriented plant breeding. II. Optional farmer collaboration in the segregating generations. *EXPERIMENTAL AGRICULTURE* 42, 79–90 (2006).
446. Participatory Plant Breeding Toolkit. https://www.fao.org/plant-treaty/tools/toolbox-for-sustainable-use/details/en/c/1071291/.
447. Assefa, T. *et al.* Participatory Plant Breeding with Traders and Farmers for White Pea Bean in Ethiopia. *Journal of Agricultural Education and Extension* 20, 497–512 (2014).
448. ICRISAT, -. Participatory Plant Improvement. in 1–78 (1998).
449. Ortiz, O., Thiele, G., Nelson, R. & Bentley, J. W. Participatory research (PR) at CIP with potato farming systems in the andes: Evolution and prospects. in *The Potato Crop: Its Agricultural, Nutritional and Social Contribution to Humankind* 451–473 (2019). doi:10.1007/978-3-030-28683-5_13.
450. Sthapit, B. R., Joshi, K. D. & Witcombe, J. R. Participatory rice breeding in Nepal. *Crop Improvement* 23, 179–188 (1996).
451. Craufurd, P. *et al.* Participatory Rice Variety Improvement in Ghana II.
452. Olaoye, G., Ajala, S. & Adedeji, S. Participatory selection of a maize (Zea mays L.) variety for the control of stem borers in a southeastern Nigeria location. *JOURNAL OF FOOD AGRICULTURE & ENVIRONMENT* 7, 508–512 (2009).
453. Mncwango, N., Van Jaarsveld, C., Ntuli, N. & Mavengahama, S. Participatory Selection of Amaranthus Genotypes in the KwaMbonambi Area, KwaZulu-Natal, South Africa. *SUSTAINABILITY* 13, (2021).
454. Dibi, K. *et al.* Participatory selection of orange-fleshed sweetpotato varieties in north

and north-east Cote d'Ivoire. *OPEN AGRICULTURE* 2, 83–90 (2017).
455. Chughtai, S. R., Fateh, J., Munawwar, M. H. & Hussain, M. Participatory sorghum varietal evaluation and selection in Pakistan. *Acta Agronomica Hungarica* 55, 19–26 (2007).
456. Campanelli, G. *et al.* Participatory tomato breeding for organic conditions in Italy. *EUPHYTICA* 204, 179–197 (2015).
457. Elmyhun, M. & Mekonen, M. Participatory varietal evaluation of open pollinated maize in Western Amhara, North-west Ethiopia. *Journal of Crop Science and Biotechnology* 19, 131–136 (2016).
458. Sheeba, A., Mohan, S., Banumathy, S. & Agila, R. Participatory varietal selection (PVS)-a client oriented breeding approach in mung bean (Vigna radiata L.). *Electronic Journal of Plant Breeding* 10, 1441–1447 (2019).
459. Rahman, M. A. *et al.* Participatory varietal selection (PVS): A "bottom-up" breeding approach helps rice farmers in the ayeyarwady delta, myanmar. *Sabrao Journal of Breeding and Genetics* 47, 299–314 (2015).
460. Horn, L., Ghebrehiwot, H., Sarsu, F. & Shimelis, H. Participatory varietal selection among elite cowpea genotypes in northern Namibia. *LEGUME RESEARCH* 40, 995–1003 (2017).
461. Aristya, V., Trisyono, Y., Mulyo, J. & Taryono, T. Participatory Varietal Selection for Promising Rice Lines. *SUSTAINABILITY* 13, (2021).
462. Halaswamy, B. H., Gowda, B. T. S., Seetharam, A., Virk, D. S. & Witcombe, J. R. Participatory varietal selection in finger millet. in (Consultative Group on International Agricultural Research, 2001).
463. Mehraj, U., Ahmad, M., Abidi, I., Gul-Zaffar & Shikari, A. Participatory varietal selection in fodder oats under temperate conditions of Kashmir valley. *INDIAN JOURNAL OF GENETICS AND PLANT BREEDING* 76, 217–220 (2016).
464. Mulatu, E. & Belete, K. Participatory varietal selection in lowland sorghum in eastern Ethiopia: Impact on adoption and genetic diversity. *EXPERIMENTAL AGRICULTURE* 37, 211–229 (2001).
465. Rana, B. S. *et al.* Participatory varietal selection in rabi sorghum in India. in (Consultative Group on International Agricultural Research, 2001).
466. Joshi, K. & Witcombe, J. Participatory varietal selection in rice in Nepal in favourable agricultural environments - A comparison of two methods assessed by varietal adoption. *EUPHYTICA* 127, 445–458 (2002).
467. Mali, S. S., Virk, D. S., Singh, K. B. & Witcombe, J. R. Participatory varietal selection in rice in the Punjab. in (Consultative Group on International Agricultural Research, 2001).
468. Shanthy, T. Participatory varietal selection in sugarcane. *SUGAR TECH* 12, 1–4 (2010).
469. Scurrah, M., Ccanto, R. & Bonierbale, M. *Participatory varietal selection in the Andes Farmer involvement in selecting potatoes with traits from wild relatives. FARMERS AND PLANT BREEDING: CURRENT APPROACHES AND PERSPECTIVES* (2020).
470. Ghosh, R. *et al.* Participatory varietal selection of chickpea in rainfed rice fallow lands of Chhattisgarh and Madhya Pradesh in India for sustainable crop production. *INTERNATIONAL JOURNAL OF PLANT PRODUCTION* 8, 243–254 (2014).
471. Balcha, A. & Tigabu, R. Participatory varietal selection of common bean (Phaseolus vulgaris L.) in Wolaita, Ethiopia. *Asian Journal of Crop Science* 7, 295–300 (2015).
472. Bajgai, Y. *et al.* Participatory varietal selection of potato and agronomic performance with farmers' feedback on new varieties. *Bhutanese Journal of Agriculture* (2018).
473. Haan, S. de *et al. Participatory varietal selection of potato using the mother & baby trial design: A gender-responsive trainer's guide.* (International Potato Center, 2019).

474. Thapa, D. *et al.* Participatory Varietal Selection of Wheat for Micro-Niches of Kathmandu Valley. *JOURNAL OF SUSTAINABLE AGRICULTURE* 33, 745–756 (2009).
475. Prasad, V. L., Bezkorowajnyj, P. G., Nigam, S. N., Hanson, J. & Romney, D. Participatory Varietal Selection to Multiple Actor Orientation–Case study of groundnut in Anantapur, Andhra Pradesh. in 1–18 (2006).
476. Omanya, G. *et al.* Participatory varietal selection with improved pearl millet in West Africa. *EXPERIMENTAL AGRICULTURE* 43, 5–19 (2007).
477. Joshi, K. D. & Witcombe, J. R. Participatory varietal selection, food security, and varietal diversity in a high-potential production system in Nepal. in (Consultative Group on International Agricultural Research, 2001).
478. Walker, T. S. *Participatory Varietal Selection, Participatory Plant Breeding, and Varietal Change*. https://openknowledge.worldbank.org/handle/10986/9182 (2006).
479. Participatory varietal selection: Improved varieties of chickpea for rainfed conditions in Western India. https://teca.apps.fao.org/teca/fr/technologies/4442.
480. SONGYIKHANGSUTHOR, K., ATLIN, G., PHENGCHANH, S. & LINQUIST, B. Participatory varietal selection: Lessons learned from the Lao upland programme. (2002).
481. Participatory varietal selection: short duration vegetable crops for rainfed rabi in India and Nepal. https://www.fao.org/teca/fr/technologies/4584.
482. vom Brocke, K. *et al.* Participatory variety development for sorghum in Burkina Faso: Farmers' selection and farmers' criteria. *FIELD CROPS RESEARCH* 119, 183–194 (2010).
483. Nkongolo, K. K., Chinthu, K. K. L., Malusi, M. & Vokhiwa, Z. *Participatory variety selection and characterization of Sorghum (Sorghum bicolor (L.) Moench) elite accessions from Malawian gene pool using farmer and breeder knowledge*. http://www.academicjournals.org/AJAR/PDF/pdf%202008/April/Nkongolo%20et%20al.pdf (2008).
484. Magaisa, A., Manjeru, P., Kamutando, C. & Moyo, M. Participatory variety selection and stability of agronomic performance of advanced sorghum lines in Zimbabwe. *JOURNAL OF CROP IMPROVEMENT* doi:10.1080/15427528.2021.1974635.
485. Henry, F. O. *et al.* Participatory Variety Selection for enhanced promotion and adoption of improved finger millet varieties: A case for Singida and Iramba Districts in Central Tanzania. *African Journal of Rural Development (AFJRD)* (2017) doi:10.22004/ag.econ.263302.
486. Amare, K. & Kassahun, A. Participatory variety selection for released white common bean varieties in South Gondar Zone, Ethiopia. *HELIYON* 7, (2021).
487. Belay, G. *et al.* Participatory variety selection in the Ethiopian cereal tef (Eragrostis tef). *EXPERIMENTAL AGRICULTURE* 42, 91–101 (2006).
488. Tigist, S. G., Melis, R., Sibiya, J., Amelework, B. & Keneni, G. Participatory variety selection of common bean (Phaseolus vulgaris L.) genotypes in the major bean producing areas of Ethiopia. *Australian Journal of Crop Science* 14, 1055–1063 (2020).
489. Belayneh, D. & Chondie, Y. Participatory variety selection of groundnut (Arachis hypogaea L.) in Taricha Zuriya district of Dawuro Zone, southern Ethiopia. *HELIYON* 8, (2022).
490. Chimonyo, V. G. P., Mutengwa, C. S., Chiduza, C. & Tandzi, L. N. PARTICIPATORY VARIETY SELECTION OF MAIZE GENOTYPES IN THE EASTERN CAPE PROVINCE OF SOUTH AFRICA. *South African Journal of Agricultural Extension* 47, 103–117 (2019).
491. Kolech, S. A., Jong, W. D., Perry, K., Halseth, D. & Mengistu, F. Participatory variety selection: A tool to understand farmers' potato variety selection criteria. *Open Agriculture* 2, 453–463 (2017).

492. Ortiz-Ferrara, G. *et al.* Partnering with farmers to accelerate adoption of new technologies in South Asia to improve wheat productivity. *EUPHYTICA* 157, 399–407 (2007).
493. Long, L. E., Kaiser, C. & Brewer, L. J. Partnering with producers and consumers to enhance cultivar and rootstock selections. in *VII International Cherry Symposium* vol. 1161 221–229 (2017).
494. McGuire, S. Path-dependency in plant breeding: Challenges facing participatory reforms in the Ethiopian sorghum improvement program. *AGRICULTURAL SYSTEMS* 96, 139–149 (2008).
495. Okoro, J. *et al.* Perceived Factors Influencing Farmers' Preference for Rice Varieties in Enugu State, Nigeria. *JOURNAL OF AGRICULTURAL EXTENSION* 27, 86–93 (2023).
496. Rana, R. K. *et al.* PERCEPTION OF GUJARAT FARMERS ON HEAT-TOLERANT POTATO VARIETIES. *Potato Journal (Shimla)* 38, 121–129 (2011).
497. Sardar, A. *et al.* Perceptual characterisation for quality assessment of jackfruit (Artocarpus lieterophyllus) in Terai districts of West Bengal. *INDIAN JOURNAL OF AGRICULTURAL SCIENCES* 89, 433–438 (2019).
498. Goa, Y., Worku, W., Mohammed, H. & Urage, E. PERFORMANCE AND FARMERS PARTICIPATORY SELECTION OF COWPEA VARIETIES IN SOUTHERN ETHIOPIA. *Tropical and Subtropical Agroecosystems* 25, (2022).
499. Sewenet, H. K., Anley, A. M. & Getie, M. A. Performance evaluation and participatory varietal selection of improved bread wheat (Triticum aestivum L.) varieties, the case of Debre Elias District, Northwestern Ethiopia. *Ecological Genetics and Genomics* 19, (2021).
500. Lamessa, K. Performance Evaluation of Banana Varieties, through Farmer's Participatory Selection. *INTERNATIONAL JOURNAL OF FRUIT SCIENCE* 21, 768–778 (2021).
501. Steyn, W., Manning, N., Muller, M. & Human, J. Physical, Sensory and Consumer Analysis of Eating Quality and Appearance of Pear Genotypes among South African Consumers. in vol. 909 579–586 (2011).
502. Ayenan, M. A. T., Ofori, K., Ahoton, L. E. & Danquah, A. Pigeonpea [(Cajanus cajan (L.) Millsp.)] production system, farmers' preferred traits and implications for variety development and introduction in Benin. *Agriculture and Food Security* 6, (2017).
503. Kumara Charyulu, D. *et al.* Pigeonpea Baseline and Early Adoption Surveys in South Asia, Insights from TL-II (Phase 1) Project in India. http://oar.icrisat.org/8354/ (2014).
504. Yohane, E. *et al.* Pigeonpea production constraints and farmers' trait preferences in Malawi: implications for variety design. *SOUTH AFRICAN JOURNAL OF PLANT AND SOIL* 38, 326–337 (2021).
505. Brouwer, B., Murphy, K. & Jones, S. Plant breeding for local food systems: A contextual review of end-use selection for small grains and dry beans in Western Washington. *RENEWABLE AGRICULTURE AND FOOD SYSTEMS* 31, 172–184 (2016).
506. KOUAME, C., Kouassi, N., dri, D. & Amani, N. Plantain (Musa spp., AAB genome) Cultivar Preference, Local Processing Techniques And Consumption Patterns Of Plantain Based Foods Most Consumed In Urban Area Of Abidjan, Côte d'Ivoire. (2015).
507. Constantino, L. V. *et al.* Post-harvest quality and sensory analysis of 'Prata' bananas produced in different cultivation field locations. *Anais da Academia Brasileira de Ciencias* 94, e20201479-Article No.: e20201479 (2022).
508. Kolech, S. *et al.* Potato Variety Diversity, Determinants and Implications for Potato Breeding Strategy in Ethiopia. *AMERICAN JOURNAL OF POTATO RESEARCH* 92, 551–566 (2015).

509. Buijs, J., Martinet, M., de Mendiburu, F. & Ghislain, M. Potential adoption and management of insect-resistant potato in Peru, and implications for genetically engineered potato. *Environmental Biosafety Research* 4, 179–188 (2005).
510. Asante, B. *et al.* Preference for improved varietal attributes of Bambara groundnut among smallholder farmers in Ghana. *AFRICAN JOURNAL OF AGRICULTURAL AND RESOURCE ECONOMICS-AFJARE* 16, 155–168 (2021).
511. Bonany, J. *et al.* Preference mapping of apple varieties in Europe. *FOOD QUALITY AND PREFERENCE* 32, 317–329 (2014).
512. Suwansri, S., Meullenet, J.-F., Hankins, J. A. & Griffin, K. Preference mapping of domestic/imported Jasmine rice for U.S.-Asian consumers. *Journal of Food Science* 67, 2420–2431 (2002).
513. Parth, S., Ardeshna, N., Suvagiya, D. & Swaminathan, B. Preferences for Technological Attributes of Pigeonpea Farmers in Saurashtra Region (Gujarat): A Conjoint Analysis. *INDIAN JOURNAL OF ECONOMICS AND DEVELOPMENT* 14, 133–137 (2018).
514. Sanchez-Toledano, B., Cuevas-Reyes, V., Kallas, Z. & Zegbe, J. Preferences in 'Jalapeno' Pepper Attributes: A Choice Study in Mexico. *FOODS* 10, (2021).
515. Uazire, A. *et al.* Preliminary Evaluation Of Improved Banana Varieties In Mozambique. *African Crop Science Journal (ISSN: 1021-9730) Vol 16 Num 1* 16, (2010).
516. Mudege, N. *et al.* Prioritising quality traits for gender-responsive breeding for boiled potato in Uganda. *INTERNATIONAL JOURNAL OF FOOD SCIENCE AND TECHNOLOGY* 56, 1362–1375 (2021).
517. Leon, L., de la Rosa, R. & Arriaza, M. Prioritization of olive breeding objectives in Spain: Analysis of a producers and researchers survey. *Spanish Journal of Agricultural Research* 19, e0701-Article No.: e0701 (2021).
518. Mafouasson, H. N. A. *et al.* Production Constraints, Farmers' Preferred Characteristics of Maize Varieties in the Bimodal Humid Forest Zone of Cameroon and Their Implications for Plant Breeding. *Agricultural Research* 9, 497–507 (2020).
519. Bahtiar *et al.* Promoting the New Superior Variety of National Hybrid Maize: Improve Farmer Satisfaction to Enhance Production. *AGRICULTURE-BASEL* 13, (2023).
520. Mausch, K., Almekinders, C., Hambloch, C. & McEwan, M. Putting diverse farming households' preferences and needs at the centre of seed system development. *OUTLOOK ON AGRICULTURE* 50, 356–365 (2021).
521. Lupin, B. & Rodriguez, E. M. Quality attributes and socio-demographic factors affecting channel choices. 26 (2012) doi:10.22004/ag.econ.126372.
522. Chijioke, U. *et al.* Quality attributes of fufu in South-East Nigeria: guide for cassava breeders. *INTERNATIONAL JOURNAL OF FOOD SCIENCE AND TECHNOLOGY* 56, 1247–1257 (2021).
523. Charoenthaikij, P., Chaovanalikit, A., Uan-On, T. & Waimaleongora-ek, P. Quality of different rice cultivars and factors influencing consumer willingness-to-purchase rice. *International Journal of Food Science & Technology* 56, (2021).
524. Bellon, M. R. & Reeves, J. Quantitative Analysis of Data from Participatory Methods in Plant Breeding. (2002).
525. Sall, S., Norman, D. & Featherstone, A. Quantitative assessment of improved rice variety adoption: the farmer's perspective. *AGRICULTURAL SYSTEMS* 66, 129–144 (2000).
526. Tiwari, T., Virk, D. & Sinclair, F. Rapid gains in yield and adoption of new maize varieties for complex hillside environments through farmer participation I. Improving options through participatory varietal selection (PVS). *FIELD CROPS RESEARCH* 111, 137–143 (2009).

527. Tiwari, T. *et al.* Rapid gains in yield and adoption of new maize varieties for complex hillside environments through farmer participation. II. Scaling-up the adoption through community-based seed production (CBSP). *FIELD CROPS RESEARCH* 111, 144–151 (2009).
528. Joshi, K. *et al.* Regulatory reform of seed systems: Benefits and impacts from a mungbean case study in Nepal. *FIELD CROPS RESEARCH* 158, 15–23 (2014).
529. Virk, D. *et al.* REML IS AN EFFECTIVE ANALYSIS FOR MIXED MODELLING OF UNBALANCED ON-FARM VARIETAL TRIALS. *EXPERIMENTAL AGRICULTURE* 45, 77–91 (2009).
530. Fiamohe, R., Ndindeng, S., Mujawamariya, G., Sanyang, S. & Futakuchi, K. Responding to consumers' preference in African rice markets: experiences of Africa Rice Center. 14 (2016) doi:10.22004/ag.econ.249322.
531. Dury, S. *et al.* Retail market prices of fonio reveal the demand for quality characteristics in Bamako, Mali. 22 (2007) doi:10.22004/ag.econ.7949.
532. Sperling, L., Loevinsohn, M. E. & Ntabomvura, B. Rethinking the farmer's role in plant breeding: Local bean experts and on-station selection in rwanda. *Experimental Agriculture* 29, 509–519 (1993).
533. SPERLING, L., LOEVINSOHN, M. & NTABOMVURA, B. RETHINKING THE FARMERS ROLE IN PLANT-BREEDING - LOCAL BEAN EXPERTS AND ON-STATION SELECTION IN RWANDA. *EXPERIMENTAL AGRICULTURE* 29, 509–519 (1993).
534. Cairns, J. E. *et al.* Revisiting strategies to incorporate gender-responsiveness into maize breeding in southern Africa. *Outlook on Agriculture* 51, 178–186 (2022).
535. Goddard, J. *et al.* Root, Tuber, and Banana Textural Traits A Review of the Available Food Science and Consumer Preferences Literature. https://www.semanticscholar.org/paper/Root%2C-Tuber%2C-and-Banana-Textural-Traits-A-Review-of-Goddard-Harris/f18224c43e735e6d6e366e263cbbbf61f55d9890 (2015).
536. Iezzoni, A. *et al.* RosBREED: bridging the chasm between discovery and application to enable DNA-informed breeding in rosaceous crops. *HORTICULTURE RESEARCH* 7, (2020).
537. Lyon, A., Silva, E., Zystro, J. & Bell, M. Seed and Plant Breeding for Wisconsin's Organic Vegetable Sector: Understanding Farmers' Needs. *AGROECOLOGY AND SUSTAINABLE FOOD SYSTEMS* 39, 601–624 (2015).
538. Kwambai, T. *et al.* Seed Quality and Variety Preferences Amongst Potato Farmers in North-Western Kenya: Lessons for the Adoption of New Varieties. *POTATO RESEARCH* (2023) doi:10.1007/s11540-023-09626-8.
539. Chupungco, A., Elazegui, D. & Nguyen, M. Seed System, Production and Marketing of Eggplant in Three Major Producing Provinces in the Phiippines. *PHILIPPINE JOURNAL OF CROP SCIENCE* 36, 37–47 (2011).
540. Abera, A. & Karamura, D. Selection criteria of Musa cultivars through a farmer participatory appraisal survey in Uganda. *Experimental Agriculture* 38, 29–38 (2002).
541. Kamara, A., Defoer, T. & De Groote, H. Selection of new varieties through participatory research, the case of corn in South Mali. *Tropicultura* 14, 100–105 (1996).
542. Park, T. A. & Florkowski, W. J. Selection of Peach Varieties and the Role of Quality Attributes. *Journal of Agricultural and Resource Economics* 14 (2003) doi:10.22004/ag.econ.30724.
543. Junior, S. S. *et al.* Selection of thermotolerant Italian tomato cultivars with high fruit yield and nutritional quality for the consumer taste grown under protected cultivation. *Scientia Horticulturae (Amsterdam)* 291, 110559-Article No.: 110559 (2022).

544. Oyunga-Ogubi, M. A., Loechl, C., Quedraogo, H. & Low, J. W. Sensory evaluation and consumer acceptability of orange-fleshed sweetpotato by pregnant women and children < 2 years in western Kenya. in (2012).
545. Dzomeku, B., Kodjo, D., Bam, R. & Ankomah, A. A. Sensory Evaluation of Four FHIA Tetraploid Hybrids for Kaakle (a Local Dish) in Ghana. *Journal of Plant Sciences* 2, 640–643 (2007).
546. Fla State Hort Soc *et al.* Sensory Evaluation of Red and Yellow Grape Tomato Varieties. in vol. 121 178–182 (2008).
547. Dzomeku, B., M, O.-O., A.A, A., E, A. & Kodjo, D. Sensory Evaluation of Some Cooking Bananas in Ghana. *Journal of Applied Sciences* 6, (2006).
548. Ahomondji, S. E. *et al.* Sensory preference criteria and willingness to adopt vegetable soybean “Edamame” in Benin (West Africa). *Journal of Sensory Studies* 38, (2023).
549. Rozzanigo, E., Stiletto, A., Lomolino, G., Vincenzi, S. & Trestini, S. Sensory preferences for pomegranate arils in Italy: A comparison between different varieties and cultivation sites. *FLAVOUR AND FRAGRANCE JOURNAL* 36, 477–489 (2021).
550. Menezes Ayres, E. M., Lee, S. M., Boyden, L. & Guinard, J.-X. Sensory Properties and Consumer Acceptance of Cantaloupe Melon Cultivars. *Journal of Food Science* 84, 2278–2288 (2019).
551. Sinesio, F. *et al.* Sensory Quality of Fresh French and Dutch Market Tomatoes: A Preference Mapping Study with Italian Consumers. *Journal of Food Science* 75, S55–S67 (2010).
552. Rimlinger, A. *et al.* Shifting perceptions, preferences and practices in the African fruit trade: the case of African plum (Dacryodes edulis) in different cultural and urbanization contexts in Cameroon. *JOURNAL OF ETHNOBIOLOGY AND ETHNOMEDICINE* 17, (2021).
553. KD Joshi *et al.* Short duration rice varieties for the High Barind Tract of Bangladesh: the Initial impact of varieties from client oriented breeding and selection in Nepal. (2004).
554. Dermail, A. *et al.* Simultaneous Selection of Sweet-Waxy Corn Ideotypes Appealing to Hybrid Seed Producers, Growers, and Consumers in Thailand. *AGRONOMY-BASEL* 12, (2022).
555. Bellon, M. & Risopoulos, J. Small-scale farmers expand the benefits of improved maize germplasm: A case study from Chiapas, Mexico. *WORLD DEVELOPMENT* 29, 799–811 (2001).
556. Fisher, M. & Snapp, S. SMALLHOLDER FARMERS’ PERCEPTIONS OF DROUGHT RISK AND ADOPTION OF MODERN MAIZE IN SOUTHERN MALAWI. *EXPERIMENTAL AGRICULTURE* 50, 533–548 (2014).
557. Mutanyagwa, A. P. SMALLHOLDER FARMERS’ PREFERENCES FOR IMPROVED MAIZE SEEDS VARIETIES IN TANZANIA. 87 (2017) doi:10.22004/ag.econ.265536.
558. Gamboa, C., Van den Broeck, G. & Maertens, M. Smallholders’ Preferences for Improved Quinoa Varieties in the Peruvian Andes. *SUSTAINABILITY* 10, (2018).
559. Manzanilla, D. *et al.* SOCIAL AND GENDER PERSPECTIVES IN RICE BREEDING FOR SUBMERGENCE TOLERANCE IN SOUTHEAST ASIA. *EXPERIMENTAL AGRICULTURE* 50, 191–215 (2014).
560. Beye, A. *et al.* Socio-Economic Constraints of Adopting New Cowpea Varieties in Three Agro-Ecological Zones in the Senegalese Peanut Basin. *SUSTAINABILITY* 14, (2022).
561. Mwamahonje, A. *et al.* Sorghum Production Constraints, Trait Preferences, and Strategies to Combat Drought in Tanzania. *SUSTAINABILITY* 13, (2021).
562. Ahmad Yahaya, M., Shimelis, H., Nebie, B., Ojiewo, C. O. & Danso-Abbeam, G.

Sorghum production in Nigeria: opportunities, constraints, and recommendations. *Acta Agriculturae Scandinavica Section B: Soil and Plant Science* 72, 660–672 (2022).
563. Wanga, M., Shimelis, H. & Mengistu, G. Sorghum Production in Northern Namibia: Farmers' Perceived Constraints and Trait Preferences. *SUSTAINABILITY* 14, (2022).
564. Park, J. & Henneberry, S. R. South Korean Millers' Preferences for the Quality Characteristics of Hard White Wheat that is Used in Producing All-purpose Flour. 21 (2010) doi:10.22004/ag.econ.56327.
565. IOP, Aristya, V., Taryono, Trisyono, Y. & Mulyo, J. Stakeholder preferences on major characteristics of promising rice lines. in vol. 686 (2021).
566. Basu, D., Basu, G., Biswas, S. & Goswami, R. Stakeholders' Perceived Attributes of Fresh Vegetables: an Important Input in Breeding Program. in vol. 895 51–57 (2011).
567. Osei, M. K., Danquah, A., Blay, E., Danquah, E. & Adu-Dapaah, H. Stakeholders‟ Perception and Preferences of Post-harvest Quality Traits of Tomato in Ghana. *Sustainable Agriculture Research* (2018) doi:10.22004/ag.econ.301832.
568. Craine, E., Bramwell, S., Ross, C., Fisk, S. & Murphy, K. Strategic malting barley improvement for craft brewers through consumer sensory evaluation of malt and beer. *JOURNAL OF FOOD SCIENCE* 86, 3628–3644 (2021).
569. Matala, V. Strawberry variety trials on berry farms. in 215–217 (2002). doi:10.17660/ActaHortic.2002.567.43.
570. Sarangi, S. *et al.* SUB1 varieties increased rice (Oryza saliva) yield in flood-prone rainfed lowlands of coastal regions. *INDIAN JOURNAL OF AGRICULTURAL SCIENCES* 90, 2064–2070 (2020).
571. Manzanilla, D. *et al.* Submergence risks and farmers' preferences: Implications for breeding Sub1 rice in Southeast Asia. *AGRICULTURAL SYSTEMS* 104, 335–347 (2011).
572. Tena, E., Mekbib, F., Shimelis, H. & Mwadzingeni, L. Sugarcane production under smallholder farming systems: Farmers preferred traits, constraints and genetic resources. *Cogent Food and Agriculture* 2, (2016).
573. Li, Z. *et al.* Supporting Successful Transition to the Fresh Market: Research and Extension Needs of Pacific Northwest Strawberry Growers. *HORTTECHNOLOGY* 29, 649–658 (2019).
574. Houngue, J. *et al.* Survey of farmers' knowledge of cassava mosaic disease and their preferences for cassava cultivars in three agro-ecological zones in Benin. *JOURNAL OF ETHNOBIOLOGY AND ETHNOMEDICINE* 14, (2018).
575. Agapie, O. *et al.* SURVEY ON CONSUMERS PREFERENCE IN THE NEW ACCLIMATIZED SPECIE IN ROMANIA: BENINCASA HISPIDA. *SCIENTIFIC PAPERS-SERIES B-HORTICULTURE* 66, 391–396 (2022).
576. Singh, S. K., Kumar, S. & Sah, A. K. S. and U. Sustainable Improvement in Pulses Productivity in Hamirpur District of Uttar Pradesh : An Approach of Farmer Participatory Varietal Evaluation. *Not Available* (2008).
577. Kagimbo, F., Shimelis, H. & Sibiya, J. Sweet Potato Weevil Damage, Production Constraints, and Variety Preferences in Western Tanzania: Farmers' Perception. *Journal of Crop Improvement* 32, 107–123 (2018).
578. Rana, R. *et al.* Tackling moisture stress with drought-tolerant potato (Solanum tuberosum) varieties: Perception of Karnataka farmers. *INDIAN JOURNAL OF AGRICULTURAL SCIENCES* 83, 216–222 (2013).
579. Britwum, K. & Demont, M. Tailoring rice varieties to consumer preferences induced by cultural and colonial heritage: Lessons from New Rice for Africa (NERICA) in The Gambia. *OUTLOOK ON AGRICULTURE* 50, 305–314 (2021).

580. Kumara charyulu, D. & Moses Shyam, D. *Targeting of Grain Legumes for Income and Nutritional Security in South Asia*. (LAP LAMBERT Academic Publishing is a trademark of International Book Market Service Ltd. member of OmniScriptum Publishing Group, 2019).
581. Khanal, N., Harris, D., Sherpa, L., Giri, R. & Joshi, K. Testing and promotion of mungbean in cereal fallows in the low hills and terai agroecosystems of Nepal. in 255–267 (2004).
582. Craufurd, P. *et al.* TESTING DROUGHT-TOLERANT PLANT TYPES OF UPLAND RICE IN GHANA USING PARTICIPATORY METHODS DEPARTMENT OF INTERNATIONAL DEVELOPMENT (DFID) PLANT SCIENCES RESEARCH PROGRAMME PROJECT R6826. (2000).
583. IOP *et al.* The analysis of selection of upland rice variety in East Aceh District. in vol. 425 (2020).
584. Botero, H., Barnes, A., Perez, L., Rios, D. & Ramirez-Villegas, J. The determinants of common bean variety selection and diversification in Colombia. *ECOLOGICAL ECONOMICS* 190, (2021).
585. Barkley, A. & Porter, L. The determinants of wheat variety selection in Kansas, 1974 to 1993. *AMERICAN JOURNAL OF AGRICULTURAL ECONOMICS* 78, 202–211 (1996).
586. Akankwasa, K. *et al.* The East African highland cooking bananas 'Matooke' preferences of farmers and traders: Implications for variety development. *INTERNATIONAL JOURNAL OF FOOD SCIENCE AND TECHNOLOGY* 56, 1124–1134 (2021).
587. Effah-Manu, L. *et al.* The effect of gender on end-user preferences for yam quality descriptors. *FOOD SCIENCE & NUTRITION* 10, 3890–3904 (2022).
588. BELLON, M. THE ETHNOECOLOGY OF MAIZE VARIETY MANAGEMENT - A CASE-STUDY FROM MEXICO. *HUMAN ECOLOGY* 19, 389–418 (1991).
589. Bucheyeki, T., Shenkalwa, M., Mapunda, X. & Matata, W. The groundnut client oriented research in Tabora, Tanzania. *AFRICAN JOURNAL OF AGRICULTURAL RESEARCH* 5, 356–362 (2010).
590. Derese, S. A., Shimelis, H., Laing, M. & Mengistu, F. The impact of drought on sorghum production, and farmer's varietal and trait preferences, in the north eastern Ethiopia: implications for breeding. *Acta Agriculturae Scandinavica Section B Soil and Plant Science* 68, 424–436 (2018).
591. Cole, J. The Impact of Personal Attitudes on Cereal Variety Adoption Decisions in Alberta. 2007, 25 (2007).
592. Ashby, J. & Polar, V. *The implications of gender relations for modern approaches to crop improvement and plant breeding. GENDER, AGRICULTURE AND AGRARIAN TRANSFORMATIONS: CHANGING RELATIONS IN AFRICA, LATIN AMERICA AND ASIA* (2019).
593. Chiwona-Karltun, L. *et al.* The importance of being bitter—a qualitative study on cassava cultivar preference in Malawi. *Ecology of Food and Nutrition* 37, 219–245 (1998).
594. Carpenter, D. The in situ conservation of rice plant genetic diversity: A case study from a Philippine barangay. *AGRICULTURE AND HUMAN VALUES* 22, 421–434 (2005).
595. Faye, M. D., Jooste, A., Lowenberg-DeBoer, J. & Fulton, J. R. The influence of cowpea characteristics on cowpea prices in Senegal. 43, 12 (2004).
596. Mohapatra, S., Gregorio, G. & Kumar, A. The pillars of Africa's agriculture. *Appropriate Technology* 38, 45–46 (2011).
597. Jesionkowska, K., Konopacka, D. & Plocharski, W. The quality of apples - preferences among consumers from Skierniewice, Poland. *Journal of Fruit and Ornamental Plant Research* 14, 173–182 (2006).

598. PRUDENCIO, C., ORKWOR, G. & KISSIEDU, A. THE RELATIONSHIPS BETWEEN CASSAVA VARIETY SET CHARACTERISTICS, FARMERS FOOD SECURITY OBJECTIVES, ENVIRONMENTAL AND SOCIOECONOMIC CONDITIONS IN AFRICA. *AGRICULTURAL SYSTEMS* 39, 387–408 (1992).
599. Singh, R. K. *et al.* The right rice in the right place: Systematic exchange and farmer-based evaluation of rice germplasm for salt-affected areas. in *Tropical Deltas and Coastal Zones: Food Production, Communities and Environment at the Land-Water Interface* 166–182 (2010).
600. Casals, J. *et al.* The risks of success in quality vegetable markets: Possible genetic erosion in Marmande tomatoes (Solanum lycopersicum L.) and consumer dissatisfaction. *SCIENTIA HORTICULTURAE* 130, 78–84 (2011).
601. Aw-Hassan, A., Mazid, A. & Salahieh, H. The role of informal farmer-to-farmer distribution in diffusion of new barley varieties in Syria. *EXPERIMENTAL AGRICULTURE* 44, 413–431 (2008).
602. Otieno, Z., Okello, J. J., Nyikal, R., Mwang'ombe, A. & Clavel, D. The role of varietal traits in the adoption of improved dryland crop varieties: The case of pigeon pea in Kenya. 06, 18 (2011).
603. Naico, A. & Lusk, J. The Value of a Nutritionally Enhanced Staple Crop: Results from a Choice Experiment Conducted with Orange-fleshed Sweet Potatoes in Mozambique. *JOURNAL OF AFRICAN ECONOMIES* 19, 536–558 (2010).
604. Ndjeunga, J. & Nelson, C. Toward understanding household preference for consumption characteristics of millet varieties: a case study from western Niger. *AGRICULTURAL ECONOMICS* 32, 151–165 (2005).
605. Vernooy, R. *et al.* Towards new roles, responsibilities and rules: the case of participatory plant breeding. in 613–628 (2009).
606. Britwum, K. & Demont, M. Trading off consumer preferences induced by cultural and colonial heritage: Lessons from New Rice for Africa (NERICA) in Casamance, Senegal. *Q Open* 1, (2021).
607. Marenya, P., Wanyama, R., Alemu, S. & Woyengo, V. Trait preference trade-offs among maize farmers in western Kenya. *HELIYON* 7, (2021).
608. Adu, G. *et al.* Trait profile of maize varieties preferred by farmers and value chain actors in northern Ghana. *AGRONOMY FOR SUSTAINABLE DEVELOPMENT* 41, (2021).
609. Tyack, N. *et al. Twenty years of participatory varietal selection at AfricaRice Lessons from farmer involvement in variety development*. *FARMERS AND PLANT BREEDING: CURRENT APPROACHES AND PERSPECTIVES* (2020).
610. Teeken, B. *et al.* Understanding cassava varietal preferences through pairwise ranking of gari-eba and fufu prepared by local farmer-processors. *INTERNATIONAL JOURNAL OF FOOD SCIENCE AND TECHNOLOGY* 56, 1258–1277 (2021).
611. Kolech, S. A., De Jong, W., Halseth, D. & Schulz, S. Understanding farmer needs and unlocking local genetic resources for potato improvement: A case study in Ethiopia. *African Journal of Food, Agriculture, Nutrition and Development* 19, 13883–13905 (2019).
612. Melesse, M., Tirra, A., Ojiewo, C. & Hauser, M. Understanding Farmers' Trait Preferences for Dual-Purpose Crops to Improve Mixed Crop-Livestock Systems in Zimbabwe. *SUSTAINABILITY* 13, (2021).
613. Maligalig, R., Demont, M., Umberger, W. & Peralta, A. Understanding Filipino Rice Farmer Preference Heterogeneity for Varietal Trait Improvements: A Latent Class Analysis. *JOURNAL OF AGRICULTURAL ECONOMICS* 72, 134–157 (2021).
614. Varshney, D., Joshi, P., Roy, D. & Kumar, A. Understanding the Adoption of Modern

Cultivars in India: Adoption Probability and Use Intensity. *JOURNAL OF AGRICULTURAL AND RESOURCE ECONOMICS* 47, 167–189 (2022).
615. Basavaraj, G. *et al.* Understanding trait preferences of farmers for post-rainy sorghum and pearl millet in India - A conjoint analysis. *Indian Journal of Agricultural Economics* 70, 130–143 (2015).
616. Hairmansis, A. *et al. Upland rice breeding lines adapted to high elevation areas selected through participatory approaches*. (SABRAO Journal of Breeding & Genetics, 2017).
617. Lado, J., Rivas, F., Moltini, A., Alcaire, F. & Ares, G. Uruguayan consumers' perception of mandarins insights for selection and marketing of new cultivars. *AGROCIENCIA URUGUAY* 25, (2021).
618. Zhao, S. *et al.* US Peach Producer Preference and Willingness to Pay for Fruit Attributes. *HORTSCIENCE* 52, 116–121 (2017).
619. Li, Z. *et al.* US Southeastern Peach Growers Preferences for Fruit Size and External Color versus Resistance to Brown Rot Disease. *HORTTECHNOLOGY* 30, 576–584 (2020).
620. Cliff, M., Stanich, K., Lu, R. & Hampson, C. Use of descriptive analysis and preference mapping for early-stage assessment of new and established apples. *JOURNAL OF THE SCIENCE OF FOOD AND AGRICULTURE* 96, 2170–2183 (2016).
621. Cliff, M., Sanford, K., Wismer, W. & Hampson, C. Use of digital images for evaluation of factors responsible for visual preference of apples by consumers. *Hortscience* 37, 1127–1131 (2002).
622. Arora, D. & García, M. A. *Use of discrete choice experiments in gendered participatory breeding and varietal selection: A case study of biofortified rice varietal selection in Bolivar, Colombia*. https://cgspace.cgiar.org/handle/10568/103661 (2019).
623. Kamara, A. *et al.* Using a Participatory Approach and Legume Integration to Increase the Productivity of Early Maturing Maize in the Nigerian Sudan Savannas. *INTERNATIONAL JOURNAL OF AGRONOMY* 2019, (2019).
624. Kern, S. *et al.* Using choice analysis of growers' preferences to prioritize breeding traits in horticultural tree crops: A macadamia case study. *SCIENTIA HORTICULTURAE* 294, (2022).
625. Baidu-Forson, J., Ntare, B. R. & Waliyar, F. Utilizing conjoint analysis to design modern crop varieties: empirical example for groundnut in Niger. *Agricultural Economics: The Journal of the International Association of Agricultural Economists* 8 (1997) doi:10.22004/ag.econ.174070.
626. Carneiro, R. *et al.* Utilizing Consumer Perception of Edamame to Guide New Variety Development. *FRONTIERS IN SUSTAINABLE FOOD SYSTEMS* 4, (2021).
627. Kafiriti, E., Dondeyne, S., Msomba, S., Deckers, J. & Raes, D. Variations in agronomic characteristics of irrigated rice varieties: Lessons from participatory trials in South Eastern Tanzania. *JOURNAL OF FOOD AGRICULTURE & ENVIRONMENT* 1, 273–277 (2003).
628. Yadav, H. K., Singh, S., Kumar, V. & Kumara, A. Varietal Preferences and Adoption Pattern of Economically Viable Medicinal and Aromatic Crops by the Indian Farmers. *AGRIS on-line Papers in Economics and Informatics* 7 (2013) doi:10.22004/ag.econ.148107.
629. Mengue Efanden, C., Temple, L. & Tomekpé, K. Varietal selection by growers in central Cameroon. *Infomusa (English ed.)* (2003).
630. Teeken, B. & Temudo, M. Varietal selection in marginal agroecological niches and cultural landscapes: the case of rice in the Togo Hills. *AGROECOLOGY AND SUSTAINABLE FOOD SYSTEMS* 45, 1109–1138 (2021).
631. Nayak, Y. P. S. A. K. *et al.* Varietal selection in sodic soils of Indo-Gangetic plains

through farmers' participatory approach. *Not Available* (2013).
632. Hintze, L., Renkow, M. & Sain, G. Variety characteristics and maize adoption in Honduras. *AGRICULTURAL ECONOMICS* 29, 307–317 (2003).
633. Macholdt, J. & Honermeier, B. Variety choice in crop production for climate change adaptation: Farmer evidence from Germany. *OUTLOOK ON AGRICULTURE* 45, 117–123 (2016).
634. Edmeades, S., Smale, M., Renkow, M. & Phaneuf, D. J. VARIETY DEMAND WITHIN THE FRAMEWORK OF AN AGRICULTURAL HOUSEHOLD MODEL WITH ATTRIBUTES: THE CASE OF BANANAS IN UGANDA. 68 (2004) doi:10.22004/ag.econ.60323.
635. Rubyogo, J.-C. & Odhiambo, W. Variety selection and seed quality management in grain legume cultivation. in (Burleigh Dodds Science Publishing Limited, 2018).
636. Osewe, M., Liu, A. & Han, J. Variety Traits and Sustainable Food Security: The Role of Improved Cassava Varieties in Kenya. *Chemical Engineering Transactions* 89, 355–360 (2021).
637. Singh, B. *et al.* Vegetable varieties with multiple attributes spread at faster rate - A case study in popularizing carrot variety Pusa Rudhira in NCR Region. *INDIAN JOURNAL OF HORTICULTURE* 75, 482–491 (2018).
638. Timsina, K. P. & Shivakoti, G. P. Vegetables production and marketing: Practice and perception of vegetable seed producers and fresh growers in Nepal. *Agriculture and Food Security* 7, (2018).
639. Camarena-Gomez, D. M. & Sanjuan, A. I. Walnut Preferences in Spain: Is the Spanish Consumer Ready for New Varieties? 15 (2005) doi:10.22004/ag.econ.24749.
640. Bairagi, S., Demont, M., Custodio, M. & Ynion, J. What drives consumer demand for rice fragrance? Evidence from South and Southeast Asia. *BRITISH FOOD JOURNAL* 122, 3473–3498 (2020).
641. Bishaw, Z., Struik, P. & van Gastel, A. Wheat and barley seed system in Syria: farmers' varietal perceptions, seed sources and seed management. *INTERNATIONAL JOURNAL OF PLANT PRODUCTION* 5, 323–347 (2011).
642. Nazli, H., Birol, E., Asare-Marfo, D. & Tariq, A. Wheat Farmers' Preferences for Wheat Traits in Punjab, Pakistan: A Choice Experiment Approach. 23 (2015) doi:10.22004/ag.econ.211352.
643. Jaradat, A. A. Wheat Landraces: A mini review. *Emirates Journal of Food and Agriculture* 25, 20–29 (2013).
644. Nigus, M., Shimelis, H., Mathew, I. & Abady, S. Wheat production in the highlands of Eastern Ethiopia: opportunities, challenges and coping strategies of rust diseases. *Acta Agriculturae Scandinavica Section B: Soil and Plant Science* 72, 563–575 (2022).
645. Bishaw, Z., Struik, P. C. & van Gastel, A. J. G. Wheat seed system in Ethiopia: Farmers' varietal perception, seed sources, and seed management. *Journal of New Seeds* 11, 281–327 (2010).
646. Rasheed, S. *et al.* Who cultivates traditional paddy varieties and why? Findings from Kerala, India. *Current Science (Bangalore)* 121, 1188–1193 (2021).
647. Yokouchi, T. & Saito, K. Why did farmers stop cultivating NERICA upland rice varieties in central Benin? *INTERNATIONAL JOURNAL OF AGRICULTURAL SUSTAINABILITY* 15, 724–734 (2017).
648. Sanya, L. *et al.* Why Gender Matters in Breeding: Lessons from Cooking Bananas in Uganda. *SUSTAINABILITY* 15, (2023).
649. Gallardo, R. K., Kupferman, E. & Colonna, A. Willingness to Pay for Optimal 'Anjou' Pear Quality. *Hortscience* 46, 452–456 (2011).

650. Gallardo, R. K., Lusk, J. L., Holcomb, R. B. & Rayas-Duarte, P. Willingness-to-Pay for Attribute Level and Variability: The Case of Mexican Millers' Demand for Hard Red Winter Wheat. 41, 13 (2009).
651. Kilduff, A. & Tregeagle, D. Willingness-to-Pay for Produce: A Meta-Regression Analysis Comparing the Stated Preferences of Producers and Consumers. *HORTICULTURAE* 8, (2022).
652. WIRFP participatory plant breeding: Concepts and examples. https://www.fao.org/plant-treaty/tools/toolbox-for-sustainable-use/details/en/c/1071263/.
653. Gibson, R., Byamukama, E., Mpembe, I., Kayongo, J. & Mwanga, R. Working with farmer groups in Uganda to develop new sweet potato cultivars: decentralisation and building on traditional approaches. *EUPHYTICA* 159, 217–228 (2008).
654. Mudege, N. N., Mukewa, E. & Amele, A. *Workshop Report: Training on Gender Integrated Potato Participatory Varietal Selection (PVS) in Ethiopia.* https://cgspace.cgiar.org/handle/10568/80906 (2015).
655. Sivapalan, S., Batten, G., Goonetilleke, A. & Kokot, S. Yield performance and adaptation of some Australian-grown rice varieties through multivariate analysis. *Australian Journal of Agricultural Research* 58, 874–883 (2007).
656. Ulzen, O. *et al.* Yield potentials of improved rice varieties for increased lowland rice production within the mankran watershed in Ghana. *PLANT PRODUCTION SCIENCE* 26, 17–27 (2023).
657. Kierczyńska, S. Zróżnicowanie preferencji konsumentów jabłek na przykładzie studentów Uniwersytetu Przyrodniczego w Poznaniu. *Journal of Agribusiness and Rural Development* (2013) doi:10.22004/ag.econ.254080.

<u>The study protocol</u>

Trait prioritization in plant breeding programs: a review on tools and methods

Based on the PRIMSA statement for scoping reviews
Open Science Framework registration

<u>Administrative Information</u>

1. <u>Identification:</u> Scoping review
2. <u>Registration:</u> https://osf.io/ayw8q/
3. <u>Authors:</u>

Martina Occelli (MO), Cornell University – mo386@cornell.edu
Rishabh Mukerjee (RM), Cornell University – rm824@cornell.edu
Elisabeth Garner (EG), Cornell University – eg65@cornell.edu
Miguel Gomez (MG), Cornell University – mig7@cornell.edu
Christian J. Miller (CM), Cornell University – cjm267@cornell.edu
Sergio Puerto (SP), Cornell University – sap257@cornell.edu
Jaron Porciello (JP), Cornell University – jat264@cornell.edu
Hale Ann Tufan (HT), Cornell University – hat36@cornell.edu

<u>Roles and responsibilities of authors</u>

Content expertise: EG, MG, RM, MO, JP, SP, HT
Systematic review methods and information retrieval: CM, RM, MO, JP

Protocol elaboration: MO, CM, JP
Title and abstract review: RM, MO
Full text review: RM, MO
Data extraction: EG, MG, RM, MO, SP, HT
Data synthesis and data charting: RM, MO (with the support of JP for the network analysis)

Introduction and Background

The goal of this article is to review tools and methods currently used to set and rank trait priorities for plant breeding programs. We focus on trait prioritization as it enhances the effectiveness of plant breeding programs by prioritizing variety traits for a targeted selection of parental lines. Often, trait prioritization is led by plant breeders (Ragot et al. 2018); however global challenges of food insecurity and poverty have compelled breeding programs to consider more participatory processes to redefine objectives for social inclusion and impact.

To review how trait prioritization is currently addressed in plant breeding, we perform a scoping review among existing sources. We will look for qualitative and quantitative research that implements tools and methods to operationalize trait prioritization in plant breeding programs. We will scope the results obtained by primary research and reviews, isolating patterns of methods and tools adoption across time, space, crop, institutions and gender.

Methods

A systematic scoping review is designed to thematically characterize the extent, range and nature of existing evidence across a five-step process: identifying the research question; identifying relevant studies; selecting studies; extracting and charting the data; and collating, summarizing, and reporting the results (Arksey & O'Malley, 2005; Tricco et al., 2016).

The following elements were used to conceptualize the research question and the objectives of the study.

Setting/Geographic scope: All geographic regions will be considered for this scoping review. We include any relevant literature that relate to trait priority setting from village - level to regional - level studies or from low and middle – income countries to high – income economies.

Population: Our target population are all stakeholders in the crop value chain (e.g., producers, consumers, processors, dealers, traders), as we are interested in extrapolating tools and methods in which trait prioritization is performed involving end-users.

Interventions/Exposures: We are interested in interventions experiments which led to the elicitation of preferences and priorities by crop producers regarding a new or existing breeding product proposed by researchers. Meta analysis reviews of interventions are also considered as subject of the scoping review.

Defintions

Definitions of the parameters, interventions and exposures of interest in our analysis:

- Trait prioritization: we define trait prioritization as how crop breeding programs establish priorities for ranking traits of a given crop. Establishing priorities in traits, also defined as trait priority setting, is the process of making decisions about how best to allocate limited resources to prioritize a given trait with respect to another trait of the same crop. It is a complex process, as the purposes for choosing a trait are directly linked to the local usage of that crop, to the local agroclimatic zone, to

the targeted end-users and to the agronomic characteristics of the local inputs, donors or policy objectives and resources available (e.g., financial capital, human capital). For this reason, trait prioritization often involves a diverse array of stakeholders, decision-makers, and scientists whose motivations and actions are often imperfectly aligned. Effective trait priority setting addresses these differing interests and motivations through a clear process focused on the use of evidence, transparency, and participation to identify the most appropriate set of variety traits to address population needs (Braunschweig 2000). Trait prioritization can entail a participatory nature: we define as participatory trait prioritization (or trait priority setting) the process through which trait priorities are co-established with farmers. Participatory trait priority setting can take the form of participatory rural appraisal, ranking and rating of traits and stated-choice experiments using multi-criteria decision making.

- Variety trait: characteristic of a given variety which expresses a specific quality of the variety. Traits can have different nature: among others, there are agronomic traits, which are of direct interest for agronomists and help the control of pests, diseases and weeds, nutritional traits, which contribute to the food security of consumers and climatic traits, which support the climate change adaptation of end-users.
- Trait preference: preference stated for a given or multiple variety traits.
- Plant breeding: Plant breeding uses principles from a variety of sciences to improve the genetic potential of plants. The process involves combining parental plants to obtain the next generation with the best characteristics. Breeders improve plants by selecting those with the greatest potential based on performance data, pedigree, and more sophisticated genetic information. Plants are improved for food, feed, fiber, fuel, shelter, landscaping, ecosystems services and a variety of other human activities (Definition by the National Association of Plant Breeders, US. Available at: https://www.plantbreeding.org/content/what-is-plant-breeding/). Within plant breeding, we can distinguish participatory plant breeding. Participatory plant breeding was proposed in early 1980s as a socio-technological solution to variety development, complementary to conventional plant breeding. The main participatory research in the plant breeding domain is called client-oriented plant breeding (COPB), and participatory varietal selection (PVS). In participatory research, clients (mainly farmers, but not solely) are included in all major decisions at all stages of a plant breeding program. The difference between this methodology and PVS is the demarcation when client participation starts; specifically, clients are engaged from the first stage of breeding pipeline in COPB, while in PVS farmers are involved in the testing of lines developed by plant breeders.
- Seed systems: science which studies formal and farmers' seed exchange systems. Formal and farmers' seed systems differ in how they use and maintain plant genetic resources for food and agriculture. Formal systems tend to produce uniform varieties through scientific breeding. Informal systems tend to generate and maintain fewer uniform materials adapted to local requirements (landraces) but also may provide a conduit for exchange of materials derived from modern varieties.
- Agronomy: agronomy is the science and technology of producing and using plants by agriculture for food, fuel, fiber, chemicals, recreation, or land conservation. Agronomy has come to include research of plant genetics, plant physiology, meteorology, and soil science.
- Crop: a crop is a plant that can be grown and harvested extensively for profit or subsistence.
- Seed: The term seed is used here to include any type of planting material that is intended for use in producing a crop, i.e., either generative or vegetative, such as roots, tubers, bulbs, cuttings, rhizomes, and apomictic seed. We consider only cases when human intervention in handling seed is explicit (Louwaars 2011).

- Planting material: Plant materials include a wide and varied group of plant parts from many species. General categories of plant materials include grasses, rushes, barks, woods, gourds, stems, roots, seeds, and leaves. These materials can be used to construct baskets, netting, cordage, and even fabrics.
- Germplasm: germplasm are living genetic resources such as seeds or tissues that are maintained for the purpose of animal and plant breeding.

Research question

1. What tools and methods have been used for trait prioritization in plant breeding programs across time, study location, crop, institutions and gender?

Eligibility criteria

Inclusion

For an article to be included in this study, it must meet all of the following inclusion criteria:

1. Original research (qualitative and quantitative reports) and/or review of existing research including gray literature.
2. Explicit focus or clear relevance on trait prioritization (see definition).
3. Explicit focus on crops, varieties, seed, planting materials or germplasm (see definitions).
4. The study is written in English.
5. The study presents primary data.
6. The study presents meta analysis reviews of interventions.
7. The study include field, on-farm, trials, laboratory experiments, where stakeholders' preferences on traits are elicited.

Exclusion

An article which is excluded will have at least one of the following characteristics:

1. The study does not focus or demonstrate a clear relevance on the topics of trait prioritization.
2. Study in which the concept of trait prioritization is not matched, or it is matched only in cited references or keywords.
3. Study in which trait prioritization or similar definitions appear only in sections related to future works and are not the main subject of the study.
4. The study is not specifically carried out in the domain of plant breeding, seed systems or agronomy.
5. The study has the main text in a language different from English.
6. The study include field experiments, but do not elicit stakeholders' preferences on traits.

Date: We are not considering a specific time frame for our scoping review. Studies meeting the inclusion criteria were published as early as 1970. We concluded the screening of the sources in March 2022, so studies published after that month are not included.

Information sources

We searched commonly used databases in the life sciences and socioeconomic domains for articles related to trait priority setting in plant breeding.

We screened 5 databases for peer-reviewed articles, books, chapters, and conference papers:

(i) Scopus
(ii) Web of Science
(iii) CAB Direct
(iv) AgEcon Search
(v) BIOSIS Citation Index

We do not use Google Scholar, as previous methodological studies have found it inappropriate as a principal search system (Gusenbauer and Haddaway 2020).

We further surveyed the following 12 gray literature sources:

(i) Commonwealth Scientific and Industrial Research Organization (CSIRO)
(ii) Gardian (searches 15 CGIAR websites - https://gardian.bigdata.cgiar.org)
(iii) International Fund for Agricultural Development (IFAD)
(iv) JPAL/ATAI impact evaluations (IPA)
(v) Overseas Development Institute (ODI)
(vi) UK Department for International Development (DFID)
(vii) World Bank
(viii) World Health Organization (WHO)
(ix) United Nations Environment Programme (UNEP)
(x) World Food Programme (WFP)
(xi) Food and Agriculture Organization (FAO)
(xii) AgriLinks (USAID Feed the Future platform)

This list stems partially from the sources used by Acevedo et al. 2020.

Search strategy

A comprehensive strategy was developed to identify all available research on the main topic, trait prioritization in plant breeding programs. Search terms will include variations of the key concepts in the research question. Table 1 presents the search strategy in its entirety, to allow replicability of the results.

Table 1 Search strategy for abstract, as implemented in the Web of Science platform

Row number	Search string
1	TS=(farme* OR household OR supplie* OR consume* OR producer OR trade* OR processo* OR dealer OR student* OR expert* OR seed company OR responde* OR stakeh* OR particip*)
2	TS=((trait OR variet* OR characterist*) NEAR (prefer* OR priorit* OR select* OR adopt*))
3	TS=(((breed* AND (plant OR variet*)) OR crop* OR agronom* OR farm* OR agricultur* or seed*))
4	1 AND 2 AND 3
5	Filter: English, Article, Conference Paper

TS = topic; DE = keywords; AND = find records containing all terms separated by the operator; OR = find records containing any of the terms separated by the operator.

Study records

Searches will be performed across all sources listed in the section on information sources, and search results will be de-duplicated to remove redundant citations identified from multiple sources.

Extraction of records from peer-reviewed sources: the structure of online databases for peer-reviewed articles, books, chapters, and conference papers makes the search strategy (Table 1) objective and transparent.

Extraction of records from gray literature: as structured databases are missing for documents in the gray literature, the search strategy (Table 1) might result in subjective outcomes, according to the author in charge of conducting the search. To mitigate this element of subjectivity, we internally conduct a peer-review process. Following the search strategy in table 1, MO and RM screen the 10 information sources listed at page 4. The two authors will independently report in an excel file the titles, keywords and abstracts of the studies which result from the search strategy. The two excel files are then compared for differences: studies identified by both authors are included into the first repository without any further review process. On the contrary, studies which appeared in one excel file but not in the other are flagged as potential inconsistencies and they are subjected to the review of a third author (alternatively EG, MG, SP or HT). If considered on scope by the third reviewer, the flagged studies are included into the first repository; otherwise, they are discarded.

A peer-review process will be used for title and abstract and full-text screening. Article screening will take place in two phases:

- Phase 1: Title and abstract screening of all de-duplicated citations. Citations will be screened for relevance against the exclusion criteria of this protocol.
- Phase 2: Full-text screening of all articles deemed relevant in the title and abstract screening phase. Reasons for exclusion at this stage will be documented.

In both phases, all citations will be reviewed for relevance by two independent reviewers among authors (MO and RM). Each citation that meets all of the inclusion criteria at the title and abstract and full-text screening phases will be included. Each citation that meets one of the exclusion criteria at the title and abstract and full- text screening phases will be excluded. All conflicts will be resolved by a third, independent reviewer among authors (alternatively EG, MG, SP or HT). This step of the analysis is performed using the Covidence Library Software.

Once the final library for the review is assembled, a data extraction template will be used to collect all the relevant information. The template is presented in Appendix A. The data extraction template is tested by MO and RM before use, and data are extracted by the review team as a whole. Conflicts will be resolved by consensus.

Critical appraisal of individual sources of evidence

Scoping reviews do not aim to produce a critically appraised and synthesized result/answer to a particular question and therefore no formal critical appraisal is conducted for a scoping review.

Data synthesis

Data extraction (Appendix A) drives the data synthesis of the information from studies in the final repository. Both steps are based on categories, which provide evidence to answer the research questions at the center of this scoping review (listed at page 2). The categories are summarized in Table 2.

Table 2 | Summary of categories to operationalize the concept of trait prioritization

Category type	Categories
Trait prioritization taxa	Mention of trait prioritization vs mention of trait preferences

Journal/Publishing organization	Journal/Organization publishing the study
Institution	Institution / CG center in which the trait prioritization exercise was implemented
Donor	Donor providing funding for the trait prioritization exercise
Crop	Specific crop at the center of the study
Variety	Variety of the crop considered
Gender mention	Mention of gender disaggregation possible for the study
Ranking	Trait ranked
Data disaggregation	Trait ranking data disaggregated by any variable (sex, region, age or any other variable specified)
Timeframe	Year in which the trait prioritization experiment has been conducted
Declared breeding purpose	For yield vs for climate vs for nutrition vs food security
Geographic area of the study	Individual country vs regional vs global
Method	Specific method utilized to conduct the trait prioritization
Tool	Specific tool utilized to conduct the trait prioritization
Specific mention of	Local knowledge vs traditional knowledge vs indigenous knowledge
Specific mention of	Priority setting
Degree of end-users' engagement	Participatory vs non – participatory experiment

Data Charting

To summarize findings and provide a clear answer to our question of interest, we will list, explain and chart data by a number of criteria:

- Summary of tools to rank trait preferences
- Summary of methods used to analyze data on trait preferences
- Evolution of tools used to rank trait preferences
- Evolution of the methods used to analyse data on trait preferences
- Share of papers mentioning two key dimensions: participation and priority setting
- Share of papers segmented for social dimensions
- Ranking of trait preferences, disaggregated by time, crop group and segment

- Map of evidence distribution by country and crop groups
- Network of (i) authors, (ii) affiliations and (iii) funding agencies
- Stated objectives of the papers focused on trait prioritization

References

Acevedo, M., Pixley, K., Zinyengere, N., Meng, S., Tufan, H., Cichy, K., Bizikova, L., Isaacs, K., Ghezzi-Kopel, K. and Porciello, J. 2020. A scoping review of adoption of climate-resilient crops by small-scale producers in low-and middle-income countries. *Nature plants*, *6*(10): 1231-1241.

Braunschweig, T. 2000. Priority setting in agricultural biotechnology research: Supporting public decisions in developing countries with the Analytic Hierarchy Process. Research Report N. 16. The Hague: International Service for National Agricultural Research.

Louwaars, N.P., Le Coent, P. and Osborn, T., 2011. Seed systems and plant genetic resources for food and agriculture. FAO.

Ragot, M., Bonierbale, M. and Weltzien, E. 2018. From Market Demand to Breeding Decisions: A Framework. Lima (Peru). CGIAR Gender and Breeding Initiative. GBI Working Paper. No. 2. Available online at: www.rtb.cgiar.org/gender-breeding-initiative.

Tricco, AC, Lillie, E, Zarin, W, O'Brien, KK, Colquhoun, H, Levac, D, Moher, D, Peters, MD, Horsley, T, Weeks, L, Hempel, S et al. PRISMA extension for scoping reviews (PRISMA-ScR): checklist and explanation. *Ann Intern Med*. 2018,169(7):467-473. doi:10.7326/M18-0850.

Appendix A

Data extraction template

Doi / handle – website	
Title	
Year of publication	
Type of publication	Peer – reviewed / grey literature
Journal / Publishing organization	
Objective of the study	Quote from the paper
Novelty of the study	Quote from the paper
Country (ies) of the study	
Admin2 level is included in the study	
Data collection end year	
Crop / germoplasm at the centre of the study	
Varities of the crop considered in the study	
Number of households included in the study	By admin2 / by sex
Multiple residents from the same household	Yes / No
Tools used for the data collection	
Authors consider the study participatory	Word search for participatory
Methods used for analysing data	
Trait ranked	Yes / No
Trait ranking disaggregated by any variable	
Explanation for differences in the rankings by disaggregating variable	Quote from the paper, if any
Funding agency / Donors	
Priority setting mentioned explicitely	Yes / No
Knowledge mentioned explicitly	Yes / No
