# Supplementary material for: A scoping review on tools and methods for trait prioritization in crop breeding programmes
Source: Nat Plants. 2024 Feb 22;10(3):402–11. doi: 10.1038/s41477-024-01639-6 (PMC10954539; doi:10.1038/s41477-024-01639-6)
Supplement: Supplementary file 2 — Reporting Summary [file 41477_2024_1639_MOESM2_ESM.pdf]

nature portfolio

Corresponding author(s): Hale Ann Tufan

Last updated by author(s): January 17th 2024

Reporting Summary

Nature Portfolio wishes to improve the reproducibility of the work that we publish. This form provides structure for consistency and transparency in reporting. For further information on Nature Portfolio policies, see our Editorial Policies and the Editorial Policy Checklist.

Statistics

For all statistical analyses, confirm that the following items are present in the figure legend, table legend, main text, or Methods section.

n/a	Confirmed	
☐	☒	The exact sample size (n) for each experimental group/condition, given as a discrete number and unit of measurement
☒	☐	A statement on whether measurements were taken from distinct samples or whether the same sample was measured repeatedly
☒	☐	The statistical test(s) used AND whether they are one- or two-sided<br>*Only common tests should be described solely by name; describe more complex techniques in the Methods section.*
☒	☐	A description of all covariates tested
☒	☐	A description of any assumptions or corrections, such as tests of normality and adjustment for multiple comparisons
☐	☒	A full description of the statistical parameters including central tendency (e.g. means) or other basic estimates (e.g. regression coefficient) AND variation (e.g. standard deviation) or associated estimates of uncertainty (e.g. confidence intervals)
☒	☐	For null hypothesis testing, the test statistic (e.g. F, t, r) with confidence intervals, effect sizes, degrees of freedom and P value noted<br>*Give P values as exact values whenever suitable.*
☒	☐	For Bayesian analysis, information on the choice of priors and Markov chain Monte Carlo settings
☒	☐	For hierarchical and complex designs, identification of the appropriate level for tests and full reporting of outcomes
☒	☐	Estimates of effect sizes (e.g. Cohen's d, Pearson's r), indicating how they were calculated

Our web collection on statistics for biologists contains articles on many of the points above.

Software and code

Policy information about availability of computer code

Data collection	Upon completion of all searches for the scoping review, we proceeded to upload the records into Covidence Library System (https://www.covidence.org/), where studies were screened over a set of inclusion and exclusion criteria. A peer-review process was used for title and abstract and full-text screening. A data extraction framework of 40 questions supported the inclusion and selection process.
Data analysis	After the extraction phase, data were synthetized descriptively using R (version 4.2.3) and R-studio (R code team, 2021). The network representation of authors, affiliations and donors is carried out in VOSviewer (Van Eck and Waltman 2010). The code utilized for this study is made publicly available and hosted in the following GitHub repository: https://github.com/TufanLab/trait-priority-scoping-review.git

For manuscripts utilizing custom algorithms or software that are central to the research but not yet described in published literature, software must be made available to editors and reviewers. We strongly encourage code deposition in a community repository (e.g. GitHub). See the Nature Portfolio guidelines for submitting code & software for further information.

Data

Policy information about availability of data

All manuscripts must include a data availability statement. This statement should provide the following information, where applicable:
- Accession codes, unique identifiers, or web links for publicly available datasets
- A description of any restrictions on data availability
- For clinical datasets or third party data, please ensure that the statement adheres to our policy

The data utilized for this study is publicly available and hosted in the following GitHub repository https://github.com/TufanLab/trait-priority-scoping-review.git.

Research involving human participants, their data, or biological material

Policy information about studies with human participants or human data. See also policy information about sex, gender (identity/presentation), and sexual orientation and race, ethnicity and racism.

Reporting on sex and gender	N/A
Reporting on race, ethnicity, or other socially relevant groupings	N/A
Population characteristics	N/A
Recruitment	N/A
Ethics oversight	N/A

Note that full information on the approval of the study protocol must also be provided in the manuscript.

Field-specific reporting

Please select the one below that is the best fit for your research. If you are not sure, read the appropriate sections before making your selection.

☐ Life sciences ☒ Behavioural & social sciences ☐ Ecological, evolutionary & environmental sciences

For a reference copy of the document with all sections, see nature.com/documents/nr-reporting-summary-flat.pdf

Behavioural & social sciences study design

All studies must disclose on these points even when the disclosure is negative.

Study description	The study is a scoping review. The reporting guidelines for a systematic scoping review PRISMA-ScR (Preferred Reporting Items for Systematic review and Meta-Analysis Protocols) (Tricco et al. 2018) was used to examine the tools and methods for trait elicitation and ranking over the past forty years in plant breeding programs across time, study location, crop group, institutions, and gender. Systematic scoping reviews are used to explore the evidence in a field to address questions relating to what is known about a topic, what can be synthesized from existing studies to develop policy or practice recommendations, and what aspects of a topic have yet to be addressed by researchers.
Research sample	The data collection protocol was created according to PRISMA-ScR (Preferred Reporting Items for Systematic review and Meta-Analysis Protocols). We searched five electronic databases of scholarly journal articles for a comprehensive retrieval: Scopus, Web of Science, CAB Direct, AgEcon Search, and BIOSIS. A large body of relevant literature was likely to exist outside of scholarly publications, so twelve grey literature sources were searched as well: Commonwealth Scientific and Industrial Research Organization (CSIRO), Gardian, International Fund for Agricultural Development (IFAD), JPAL/ATAI impact evaluations (IPA), Overseas Development Institute (ODI), UK Department for International Development (DFID), World Bank, World Health Organization (WHO), United Nations Environment Programme (UNEP), World Food Programme (WFP), Food and Agriculture Organization (FAO), AgriLinks (USAID Feed the Future platform). While there was significant overlap between some of these grey literature sources, searching all sources was necessary to exhaust the literature. As a search strategy we used keywords and subject headings to discover articles that covered the population and topic identified in the research question. The search strategies and all resources searched are provided in Appendix II of the manuscript. The sample of captured studies is representative of the research space.
Sampling strategy	Inclusion consisted of original research or reviews which explicitly discuss trait prioritization as it relates to crops, varieties, seeds, planting materials, or germplasm. Any articles that reported on research outside of the domains of plant breeding, seed systems, or agronomy (e.g., livestock or non-food crop studies) were excluded. There was no restriction on the search for dates, geographical scope. Only studies published in English were included.
Data collection	A peer-review process was used for title and abstract and full-text screening. Researchers engaged in the data collection were not

Data collection	blind to experimental conditions and study hypotheses. A data extraction framework of 40 questions supported the inclusion and selection process. Extensive pre-testing helped tailoring the set of questions and ask a minimum number of information which could be answered by all papers. The framework is provided in Appendix II of the manuscript, where a full extraction template is reported. Researchers engaged in the data collection were not blinded to experimental conditions and study hypotheses.
Timing	We performed the final searches of all scholarly databases on June 23, 2023. Searches of all grey literature sources took place over the period from March 12, 2022, until July 6, 2022. Upon completion of all searches there were 17,786 records retrieved and uploaded into Covidence for review (https://www.covidence.org/). After deduplication, there were a total of 11,978 unique records. After screening based on title and abstract, 1,325 studies we identified as being potentially relevant to the research topic. We obtained the full text versions of the articles, with each article being reviewed and confirmed as appropriate by the authors. After completing this process, 657 studies were included in the data extraction.
Data exclusions	17,786 records were identified from 5 peer-reviewed databases and 12 gray literature sources. 10.653 records were excluded because considered irrelevant according to the inclusion criteria (see research sample section above). 1,325 full-text records were screened for eligibility. 668 were excluded for the following reasons: 388 because trait prioritization is not at the center of the study (appears only in future works), 94 because end-users were not engaged, 94 because they were not focused on trait prioritization at all, 51 because full text was not available, 19 because they referred to non-food crops, 8 because the main text was not in English, 7 because they were not in the domain of plant breeding, agronomy, or seed systems and finally 7 because they referred to food processing and not varietal traits. The final number of studies included for review was 657.
Non-participation	No participants were involved in the study.
Randomization	This study does not entail allocating participants in experimental groups.

Reporting for specific materials, systems and methods

We require information from authors about some types of materials, experimental systems and methods used in many studies. Here, indicate whether each material, system or method listed is relevant to your study. If you are not sure if a list item applies to your research, read the appropriate section before selecting a response.

Materials & experimental systems

n/a	Involved in the study	
☒	☐	Antibodies
☒	☐	Eukaryotic cell lines
☒	☐	Palaeontology and archaeology
☒	☐	Animals and other organisms
☒	☐	Clinical data
☒	☐	Dual use research of concern
☒	☐	Plants

Methods

n/a	Involved in the study	
☒	☐	ChIP-seq
☒	☐	Flow cytometry
☒	☐	MRI-based neuroimaging

Plants

Seed stocks	N/A
Novel plant genotypes	N/A
Authentication	N/A
